# Supplementary figures and images for: Cryo-sensitive aggregation triggers NLRP3 inflammasome assembly in cryopyrin-associated periodic syndrome (part 1 of 2)
Source: eLife. 2022 May 26;11:e75166. doi: 10.7554/eLife.75166 (PMC9177154; doi:10.7554/eLife.75166)

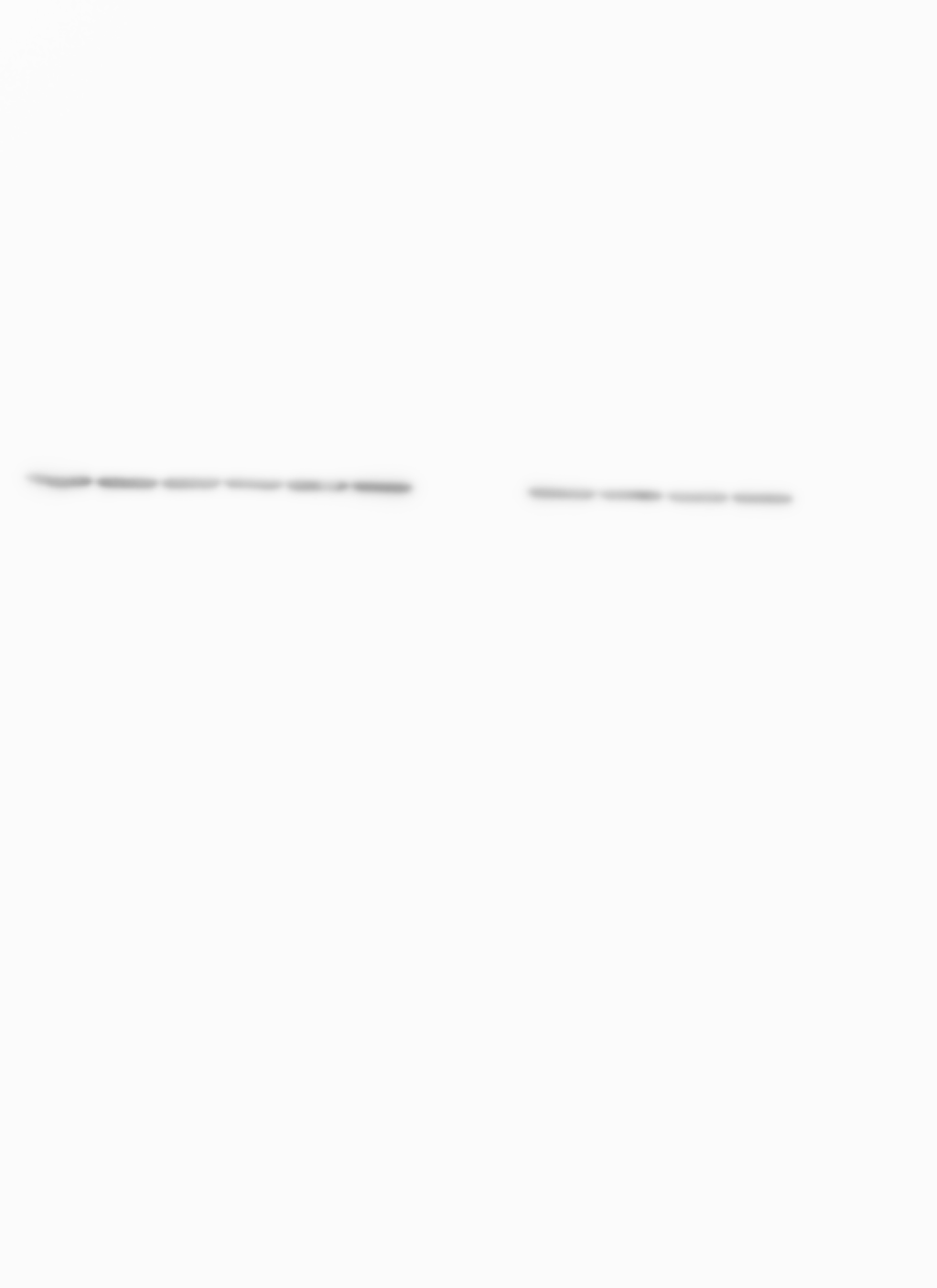

Supplement: Figure 1—figure supplement 1—source data 1. [file elife-75166-fig1-figsupp1-data1.zip › Figure_1- figure_supplemnt_1Source_Data/Fig1_S1B_actin__raw.tif]

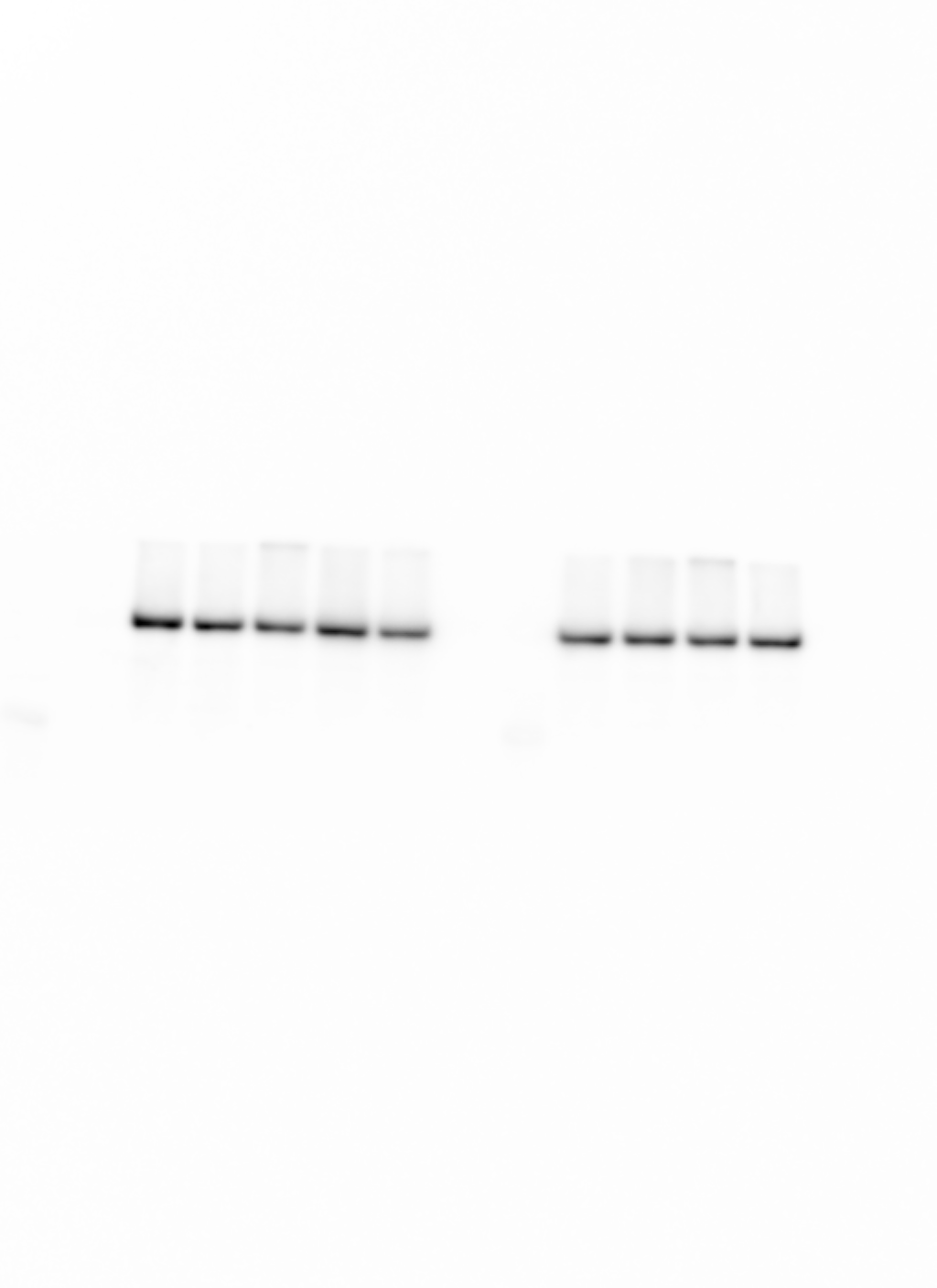

Supplement: Figure 1—figure supplement 1—source data 1. [file elife-75166-fig1-figsupp1-data1.zip › Figure_1- figure_supplemnt_1Source_Data/Fig1_S1B_NLRP3_raw.tif]

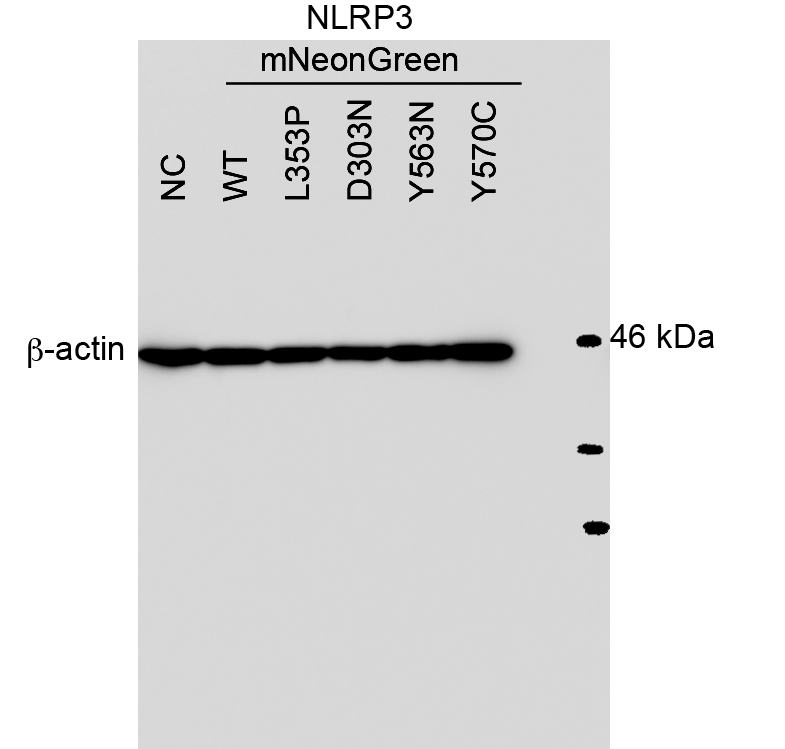

Supplement: Figure 1—figure supplement 1—source data 1. [file elife-75166-fig1-figsupp1-data1.zip › Figure_1- figure_supplemnt_1Source_Data/Fig1_S1Bactin_labeled.tif]

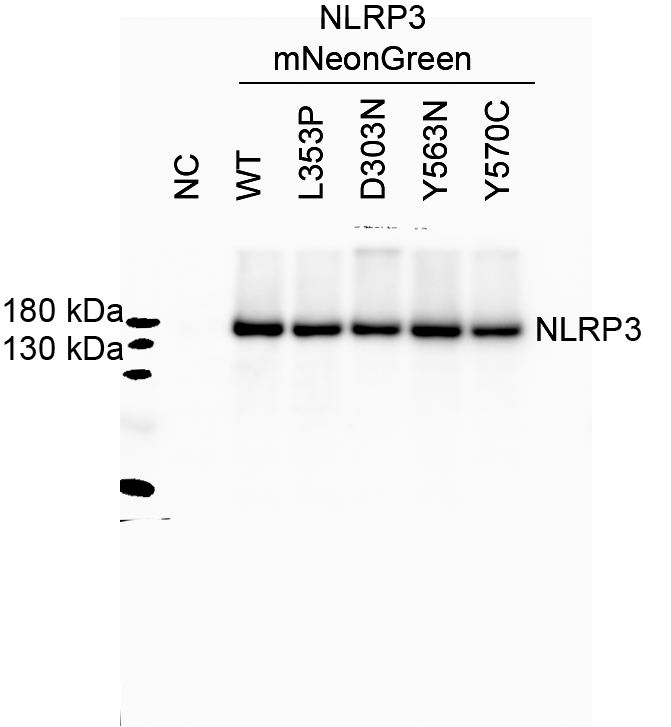

Supplement: Figure 1—figure supplement 1—source data 1. [file elife-75166-fig1-figsupp1-data1.zip › Figure_1- figure_supplemnt_1Source_Data/Fig1_S1BNLRP3_labeled.tif]

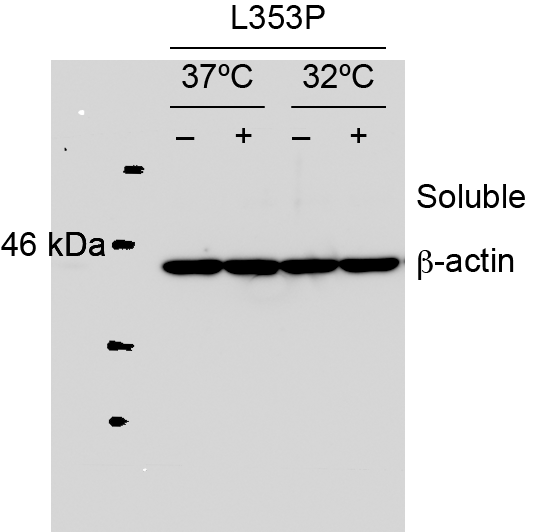

Supplement: Figure 3—source data 1. [file elife-75166-fig3-data1.zip › Figure_3-Source_data_1/Fig3D_actin_labeled.tif]

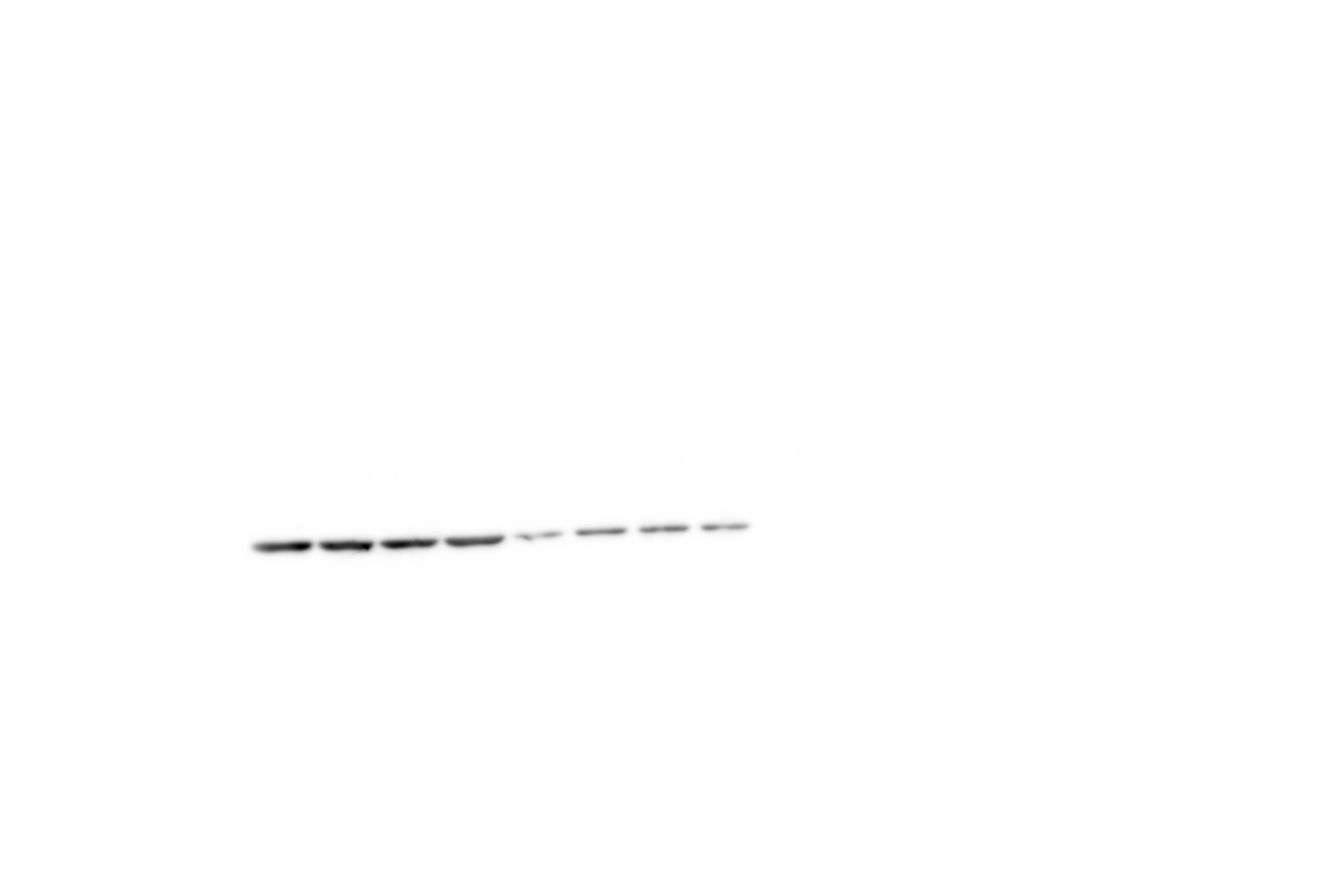

Supplement: Figure 3—source data 1. [file elife-75166-fig3-data1.zip › Figure_3-Source_data_1/Fig3D_actin_raw.tif]

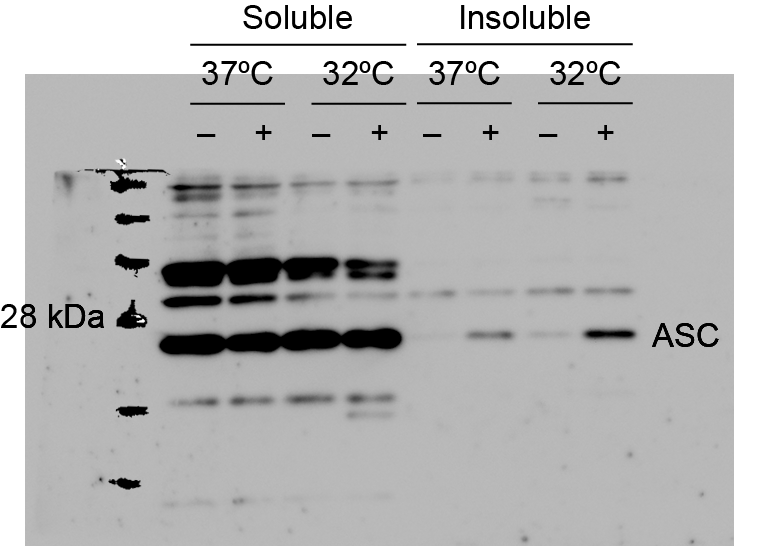

Supplement: Figure 3—source data 1. [file elife-75166-fig3-data1.zip › Figure_3-Source_data_1/Fig3D_ASC_labeled.tif]

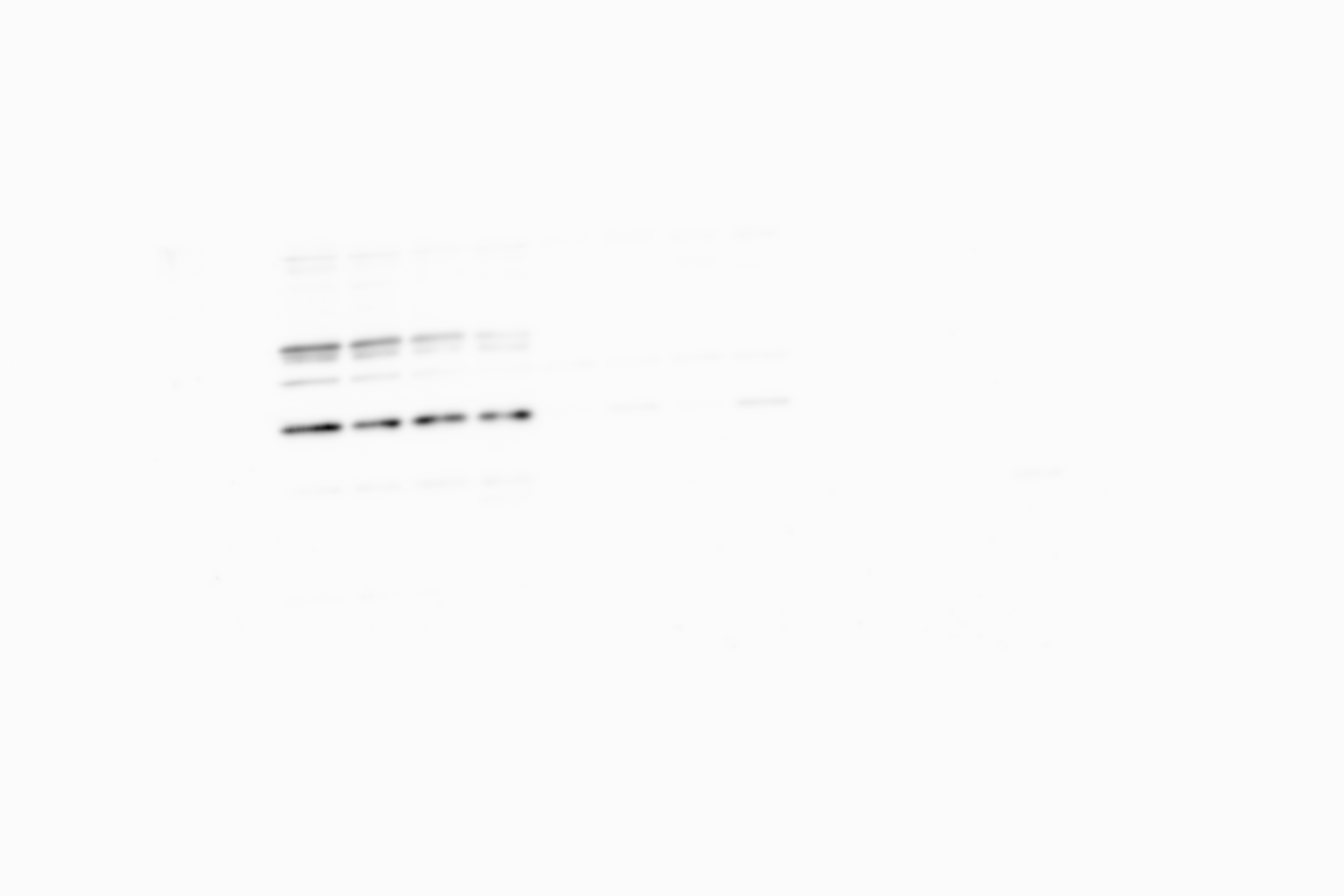

Supplement: Figure 3—source data 1. [file elife-75166-fig3-data1.zip › Figure_3-Source_data_1/Fig3D_ASC_raw.tif]

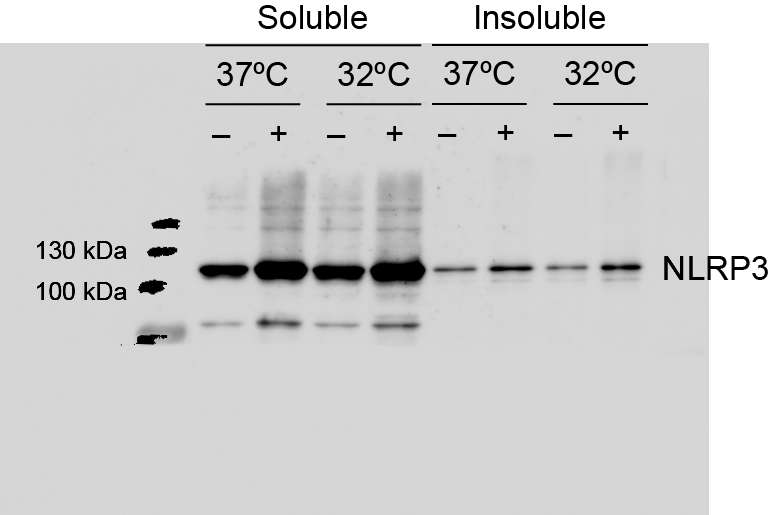

Supplement: Figure 3—source data 1. [file elife-75166-fig3-data1.zip › Figure_3-Source_data_1/Fig3DNLRP3_labeled.tif]

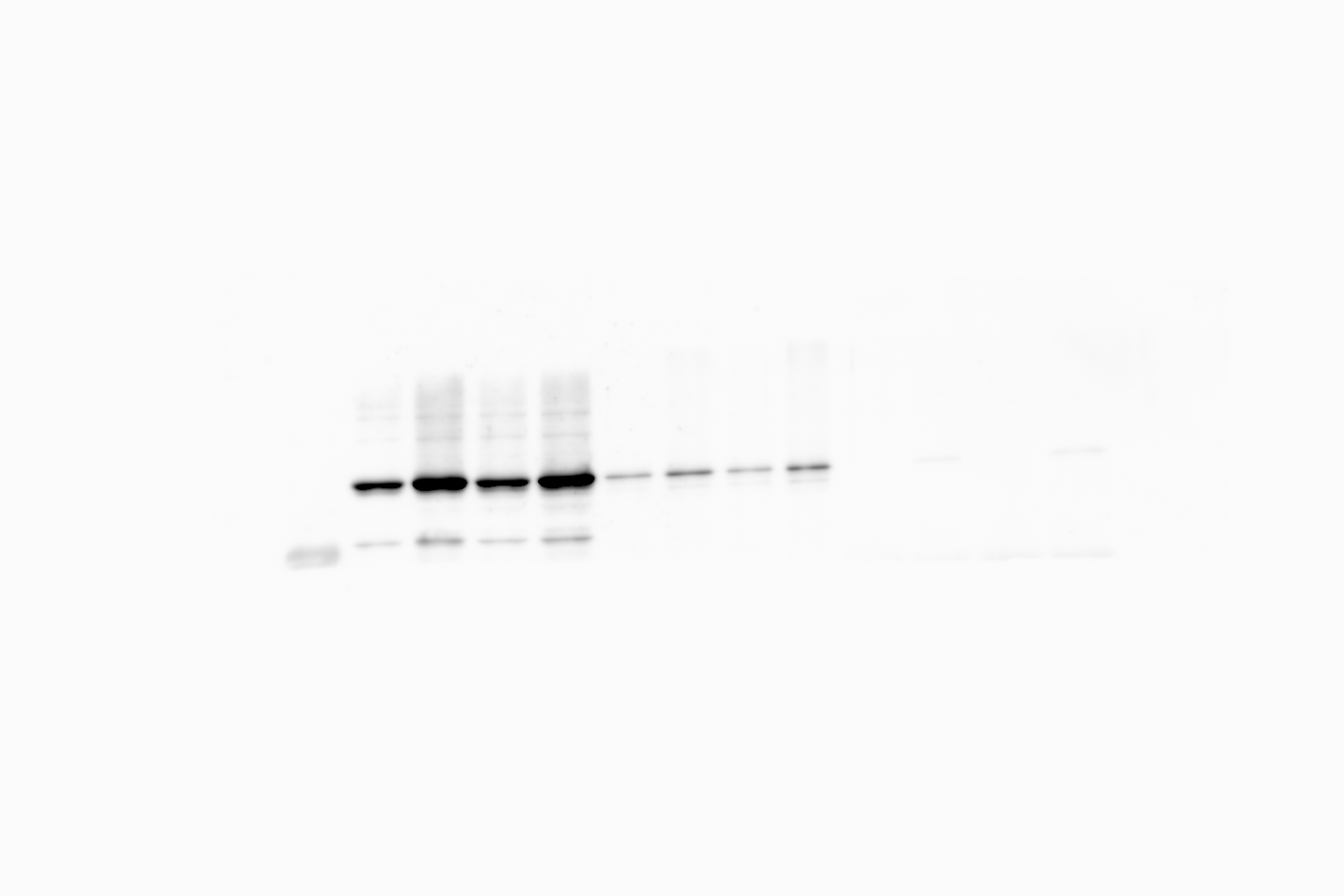

Supplement: Figure 3—source data 1. [file elife-75166-fig3-data1.zip › Figure_3-Source_data_1/Fig3DNLRP3_raw.tif]

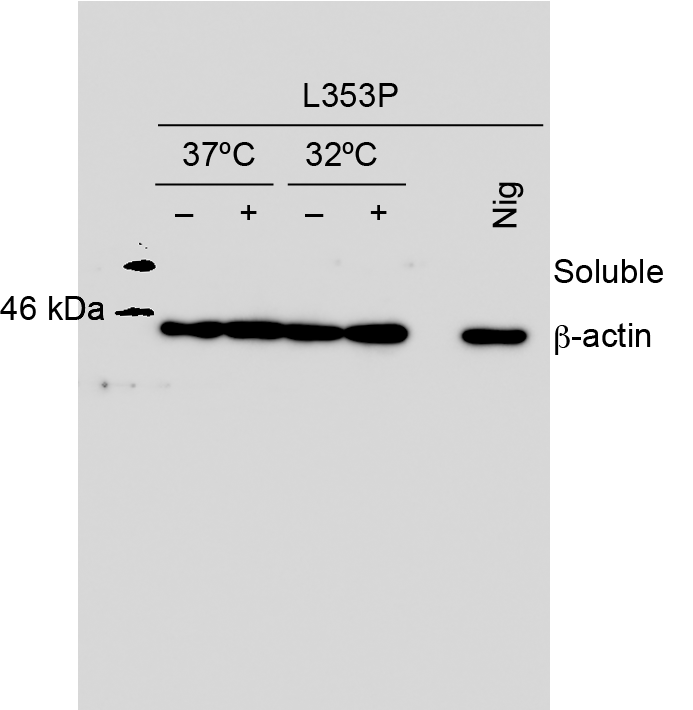

Supplement: Figure 3—source data 2. [file elife-75166-fig3-data2.zip › Figure_3-Source_data_2/Fig3E_actin_labeld.tif]

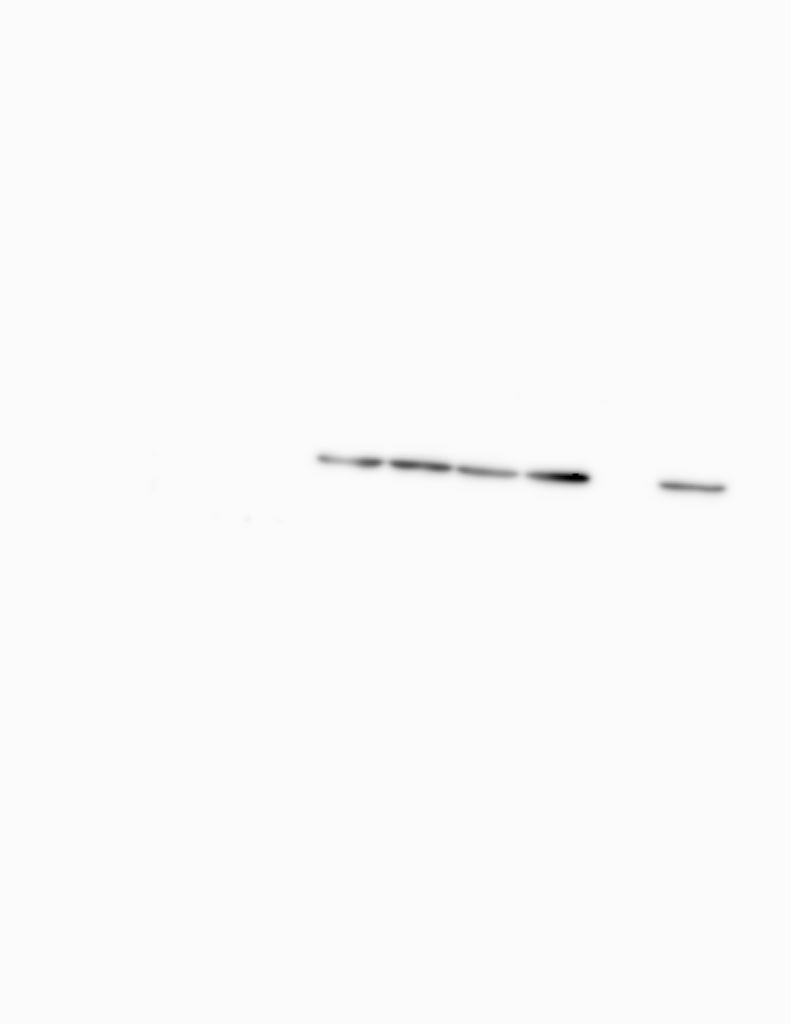

Supplement: Figure 3—source data 2. [file elife-75166-fig3-data2.zip › Figure_3-Source_data_2/Fig3E_actin_raw.tif]

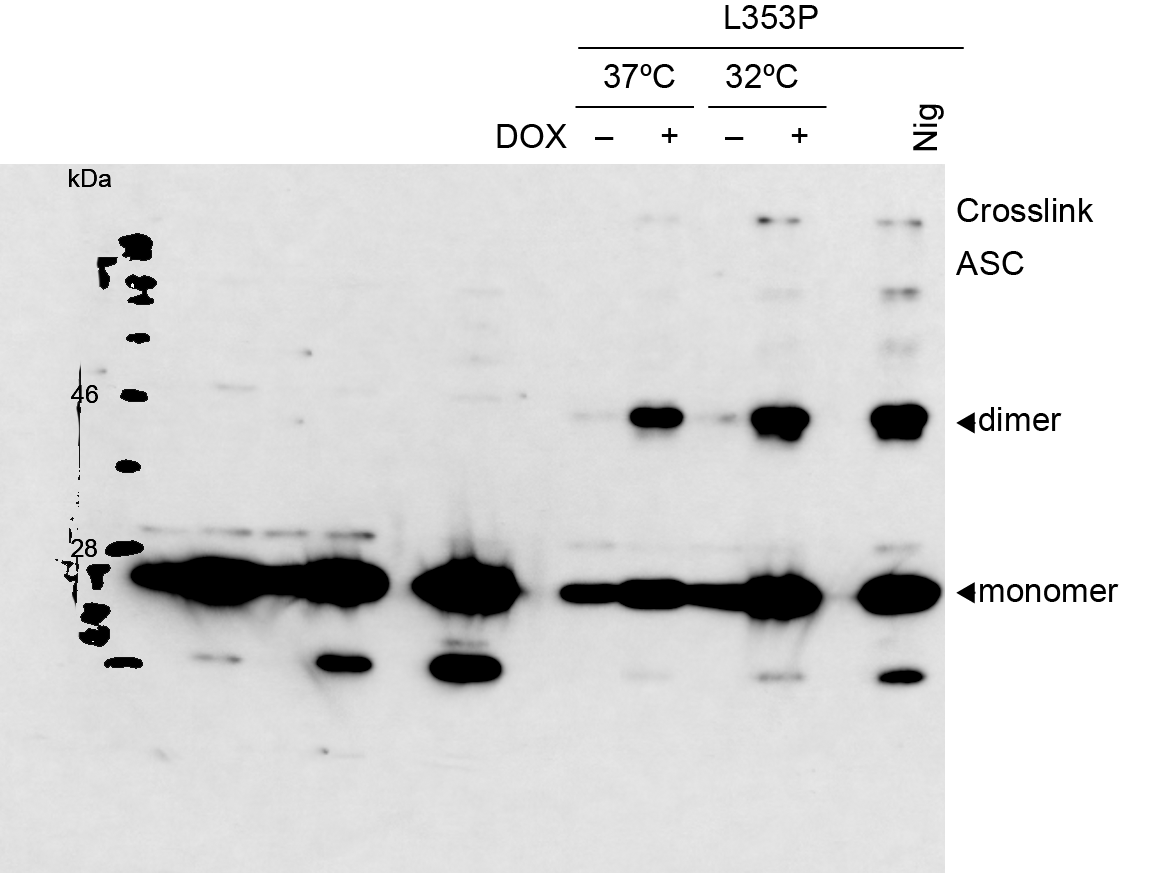

Supplement: Figure 3—source data 2. [file elife-75166-fig3-data2.zip › Figure_3-Source_data_2/Fig3E_ASC_crosslink_labeled.tif]

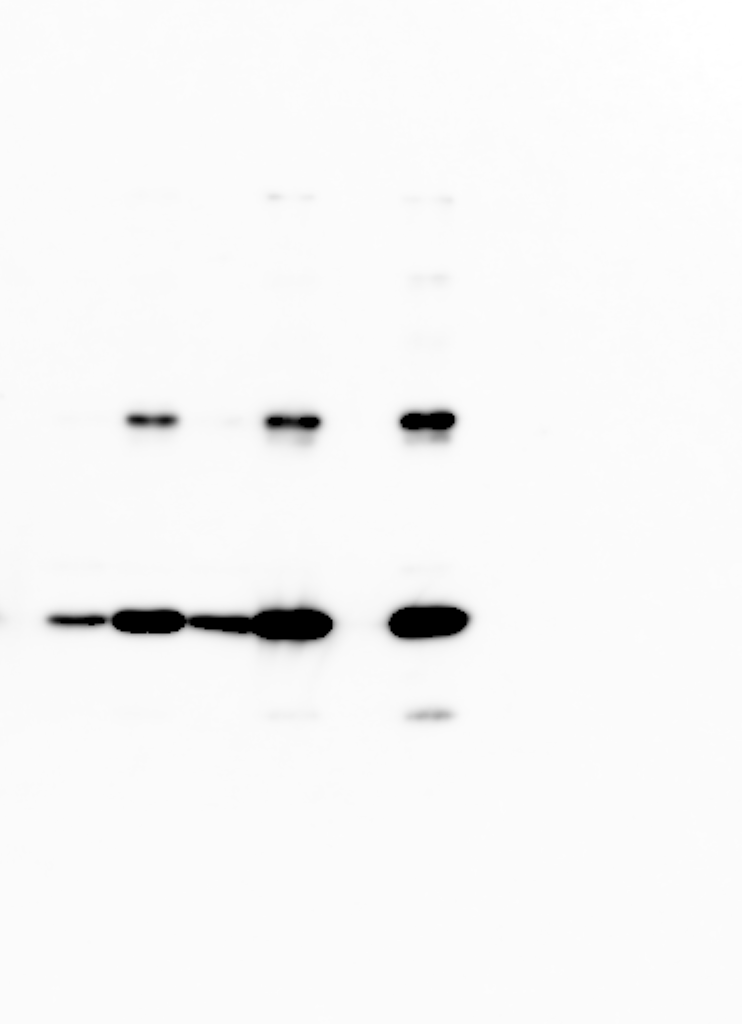

Supplement: Figure 3—source data 2. [file elife-75166-fig3-data2.zip › Figure_3-Source_data_2/Fig3E_ASC_crosslink_raw.tif]

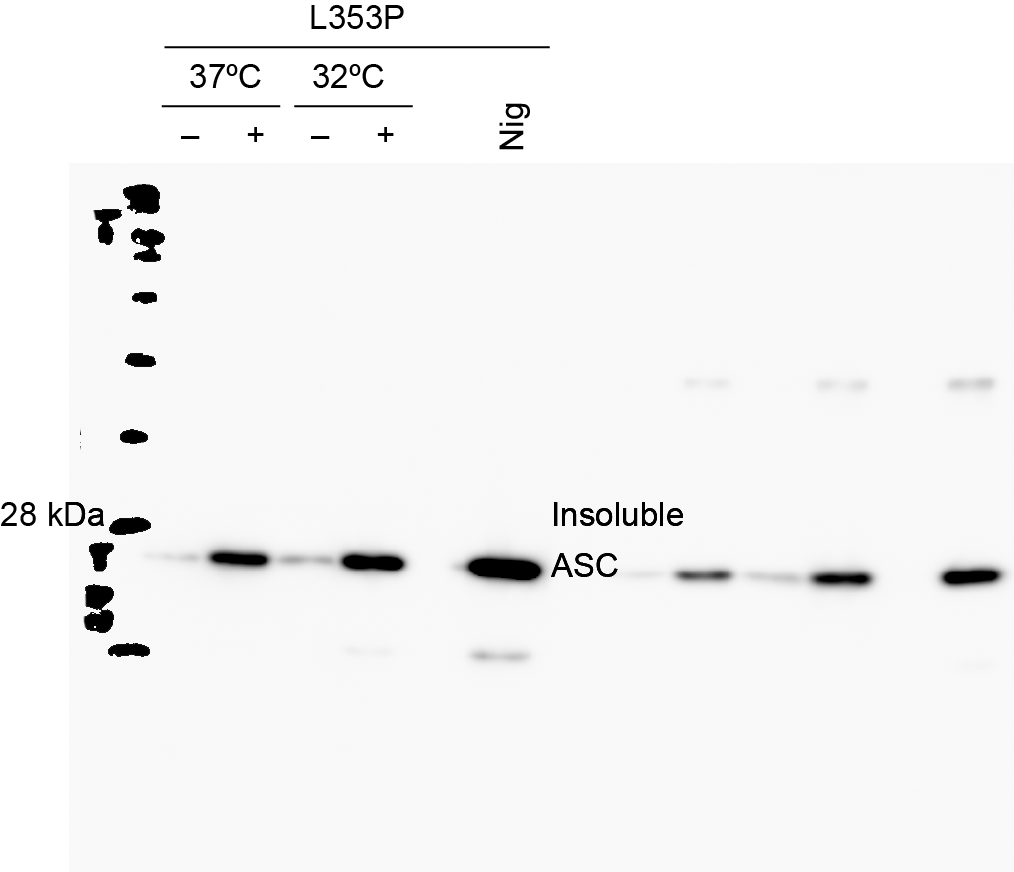

Supplement: Figure 3—source data 2. [file elife-75166-fig3-data2.zip › Figure_3-Source_data_2/Fig3E_ASC_insoluble_labeled.tif]

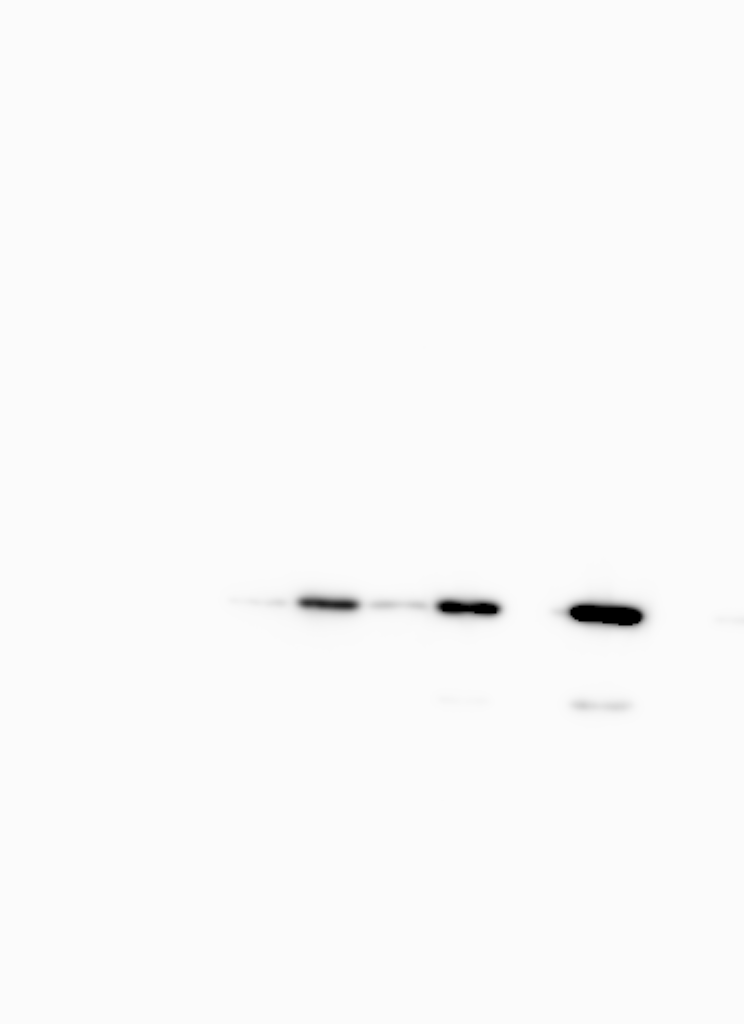

Supplement: Figure 3—source data 2. [file elife-75166-fig3-data2.zip › Figure_3-Source_data_2/Fig3E_ASC_insoluble_raw.tif]

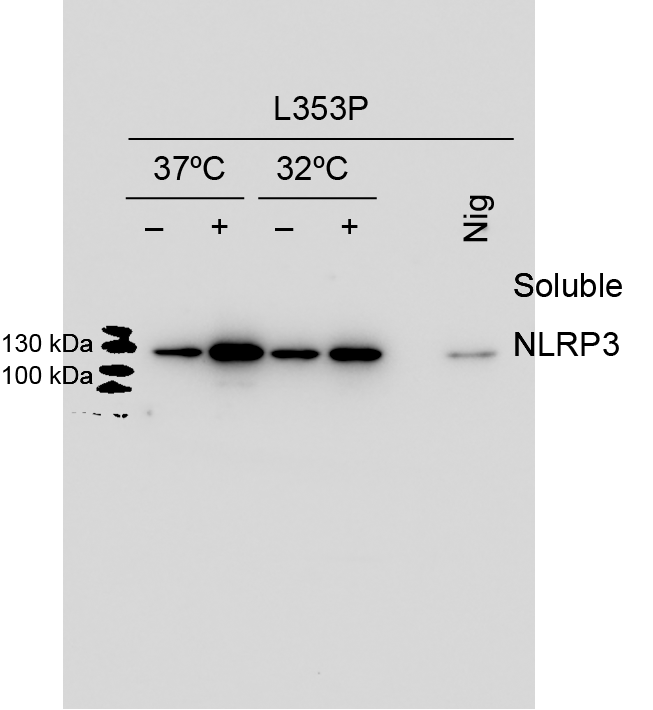

Supplement: Figure 3—source data 2. [file elife-75166-fig3-data2.zip › Figure_3-Source_data_2/Fig3E_NLRP3_labeled.tif]

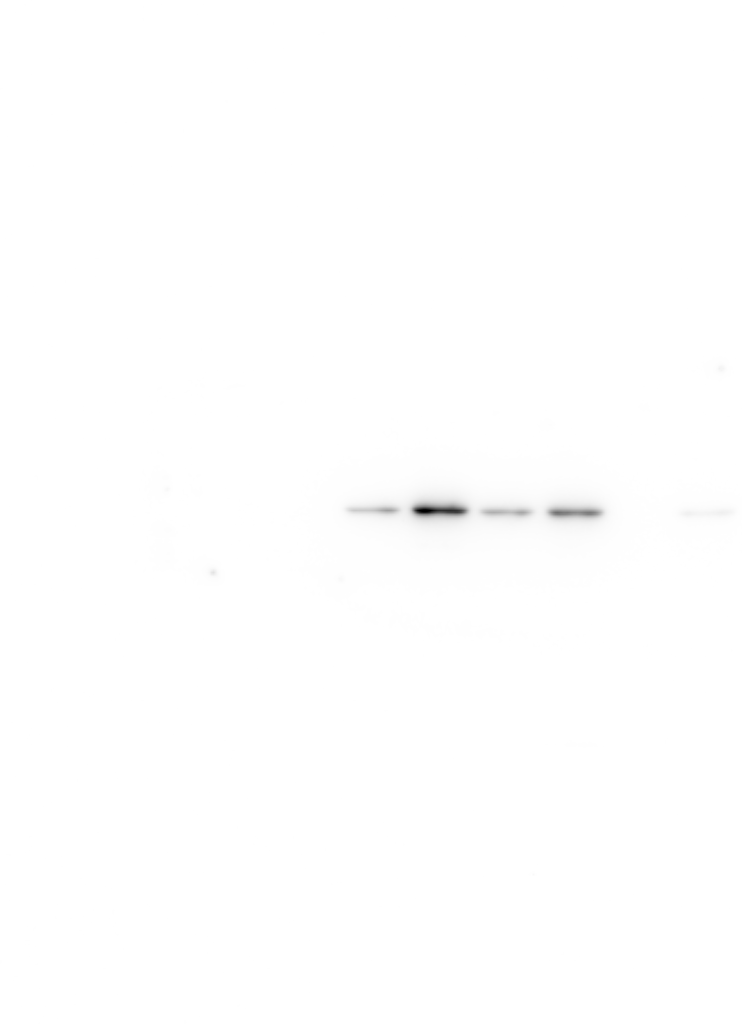

Supplement: Figure 3—source data 2. [file elife-75166-fig3-data2.zip › Figure_3-Source_data_2/Fig3E_NLRP3_raw.tif]

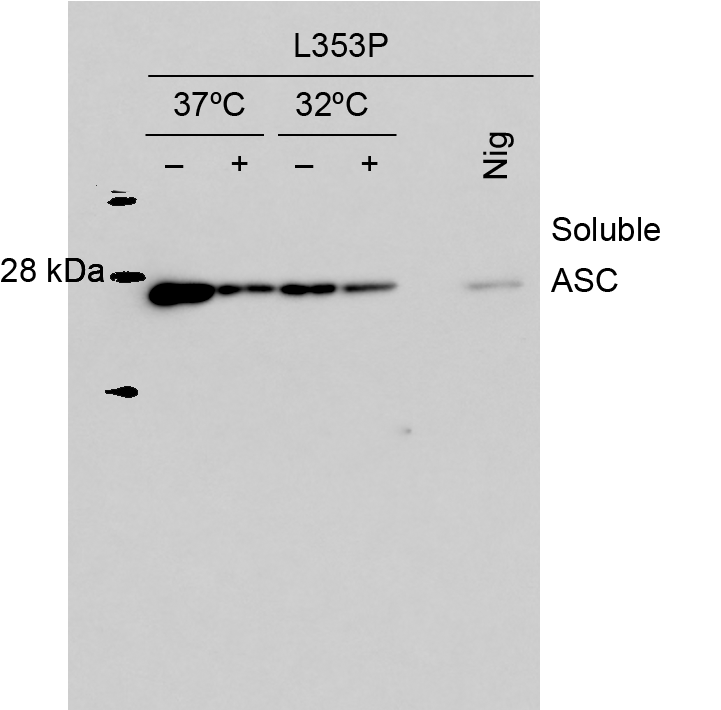

Supplement: Figure 3—source data 2. [file elife-75166-fig3-data2.zip › Figure_3-Source_data_2/Fig3EASC_soluble_labeled.tif]

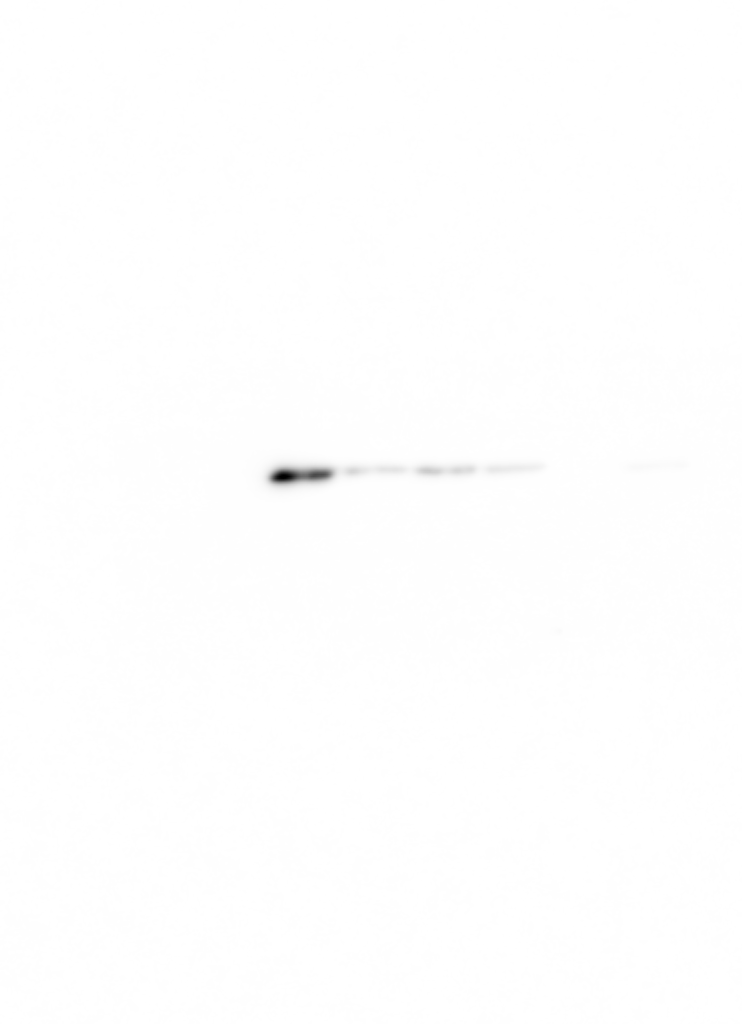

Supplement: Figure 3—source data 2. [file elife-75166-fig3-data2.zip › Figure_3-Source_data_2/Fig3EASC_soluble_raw.tif]

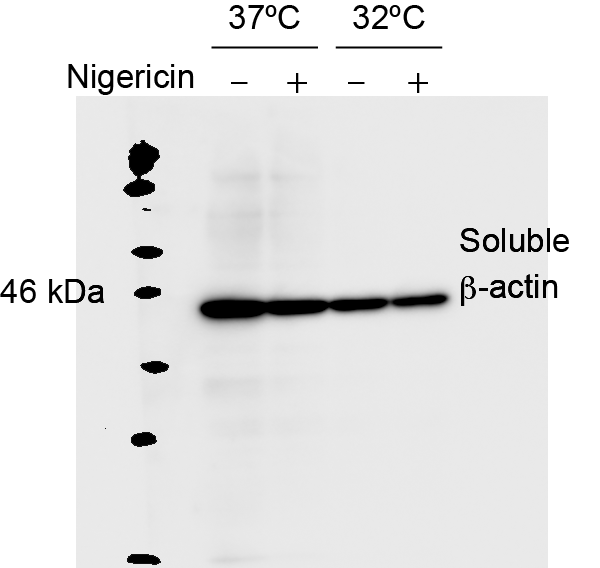

Supplement: Figure 3—source data 3. [file elife-75166-fig3-data3.zip › Figure_3-Source_data_3/Fig3F_actin_labeled.tif]

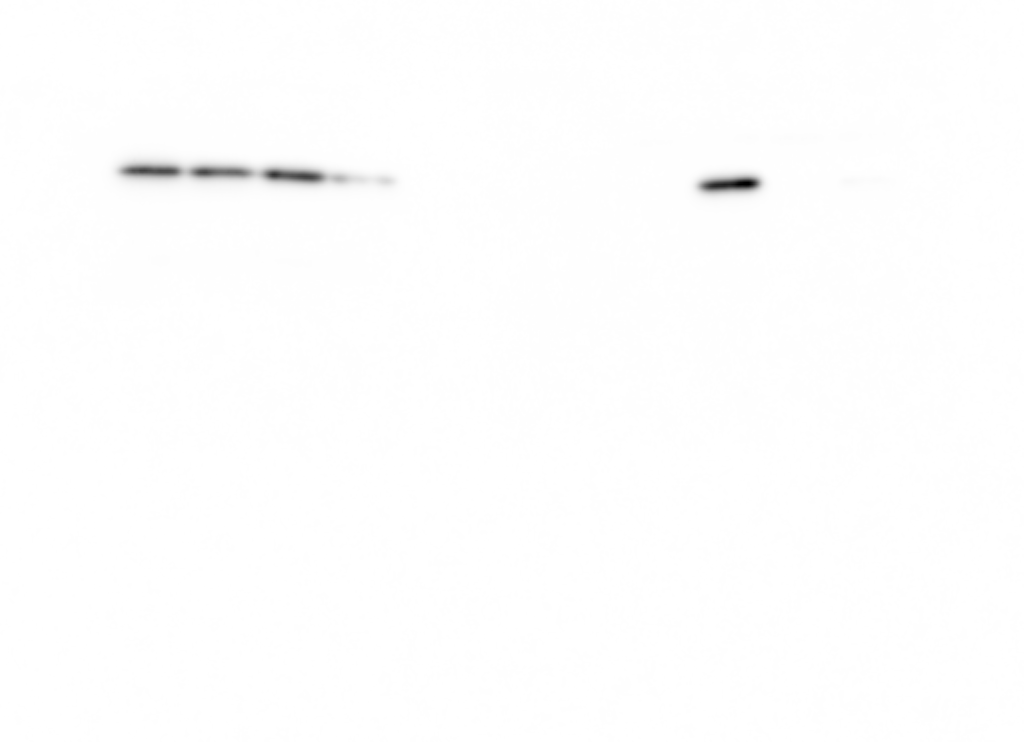

Supplement: Figure 3—source data 3. [file elife-75166-fig3-data3.zip › Figure_3-Source_data_3/Fig3F_ASC_ASC_soluble_insoluble_raw.tif]

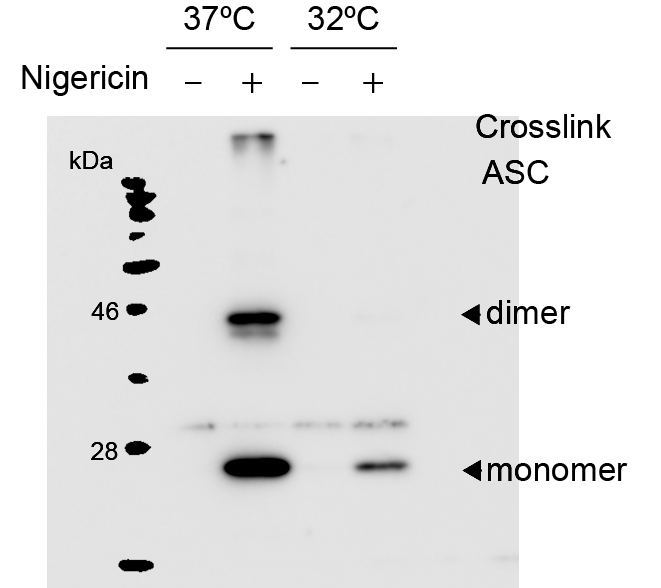

Supplement: Figure 3—source data 3. [file elife-75166-fig3-data3.zip › Figure_3-Source_data_3/Fig3F_ASC_crosslink_labeld.tif]

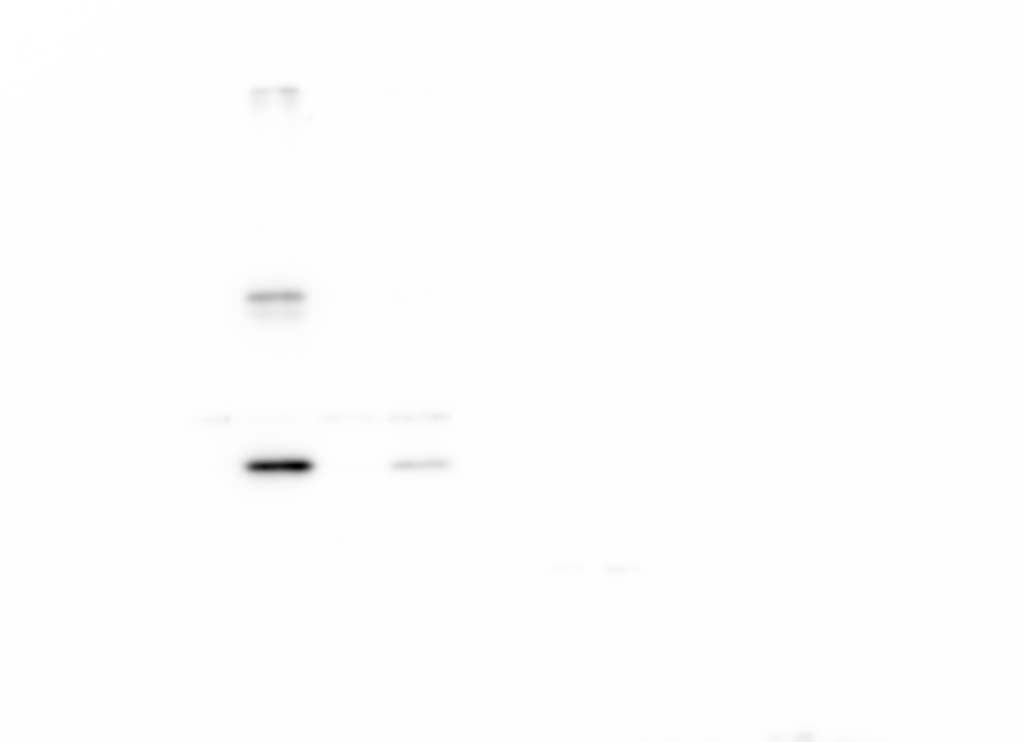

Supplement: Figure 3—source data 3. [file elife-75166-fig3-data3.zip › Figure_3-Source_data_3/Fig3F_ASC_crosslink_raw.tif]

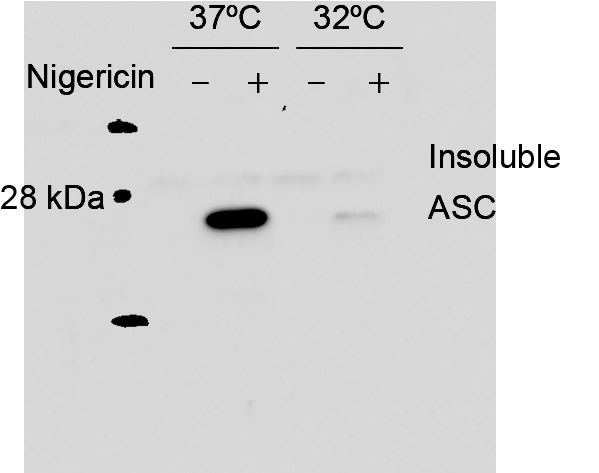

Supplement: Figure 3—source data 3. [file elife-75166-fig3-data3.zip › Figure_3-Source_data_3/Fig3F_ASC_insoluble_labeled.tif]

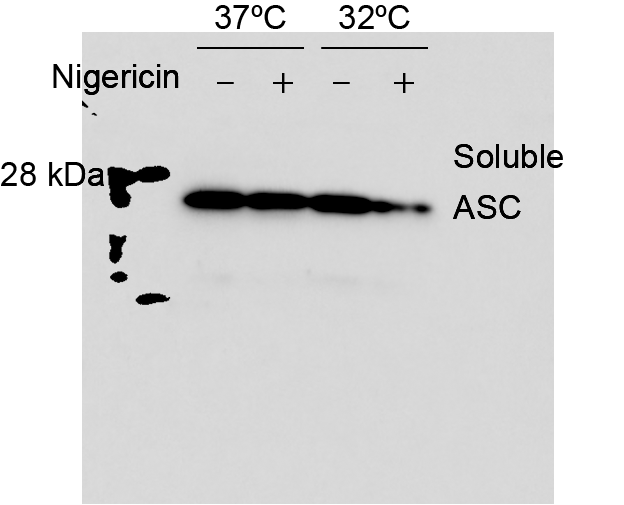

Supplement: Figure 3—source data 3. [file elife-75166-fig3-data3.zip › Figure_3-Source_data_3/Fig3F_ASC_soluble_labeled.tif]

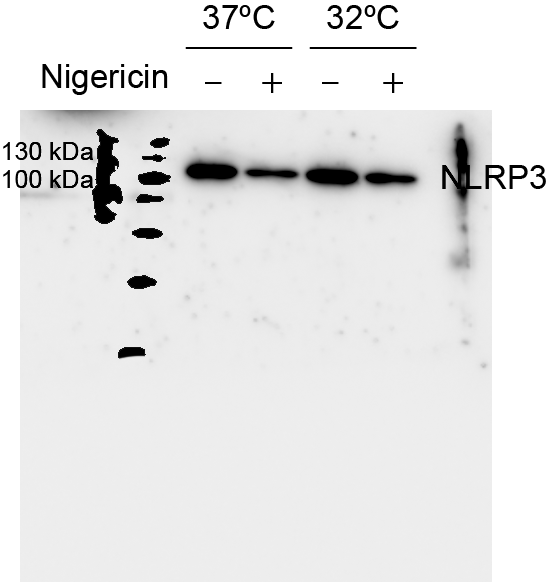

Supplement: Figure 3—source data 3. [file elife-75166-fig3-data3.zip › Figure_3-Source_data_3/Fig3F_NLRP3_labeled.tif]

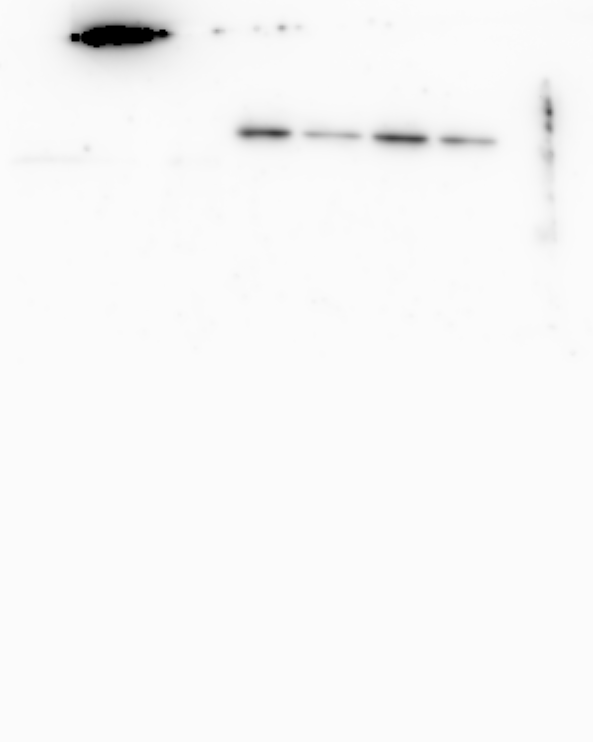

Supplement: Figure 3—source data 3. [file elife-75166-fig3-data3.zip › Figure_3-Source_data_3/Fig3F_NLRP3_raw.tif]

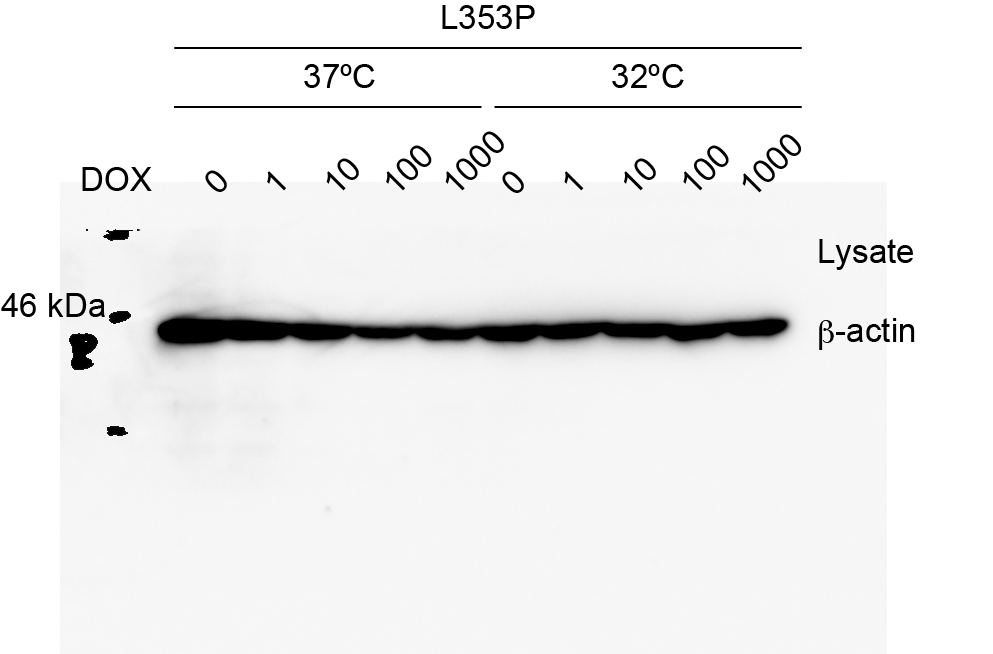

Supplement: Figure 3—figure supplement 1—source data 1. [file elife-75166-fig3-figsupp1-data1.zip › Figure_3-figure_supplement_1-Source_data_1/Fig3S1A_actin_labeled.tif]

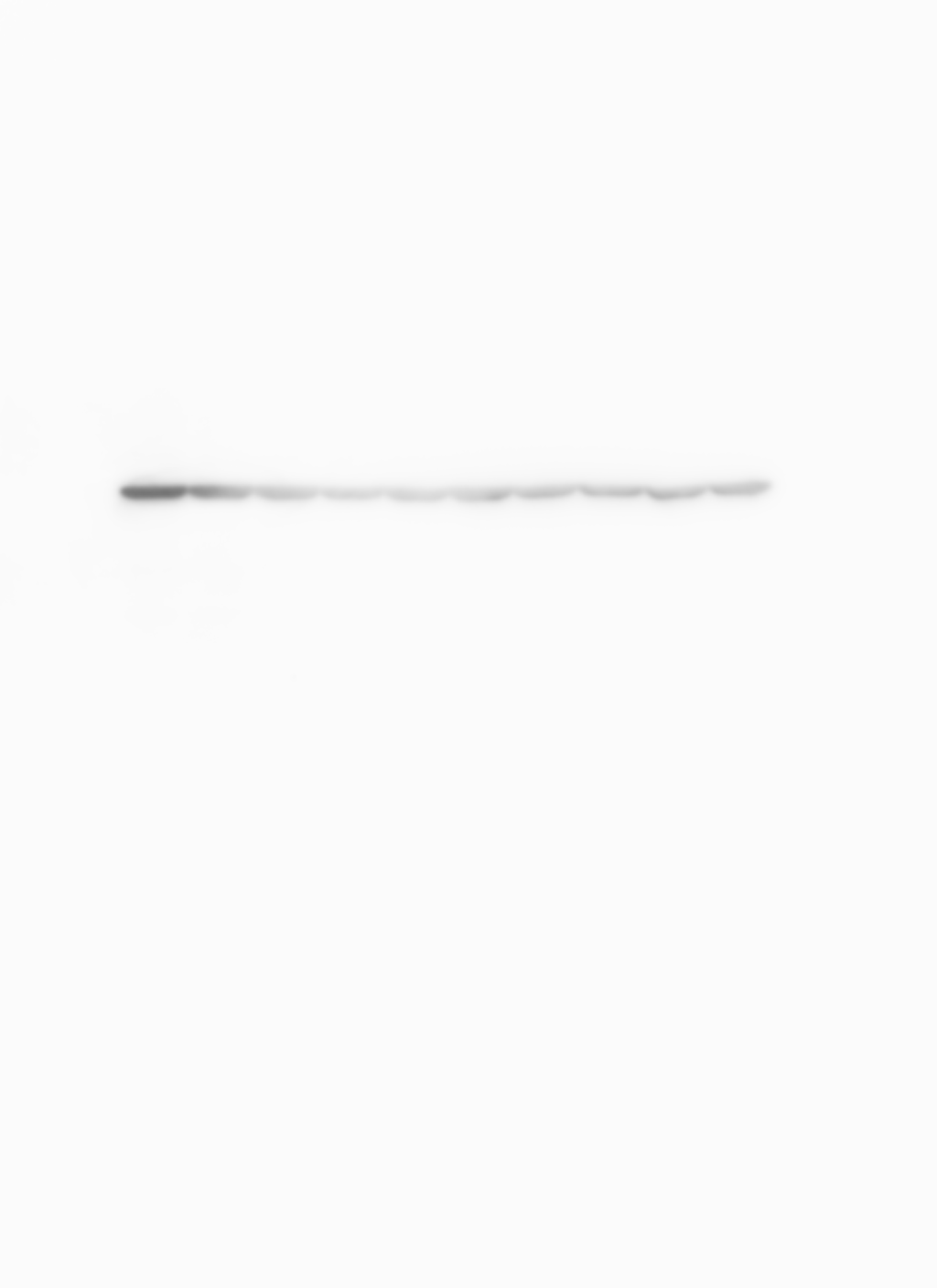

Supplement: Figure 3—figure supplement 1—source data 1. [file elife-75166-fig3-figsupp1-data1.zip › Figure_3-figure_supplement_1-Source_data_1/Fig3S1A_actin_raw.tif]

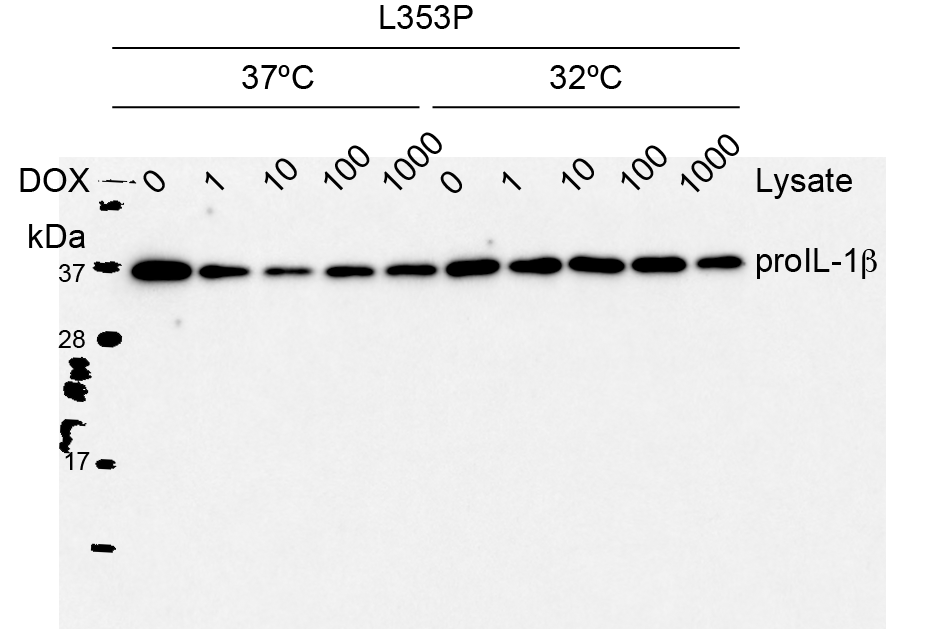

Supplement: Figure 3—figure supplement 1—source data 1. [file elife-75166-fig3-figsupp1-data1.zip › Figure_3-figure_supplement_1-Source_data_1/Fig3S1A_IL-1beta_lysate_labeled.tif]

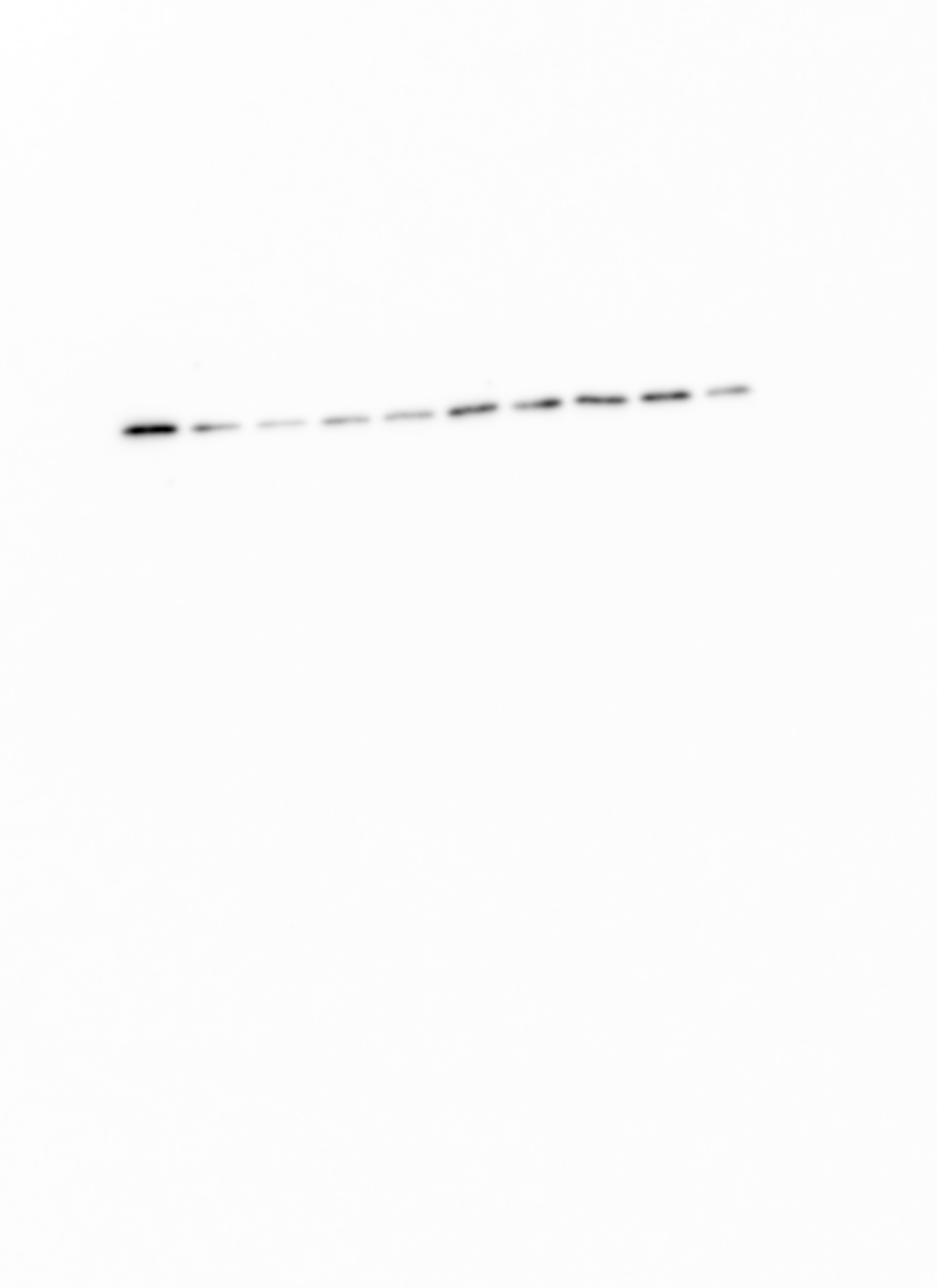

Supplement: Figure 3—figure supplement 1—source data 1. [file elife-75166-fig3-figsupp1-data1.zip › Figure_3-figure_supplement_1-Source_data_1/Fig3S1A_IL-1beta_lysate_raw.tif]

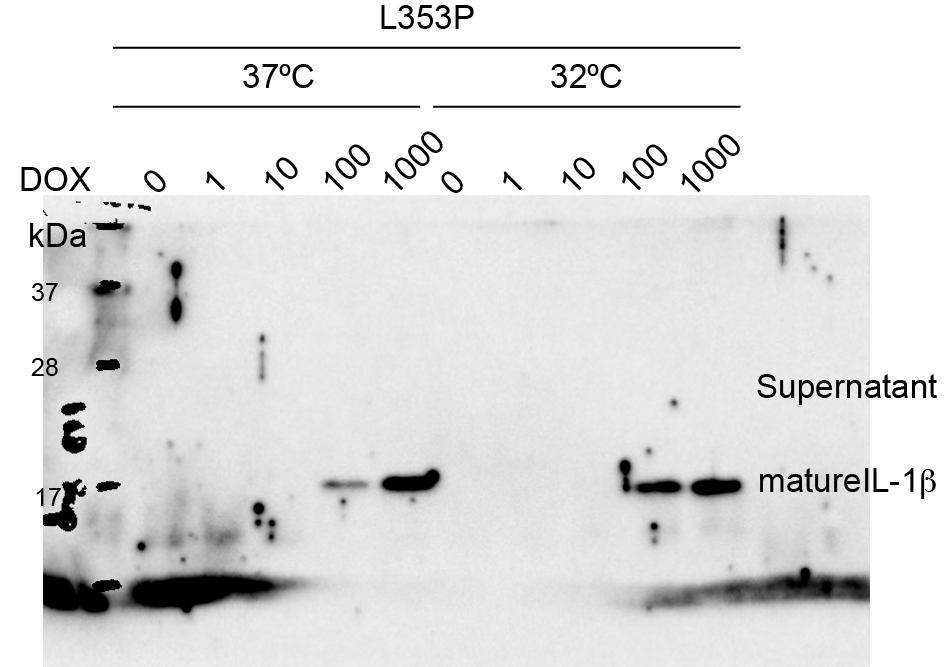

Supplement: Figure 3—figure supplement 1—source data 1. [file elife-75166-fig3-figsupp1-data1.zip › Figure_3-figure_supplement_1-Source_data_1/Fig3S1A_IL-1beta_sup_labeled.tif]

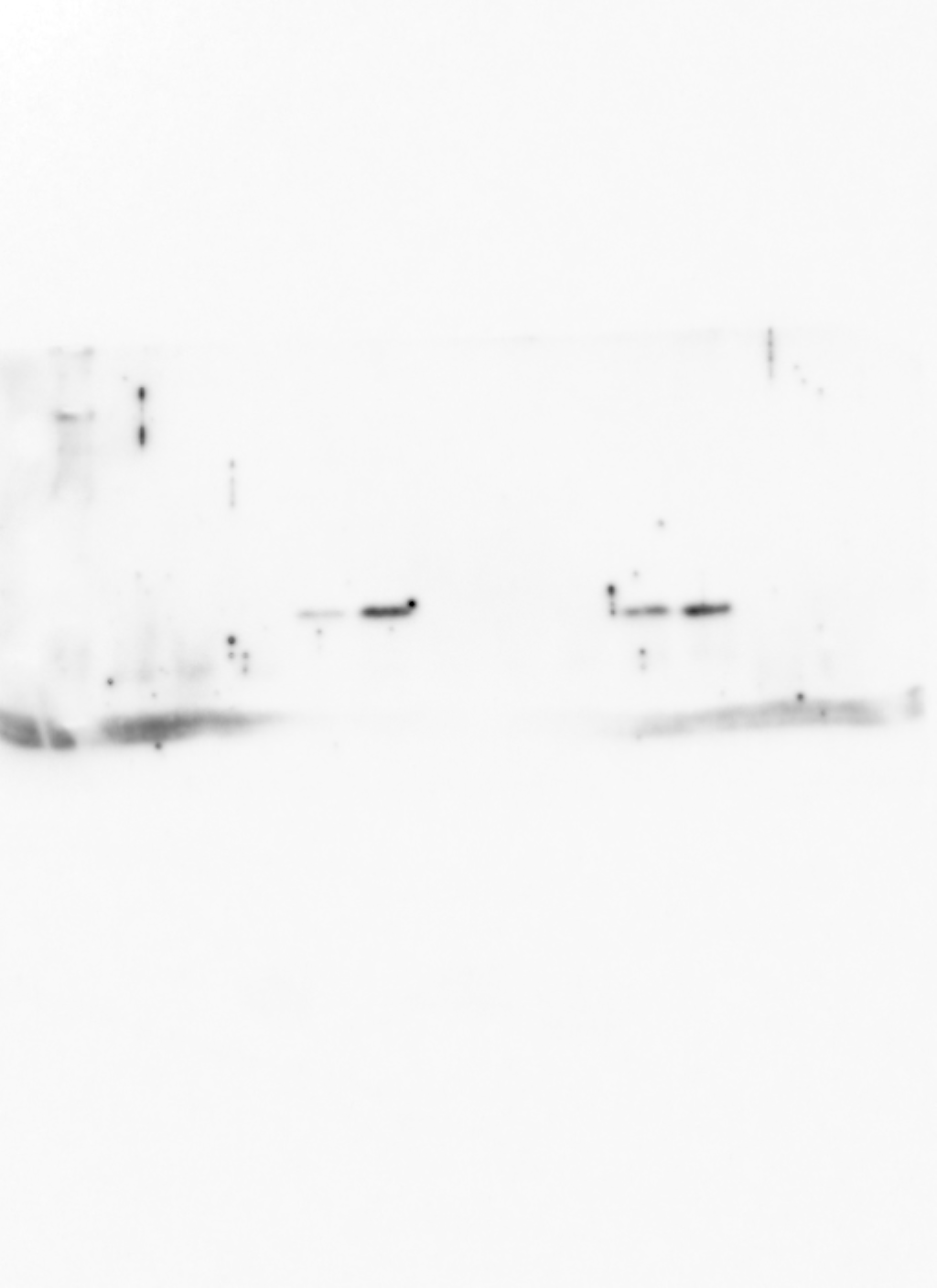

Supplement: Figure 3—figure supplement 1—source data 1. [file elife-75166-fig3-figsupp1-data1.zip › Figure_3-figure_supplement_1-Source_data_1/Fig3S1A_IL-1beta_sup_raw.tif]

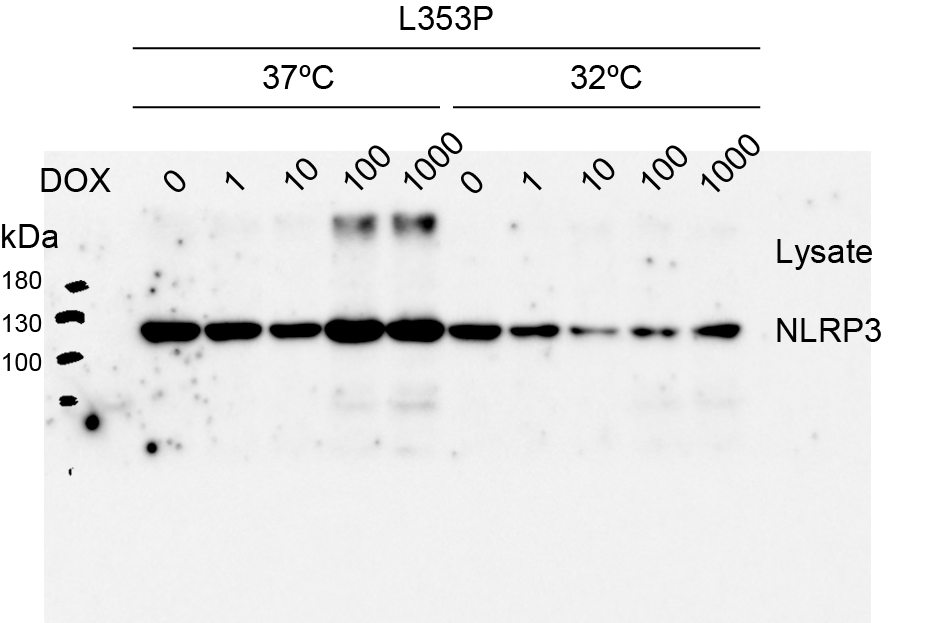

Supplement: Figure 3—figure supplement 1—source data 1. [file elife-75166-fig3-figsupp1-data1.zip › Figure_3-figure_supplement_1-Source_data_1/Fig3S1A_NLRP3_lysate_labeled.tif]

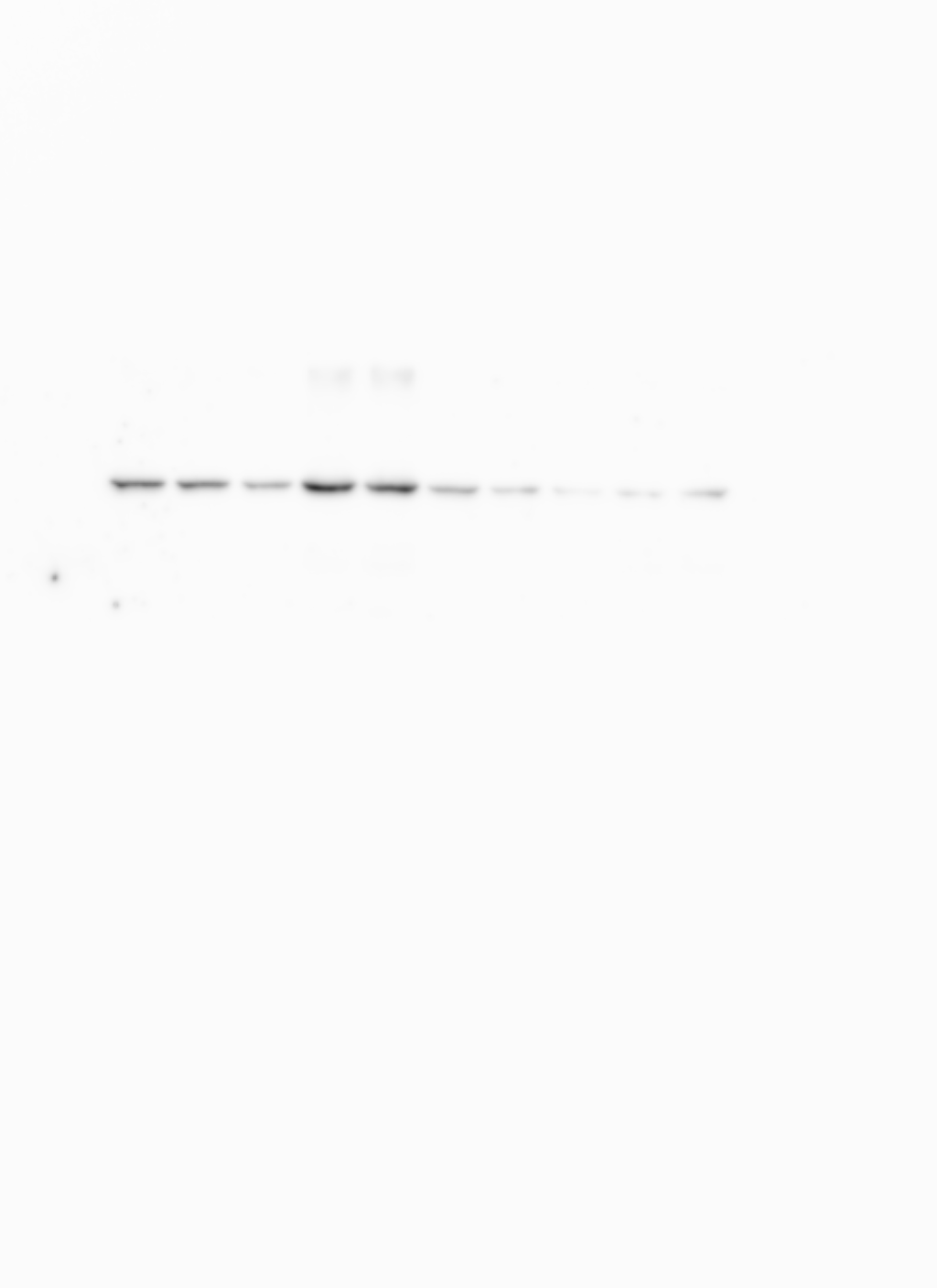

Supplement: Figure 3—figure supplement 1—source data 1. [file elife-75166-fig3-figsupp1-data1.zip › Figure_3-figure_supplement_1-Source_data_1/Fig3S1A_NLRP3_lysate_raw.tif]

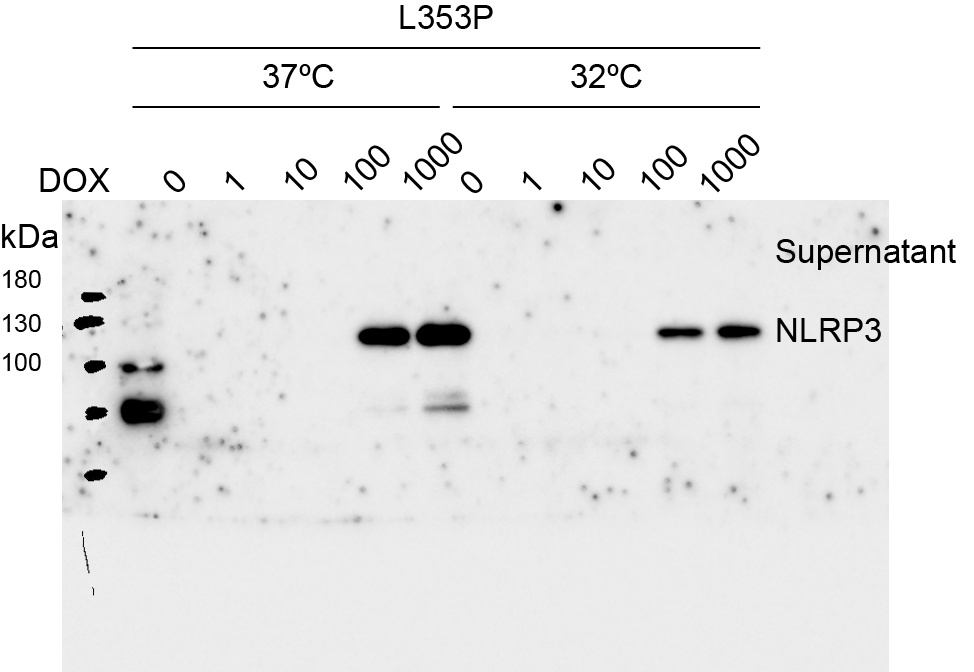

Supplement: Figure 3—figure supplement 1—source data 1. [file elife-75166-fig3-figsupp1-data1.zip › Figure_3-figure_supplement_1-Source_data_1/Fig3S1A_NLRP3_Sup_Labeled.tif]

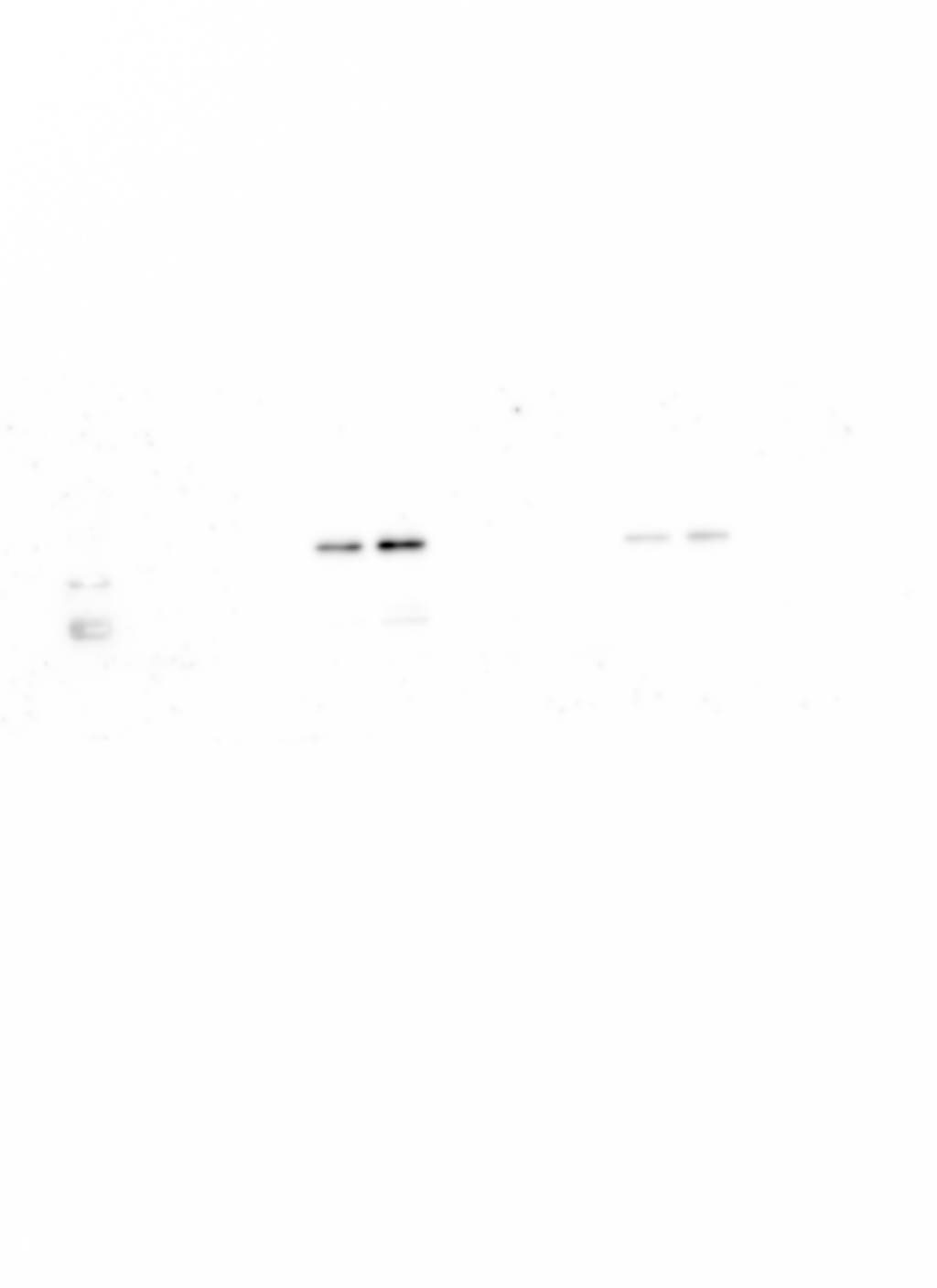

Supplement: Figure 3—figure supplement 1—source data 1. [file elife-75166-fig3-figsupp1-data1.zip › Figure_3-figure_supplement_1-Source_data_1/Fig3S1A_NLRP3_Sup_raw.tif]

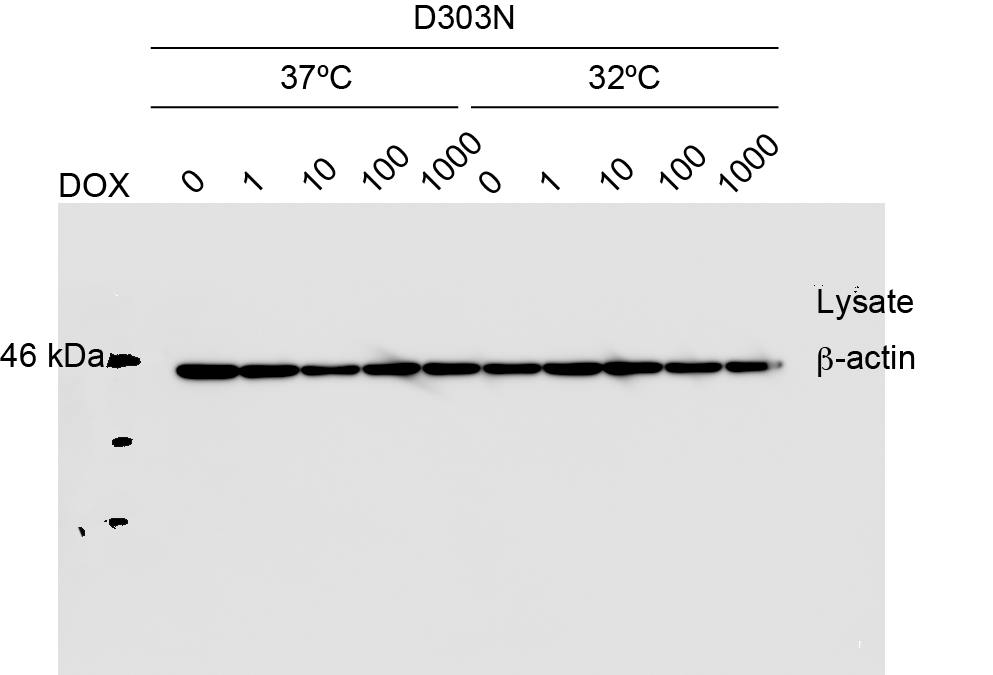

Supplement: Figure 3—figure supplement 1—source data 2. [file elife-75166-fig3-figsupp1-data2.zip › Figure_3-figure_supplement_1-Source_data_2/Fig3S1B_actin_labeled.tif]

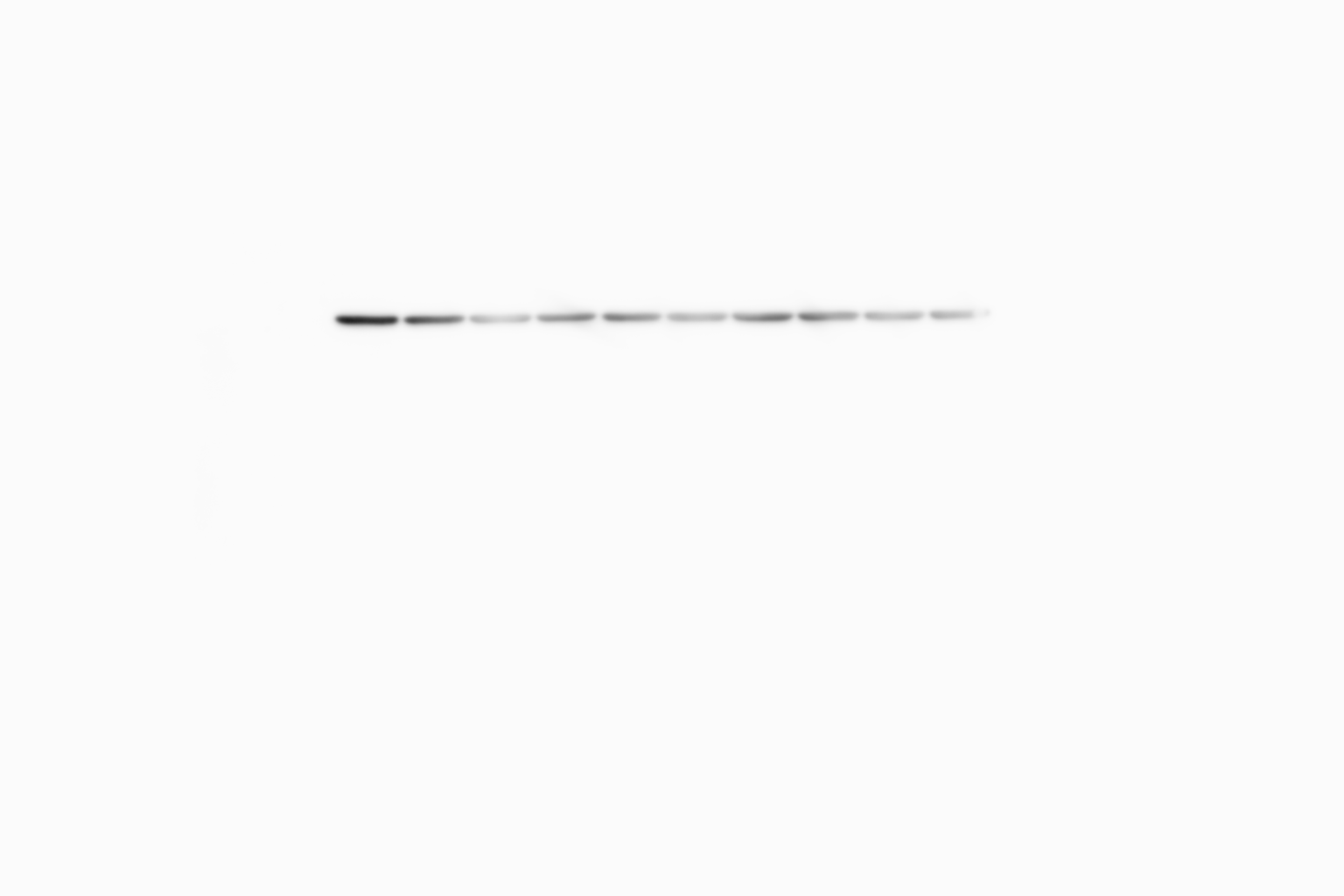

Supplement: Figure 3—figure supplement 1—source data 2. [file elife-75166-fig3-figsupp1-data2.zip › Figure_3-figure_supplement_1-Source_data_2/Fig3S1B_actin_raw.tif]

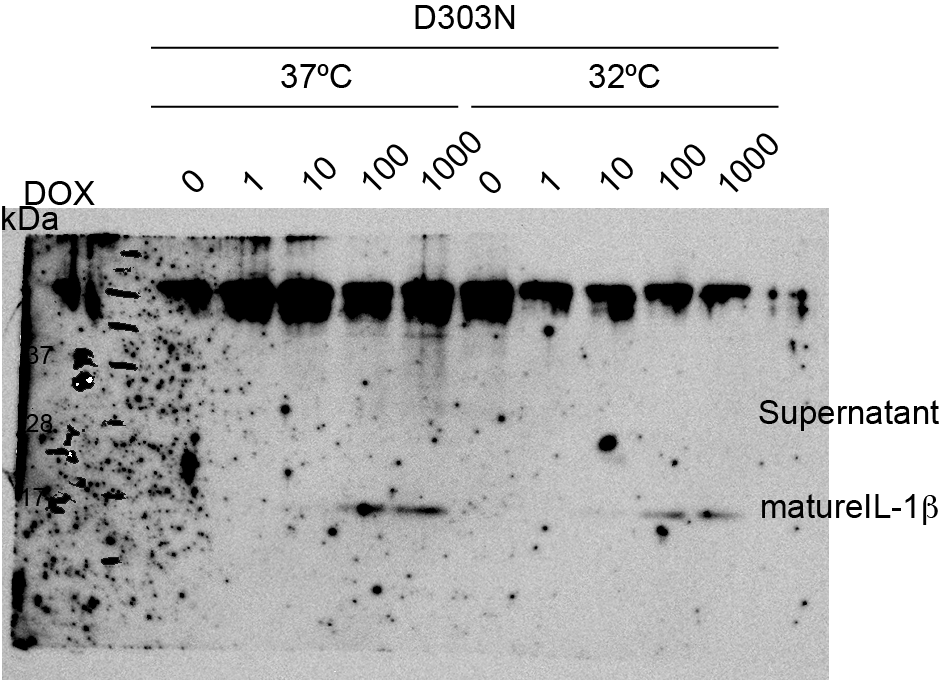

Supplement: Figure 3—figure supplement 1—source data 2. [file elife-75166-fig3-figsupp1-data2.zip › Figure_3-figure_supplement_1-Source_data_2/Fig3S1B_IL-1beta_sup_labeled.tif]

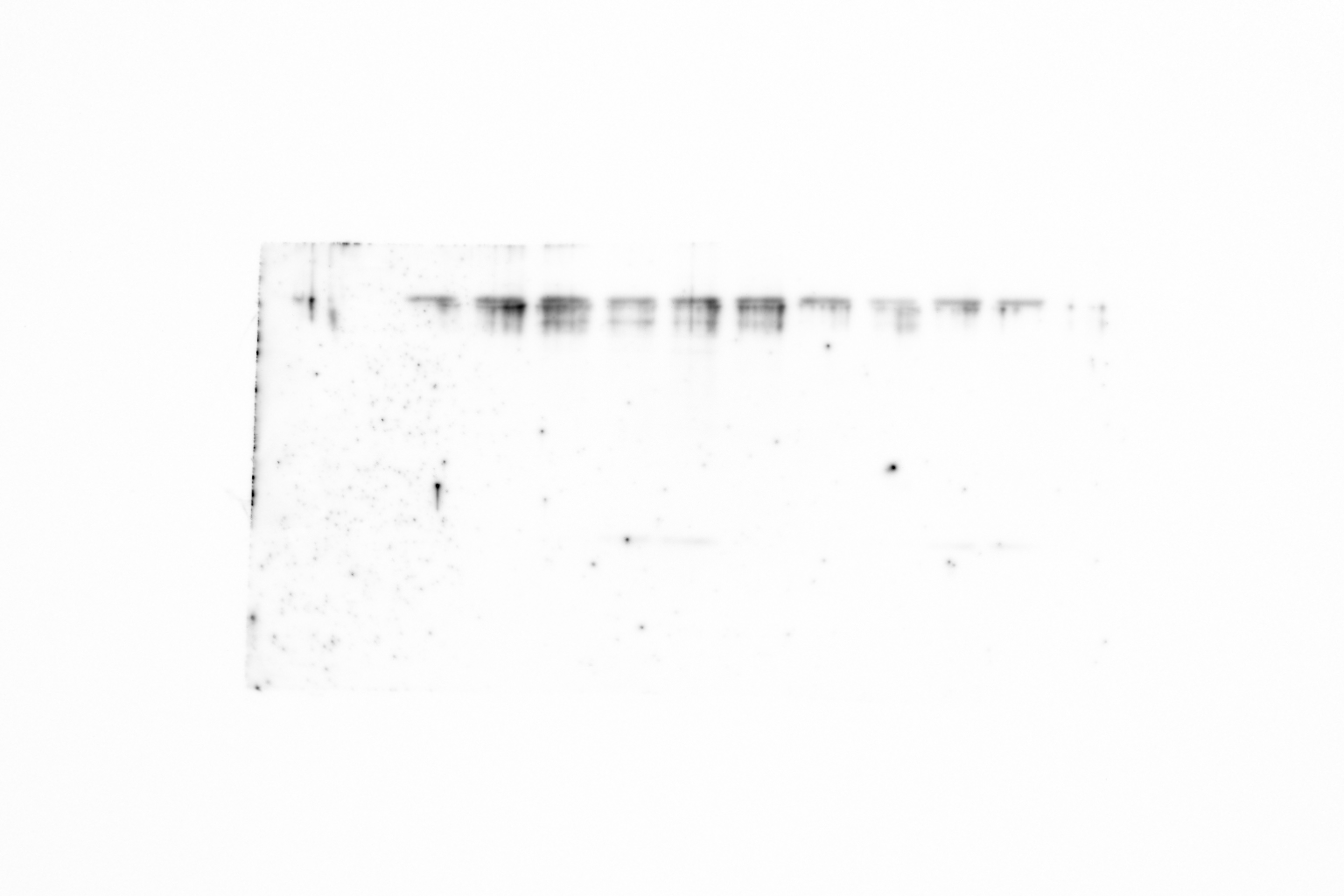

Supplement: Figure 3—figure supplement 1—source data 2. [file elife-75166-fig3-figsupp1-data2.zip › Figure_3-figure_supplement_1-Source_data_2/Fig3S1B_IL-1beta_sup_raw.tif]

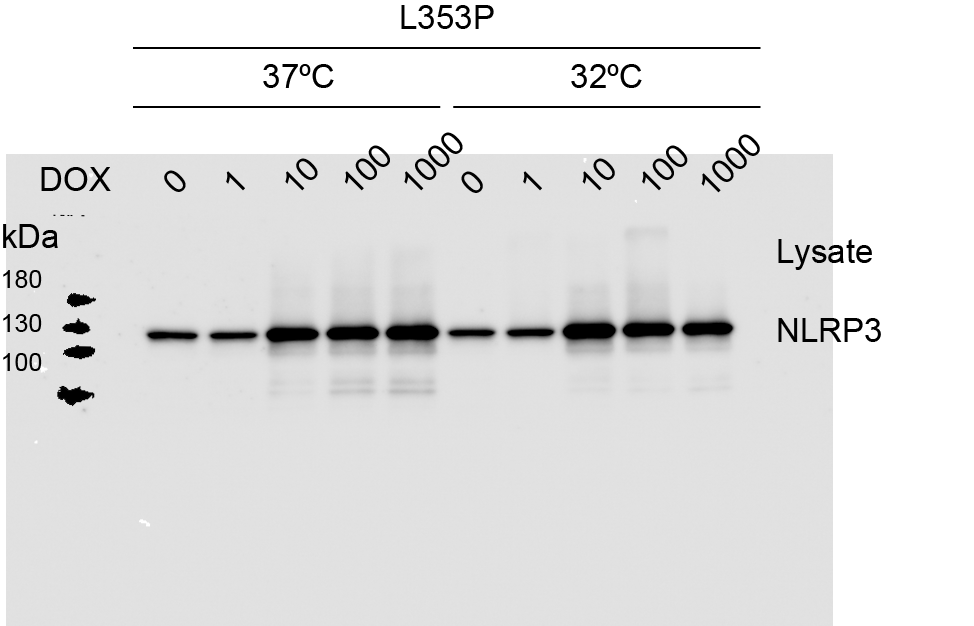

Supplement: Figure 3—figure supplement 1—source data 2. [file elife-75166-fig3-figsupp1-data2.zip › Figure_3-figure_supplement_1-Source_data_2/Fig3S1B_NLRP3_Lysate_labeled.tif]

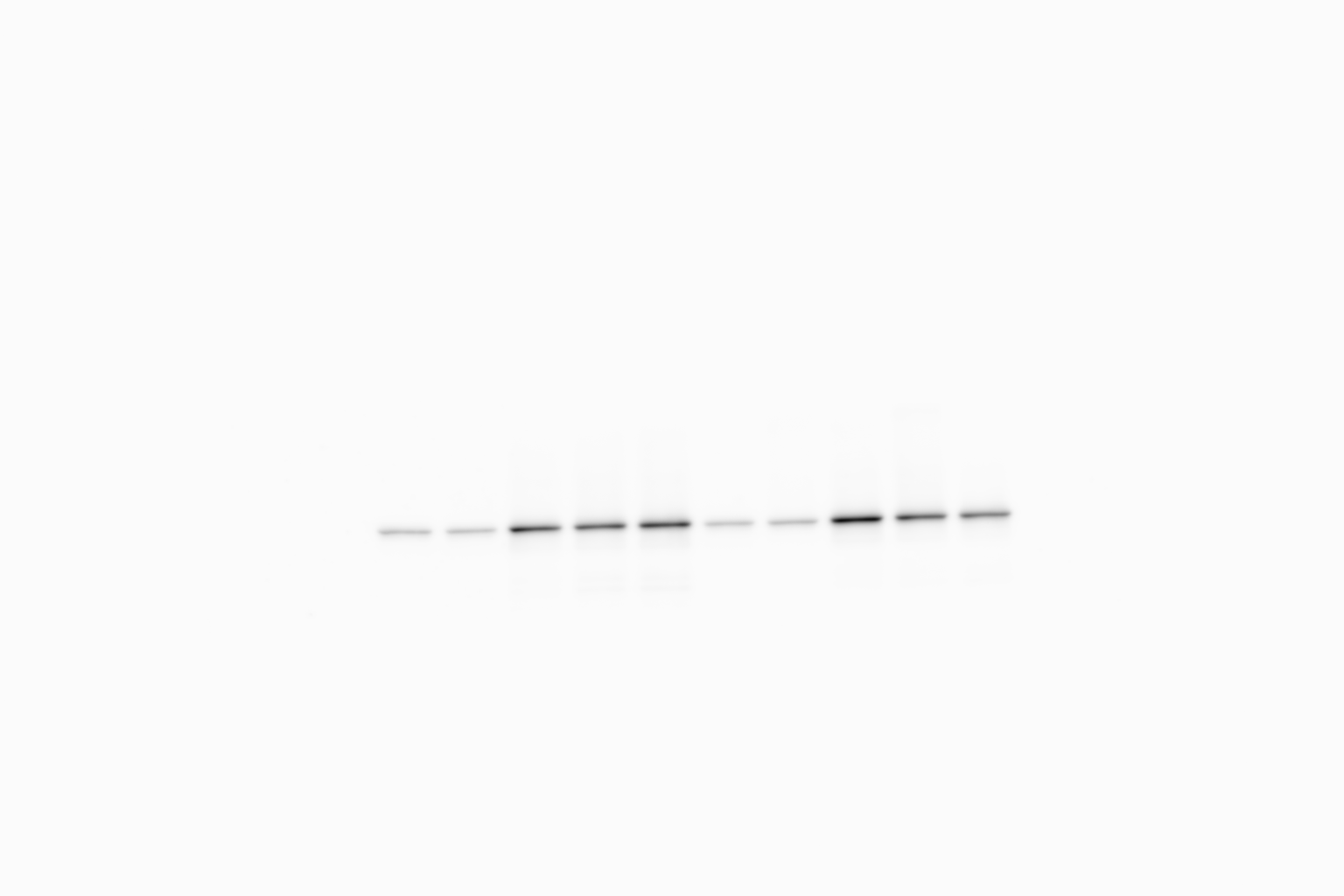

Supplement: Figure 3—figure supplement 1—source data 2. [file elife-75166-fig3-figsupp1-data2.zip › Figure_3-figure_supplement_1-Source_data_2/Fig3S1B_NLRP3_Lysate_raw.tif]

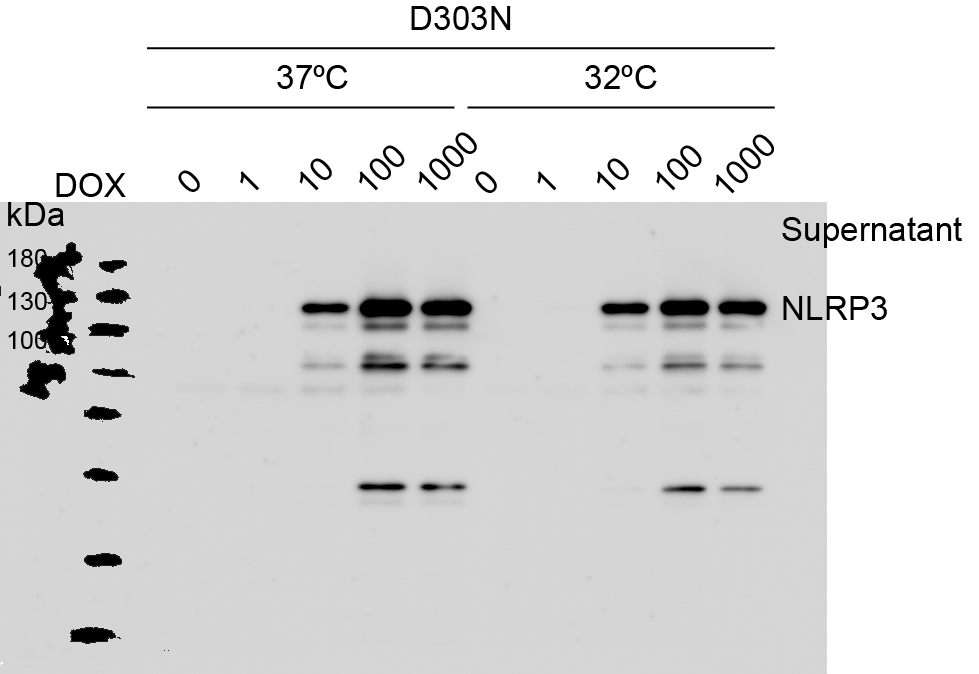

Supplement: Figure 3—figure supplement 1—source data 2. [file elife-75166-fig3-figsupp1-data2.zip › Figure_3-figure_supplement_1-Source_data_2/Fig3S1B_NLRP3_sup_labeled.tif]

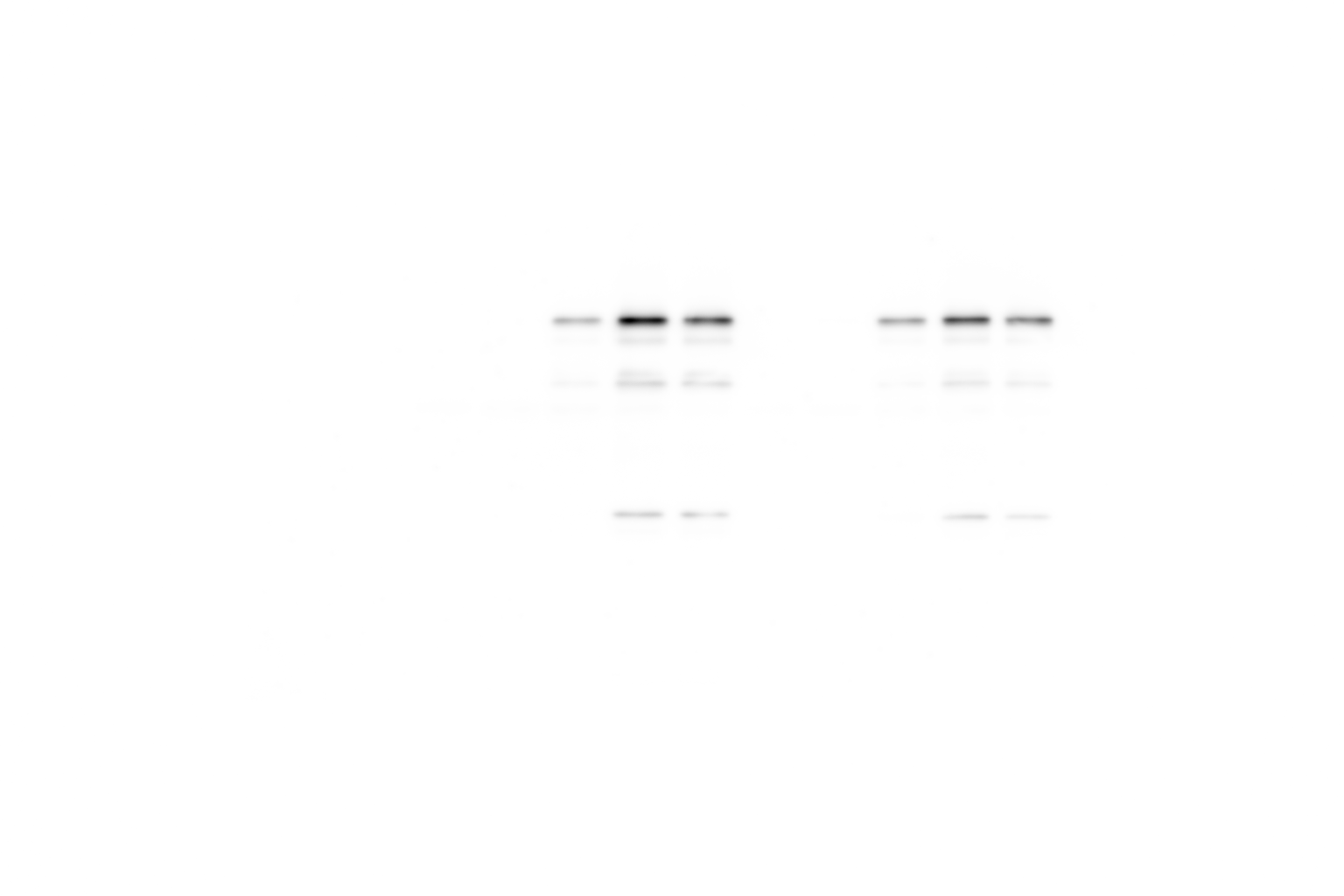

Supplement: Figure 3—figure supplement 1—source data 2. [file elife-75166-fig3-figsupp1-data2.zip › Figure_3-figure_supplement_1-Source_data_2/Fig3S1B_NLRP3_sup_raw.tif]

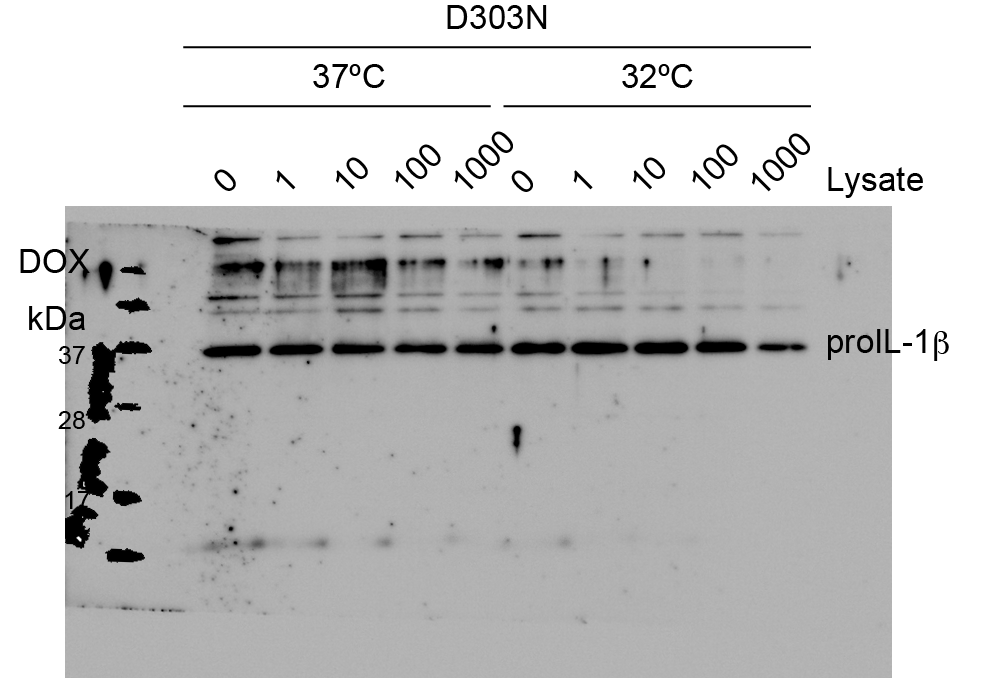

Supplement: Figure 3—figure supplement 1—source data 2. [file elife-75166-fig3-figsupp1-data2.zip › Figure_3-figure_supplement_1-Source_data_2/FigS3B_IL-1beta_lysate_labeled.tif]

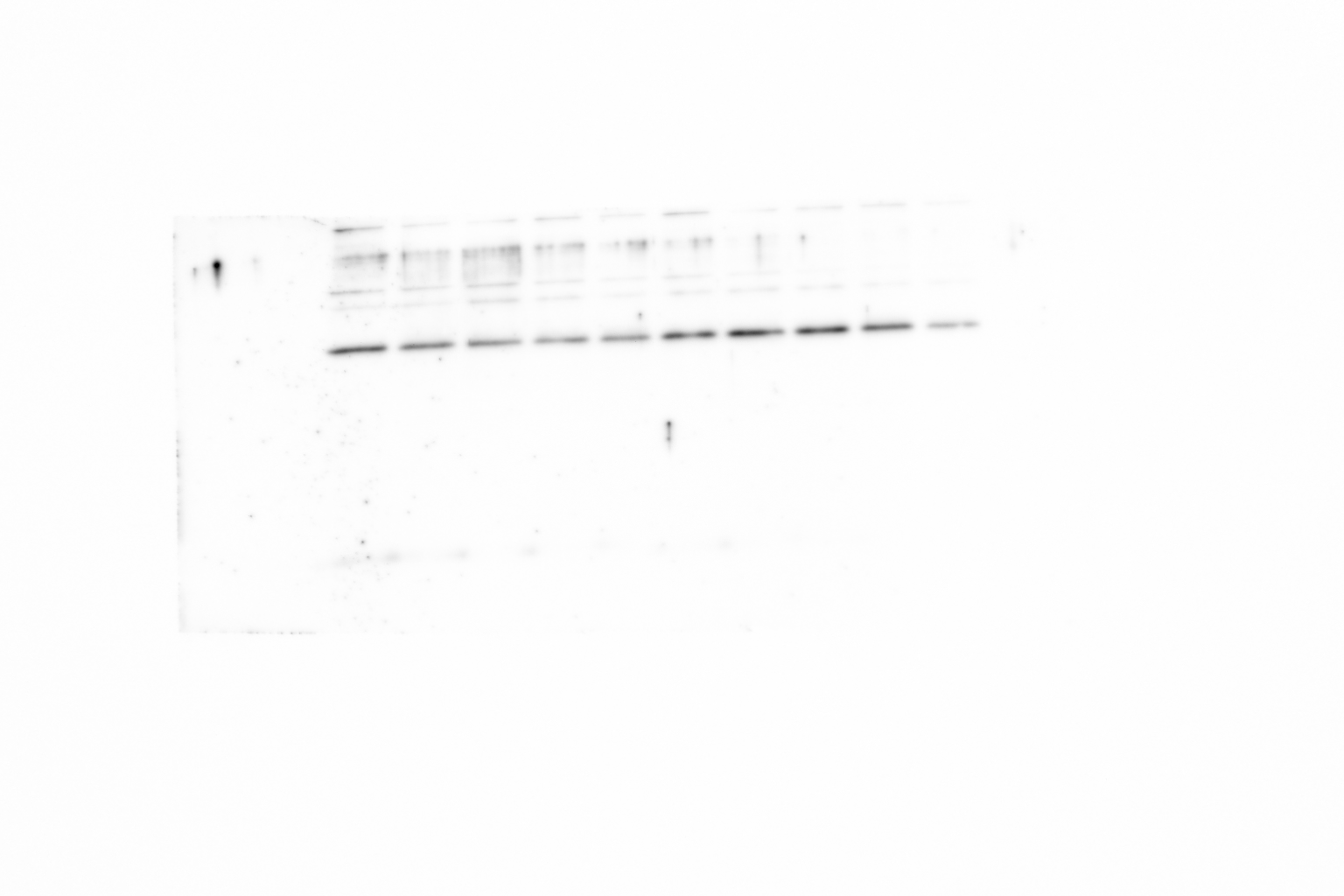

Supplement: Figure 3—figure supplement 1—source data 2. [file elife-75166-fig3-figsupp1-data2.zip › Figure_3-figure_supplement_1-Source_data_2/FigS3B_IL-1beta_lysate_raw.tif]

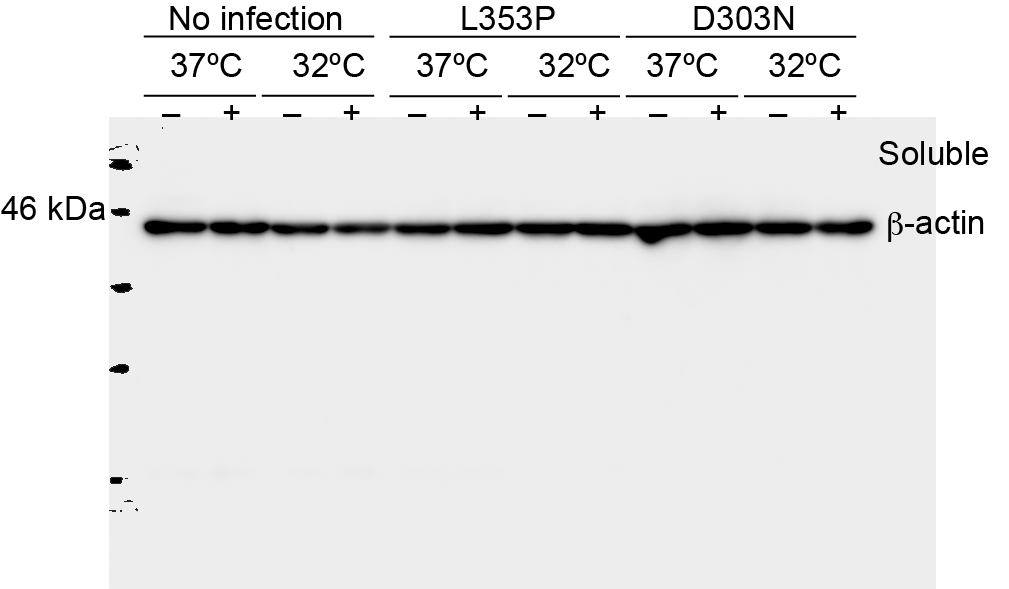

Supplement: Figure 3—figure supplement 1—source data 3. [file elife-75166-fig3-figsupp1-data3.zip › Figure_3-figure_supplement_1-Source_data_3/Fig3S1C_actin_labeled.tif]

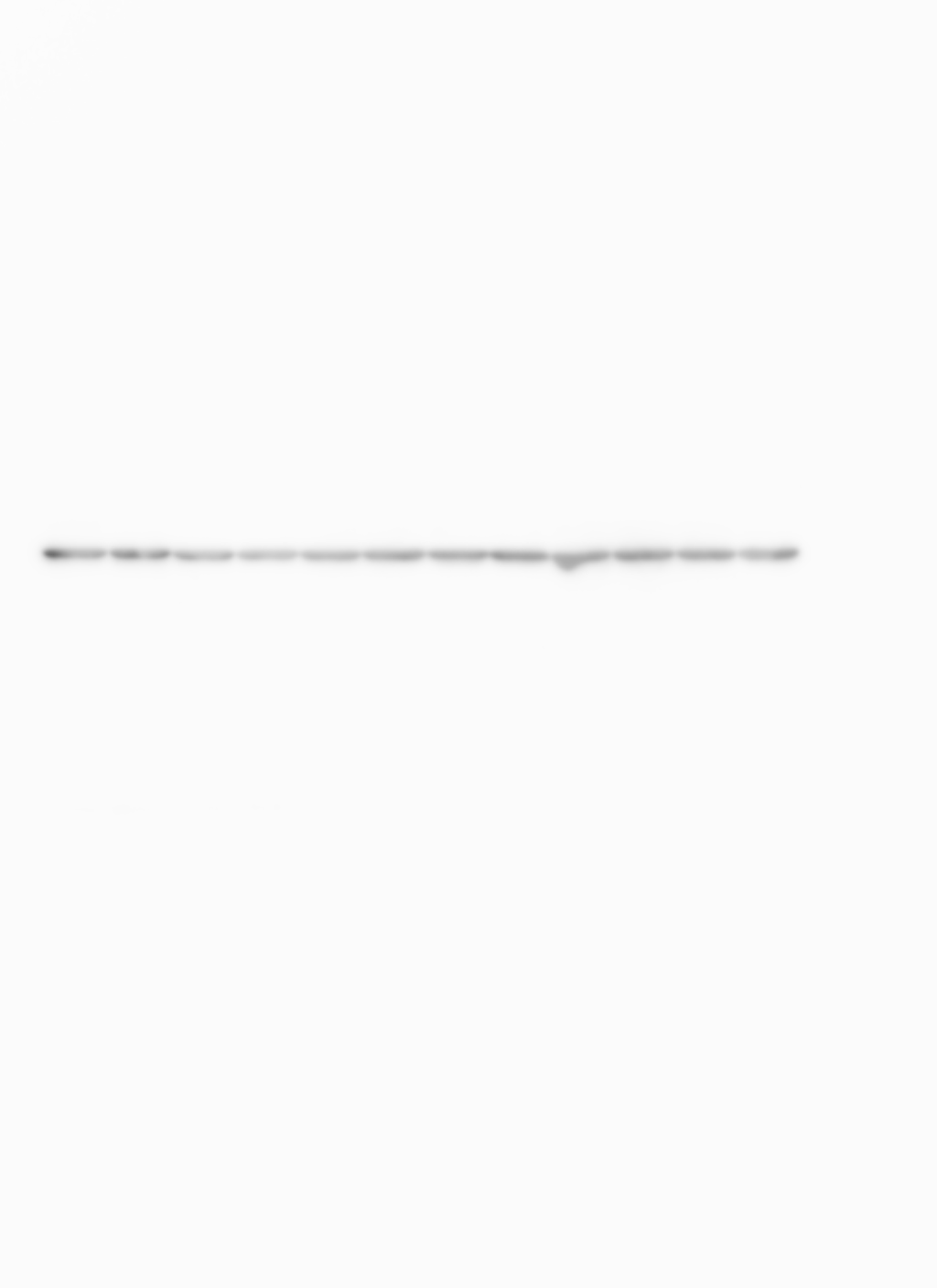

Supplement: Figure 3—figure supplement 1—source data 3. [file elife-75166-fig3-figsupp1-data3.zip › Figure_3-figure_supplement_1-Source_data_3/Fig3S1C_actin_raw.tif]

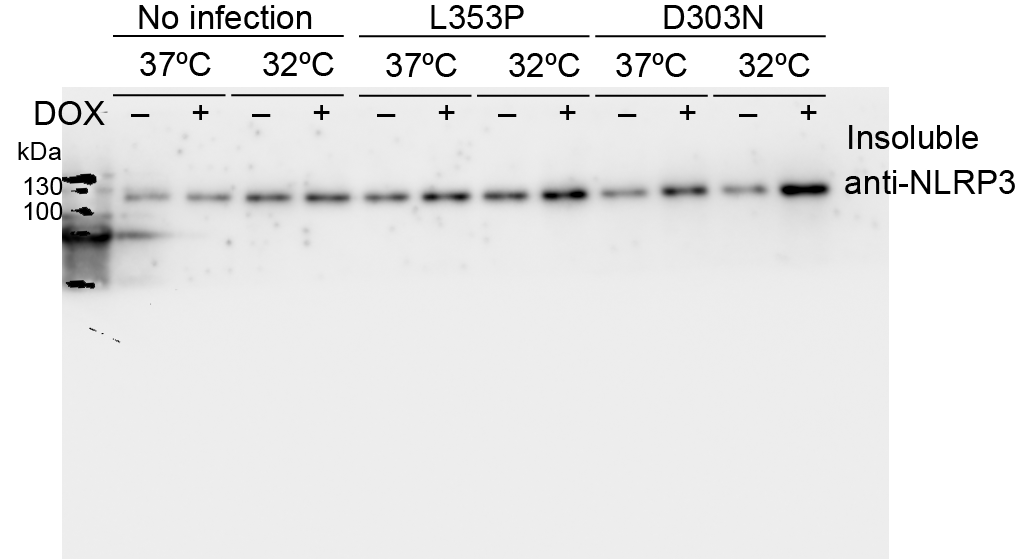

Supplement: Figure 3—figure supplement 1—source data 3. [file elife-75166-fig3-figsupp1-data3.zip › Figure_3-figure_supplement_1-Source_data_3/FigS1C_NLRP3_insoluble_labeled.tif]

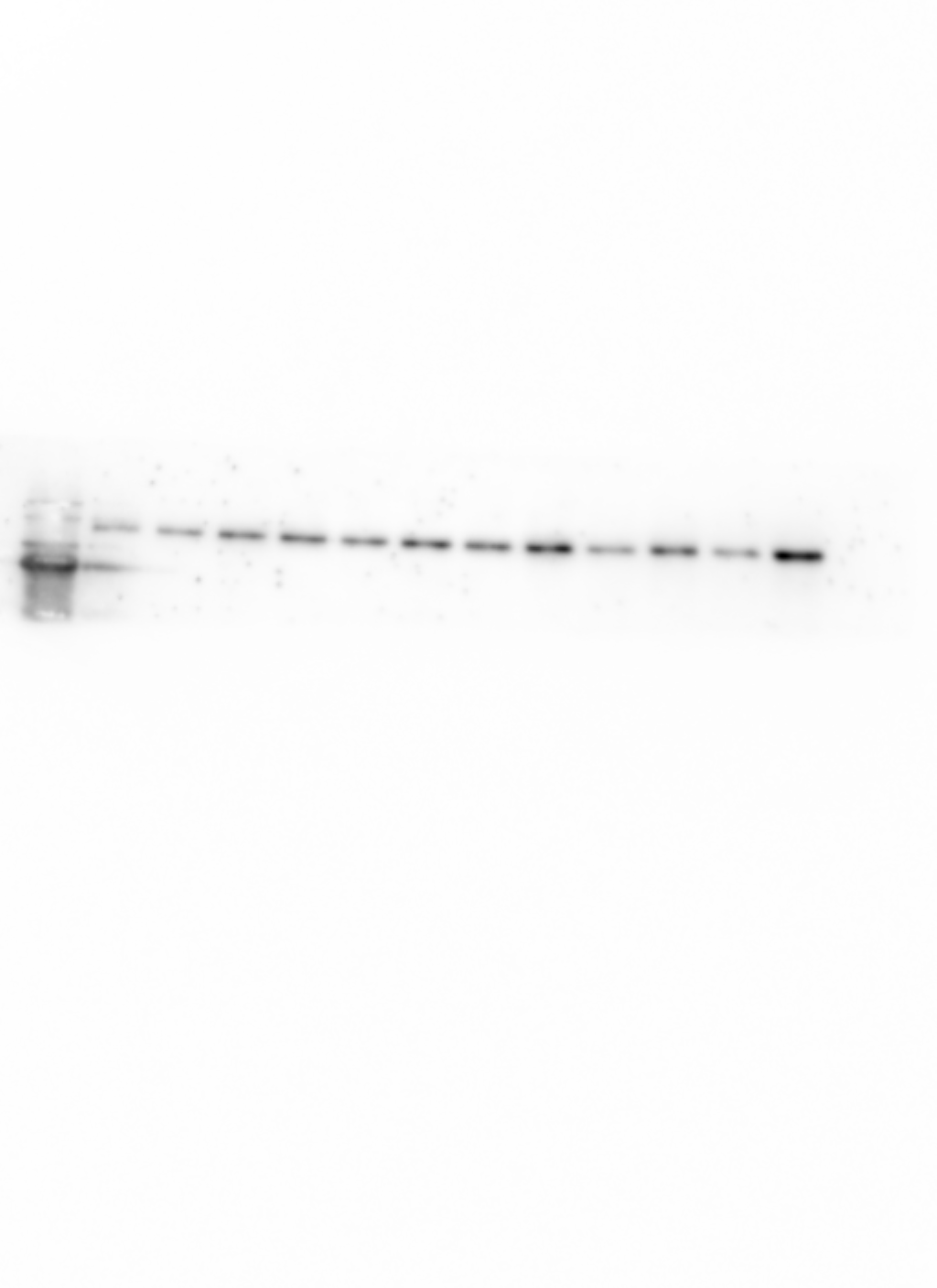

Supplement: Figure 3—figure supplement 1—source data 3. [file elife-75166-fig3-figsupp1-data3.zip › Figure_3-figure_supplement_1-Source_data_3/FigS1C_NLRP3_insoluble_raw.tif]

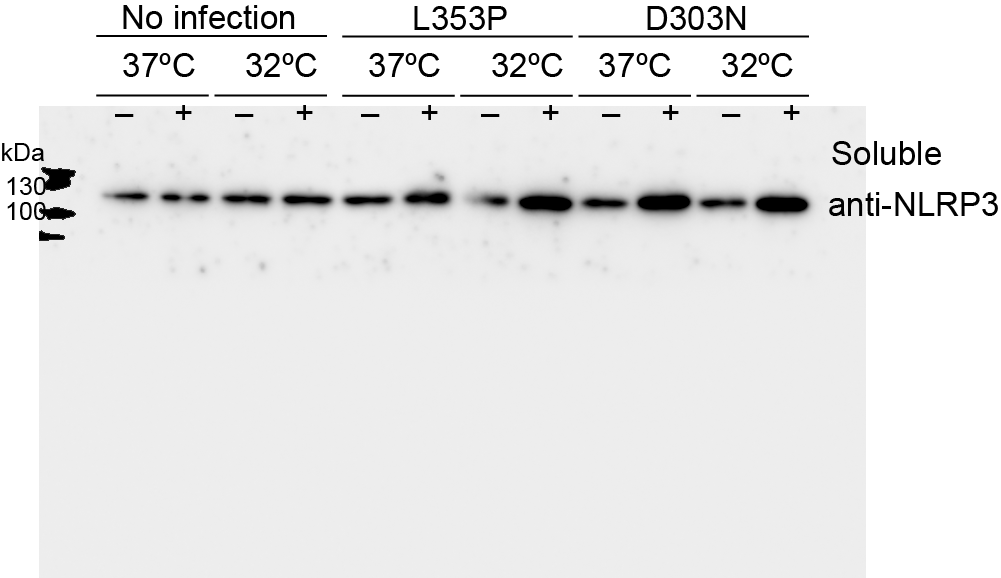

Supplement: Figure 3—figure supplement 1—source data 3. [file elife-75166-fig3-figsupp1-data3.zip › Figure_3-figure_supplement_1-Source_data_3/FigS1C_NLRP3_soluble_labeled.tif]

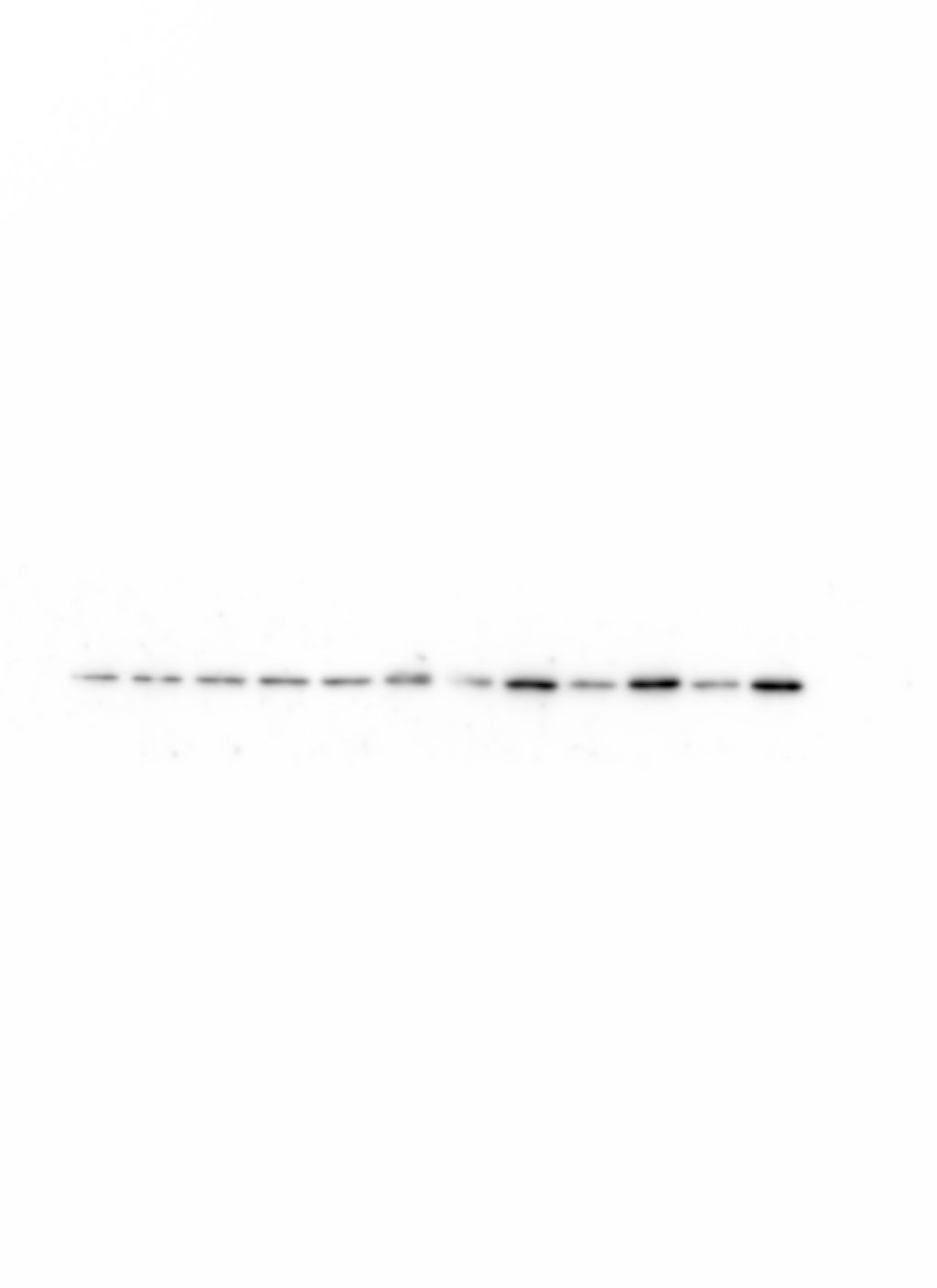

Supplement: Figure 3—figure supplement 1—source data 3. [file elife-75166-fig3-figsupp1-data3.zip › Figure_3-figure_supplement_1-Source_data_3/FigS1C_NLRP3_soluble_raw.tif]

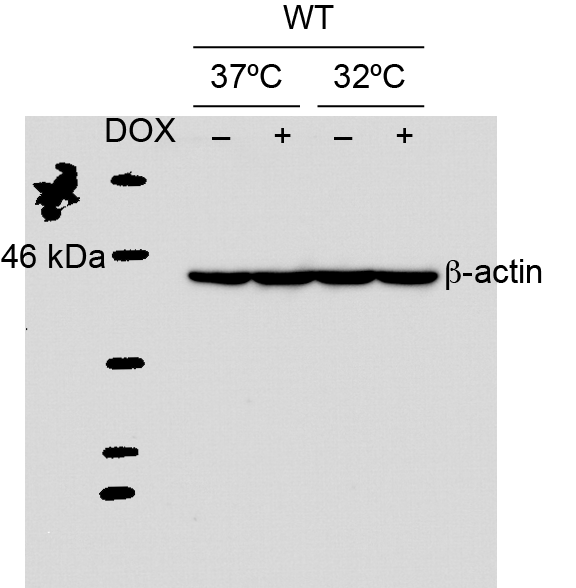

Supplement: Figure 3—figure supplement 1—source data 4. [file elife-75166-fig3-figsupp1-data4.zip › Figure_3-figure_supplement_1-Source_data_4/Fig3S1F_actin_labeled.tif]

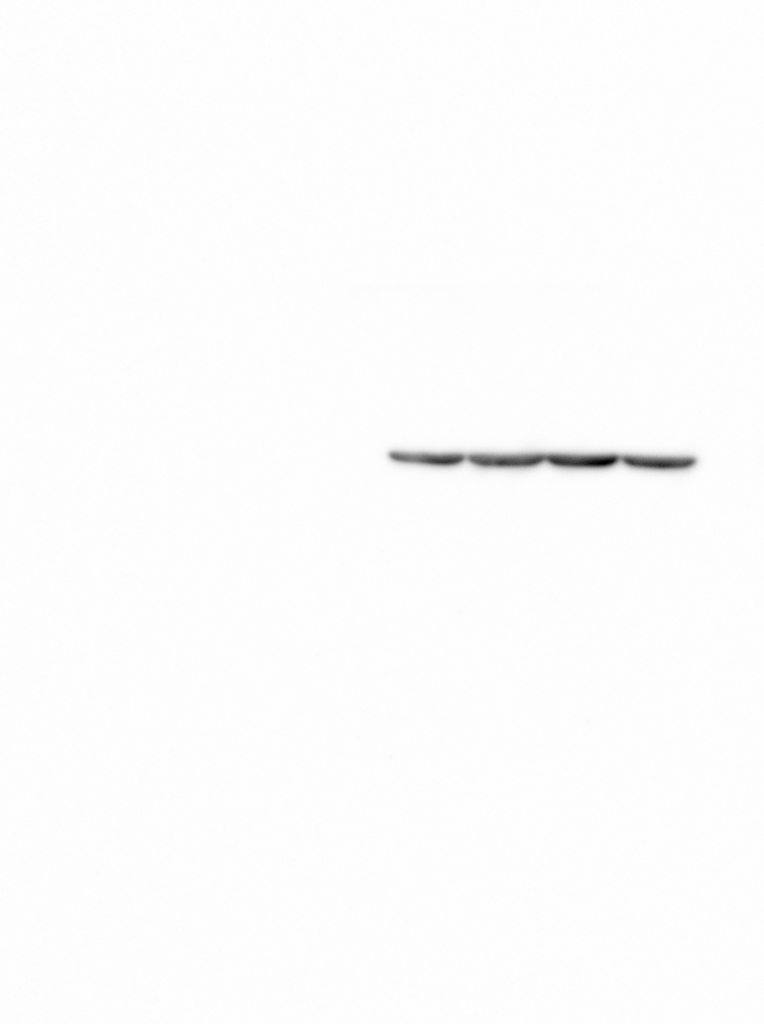

Supplement: Figure 3—figure supplement 1—source data 4. [file elife-75166-fig3-figsupp1-data4.zip › Figure_3-figure_supplement_1-Source_data_4/Fig3S1F_actin_raw.tif]

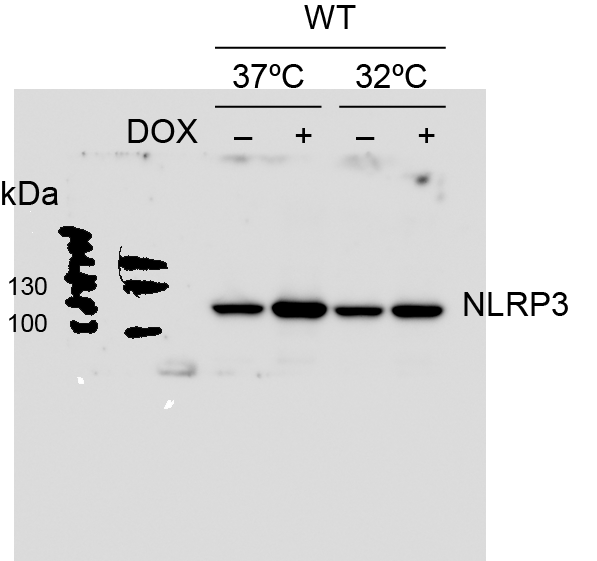

Supplement: Figure 3—figure supplement 1—source data 4. [file elife-75166-fig3-figsupp1-data4.zip › Figure_3-figure_supplement_1-Source_data_4/Fig3S1F_NLRP3_labeled.tif]

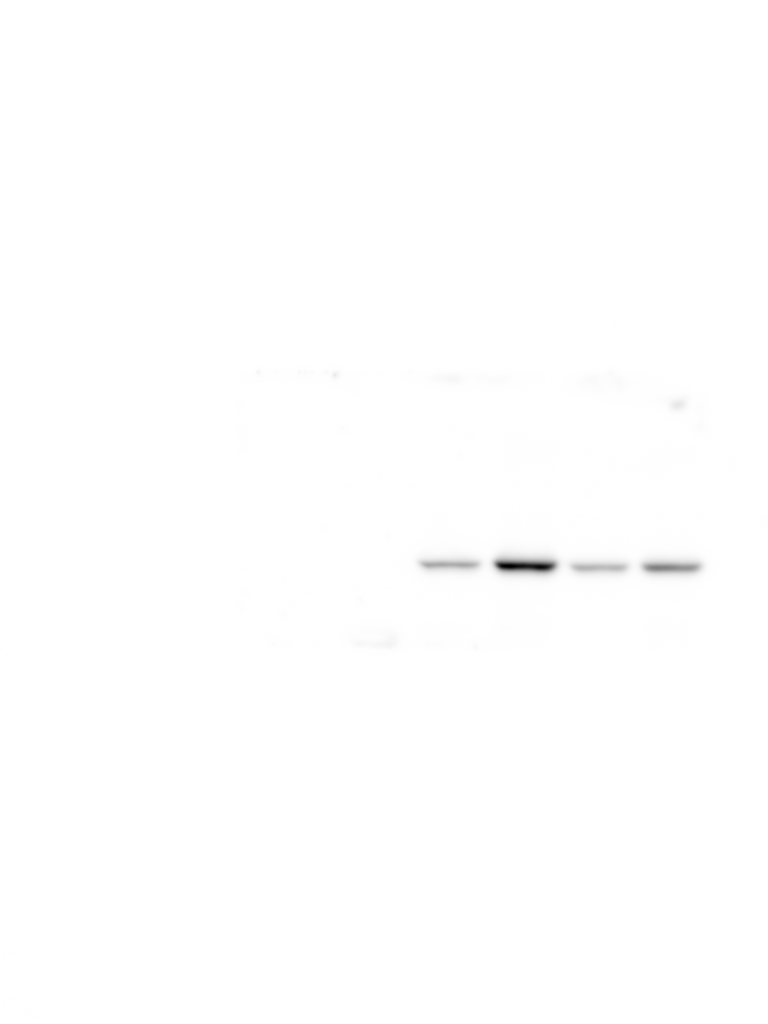

Supplement: Figure 3—figure supplement 1—source data 4. [file elife-75166-fig3-figsupp1-data4.zip › Figure_3-figure_supplement_1-Source_data_4/Fig3S1F_NLRP3_raw.tif]

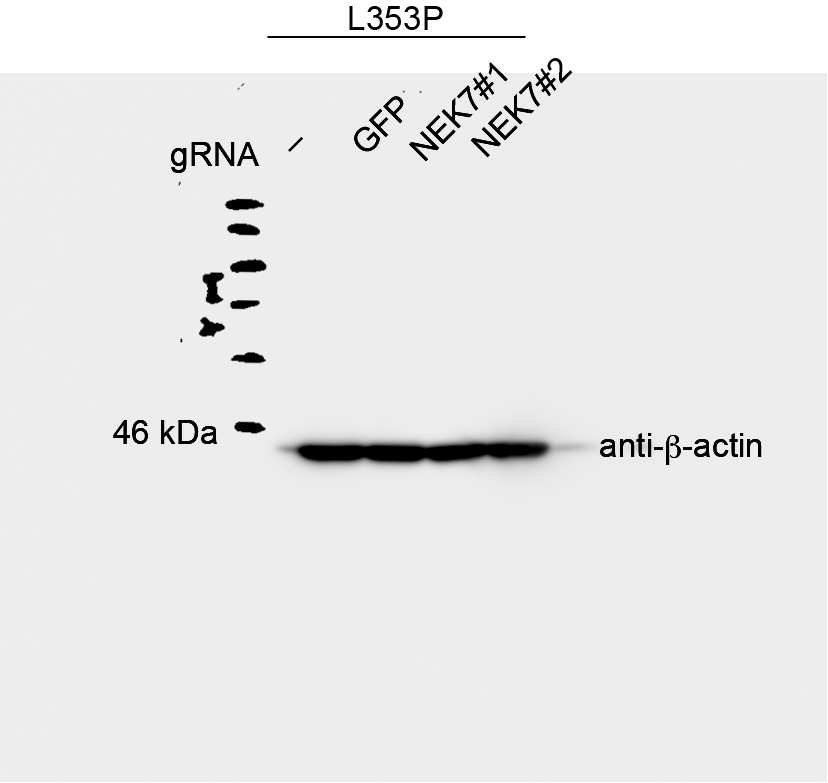

Supplement: Figure 4—figure supplement 1—source data 1. [file elife-75166-fig4-figsupp1-data1.zip › Figure4ΓÇôfigure_supplement1-Source_data1/Fig4_S1C_actin_labeled.tif]

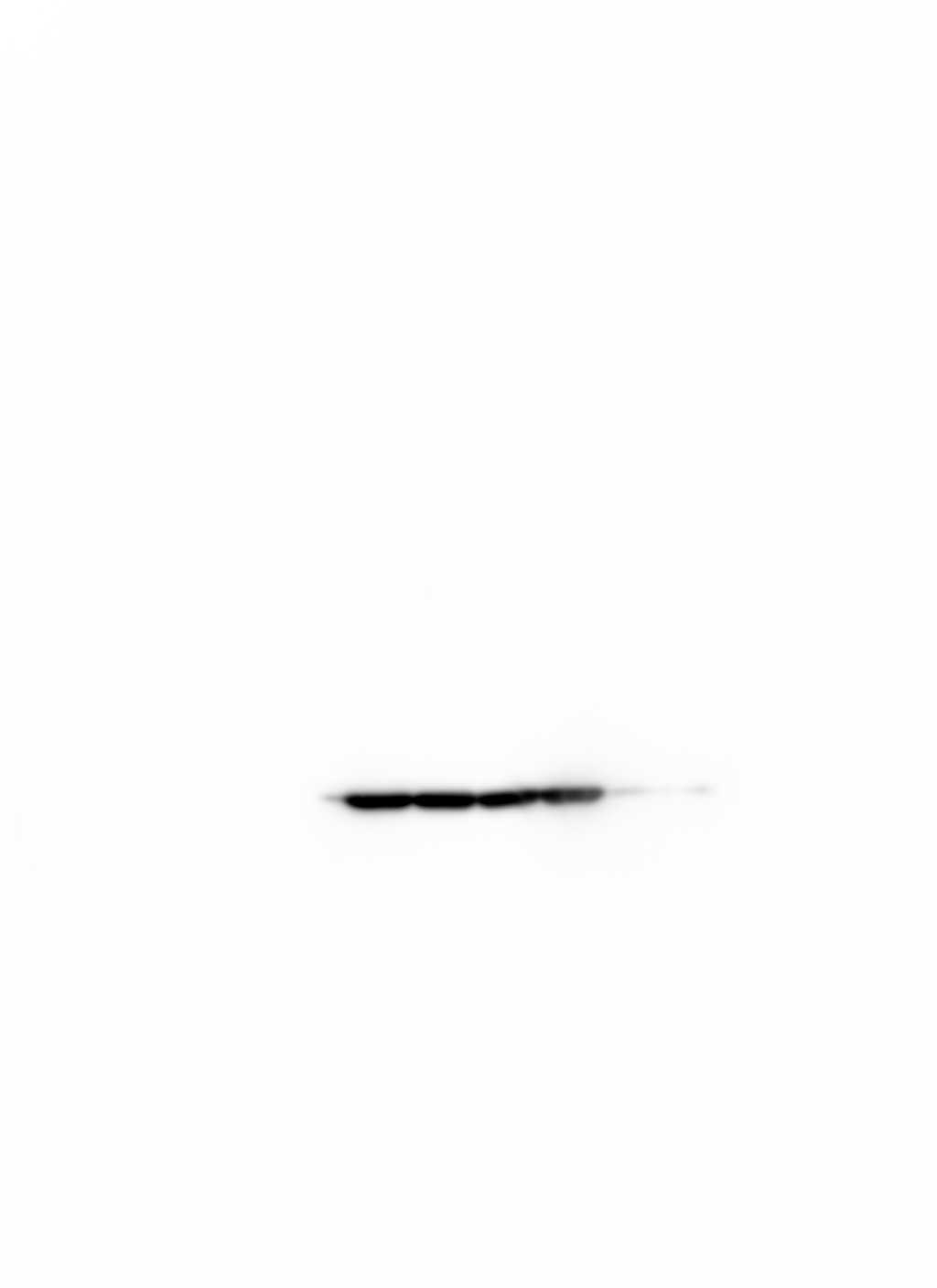

Supplement: Figure 4—figure supplement 1—source data 1. [file elife-75166-fig4-figsupp1-data1.zip › Figure4ΓÇôfigure_supplement1-Source_data1/Fig4_S1C_actin_raw.tif]

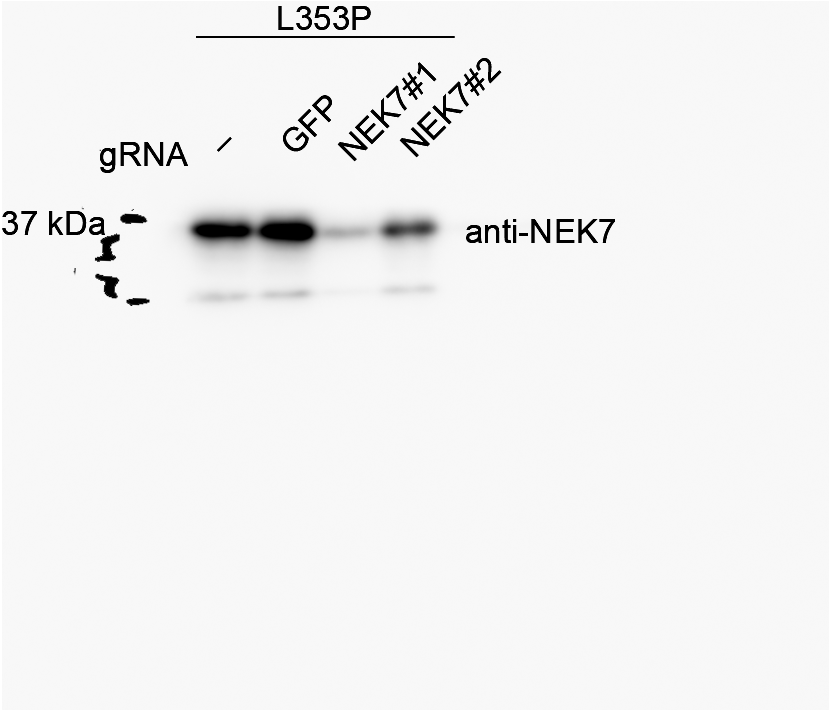

Supplement: Figure 4—figure supplement 1—source data 1. [file elife-75166-fig4-figsupp1-data1.zip › Figure4ΓÇôfigure_supplement1-Source_data1/Fig4S1C_NEK7_labeled.tif]

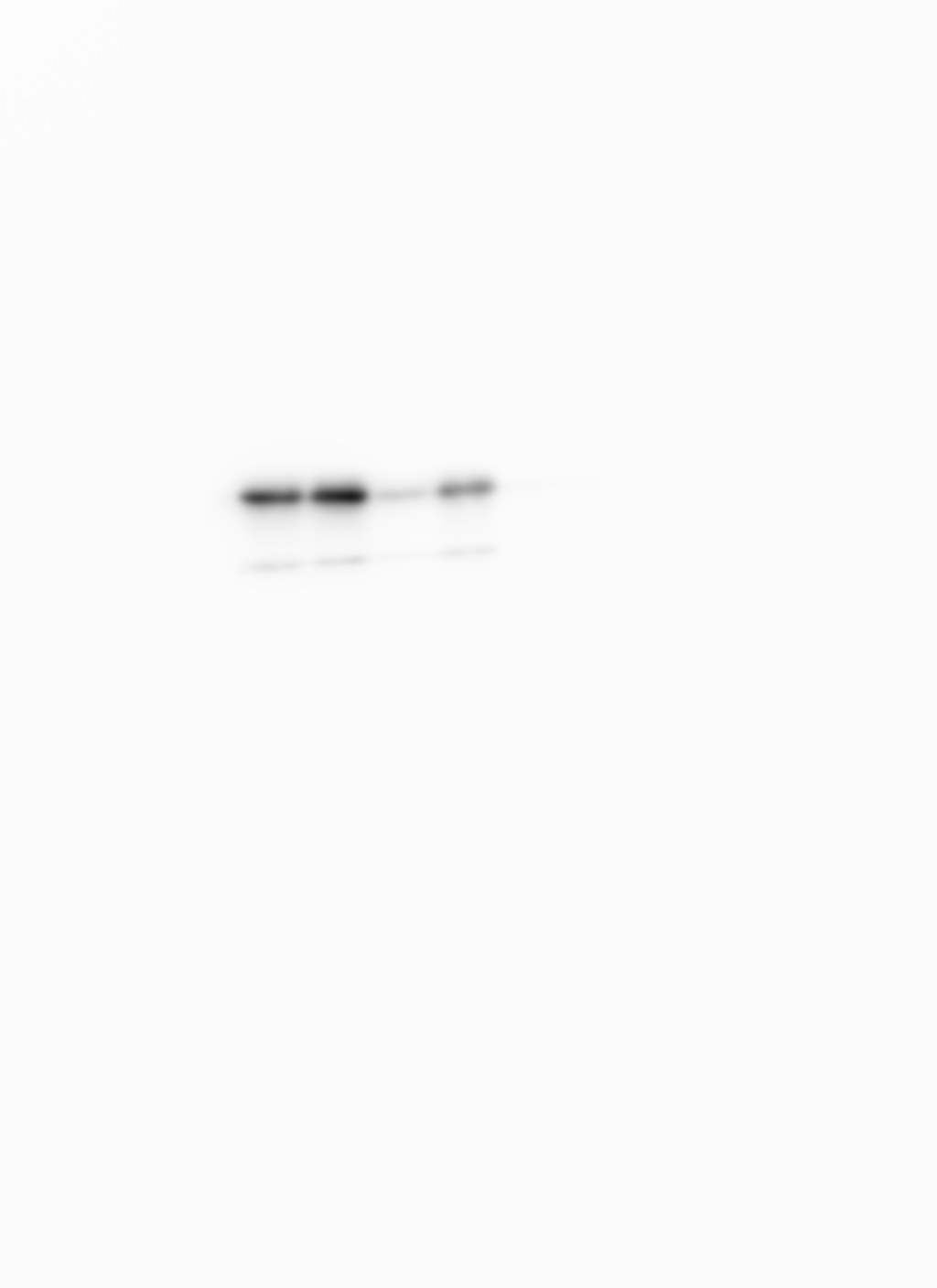

Supplement: Figure 4—figure supplement 1—source data 1. [file elife-75166-fig4-figsupp1-data1.zip › Figure4ΓÇôfigure_supplement1-Source_data1/Fig4S1C_NEK7_raw.tif]

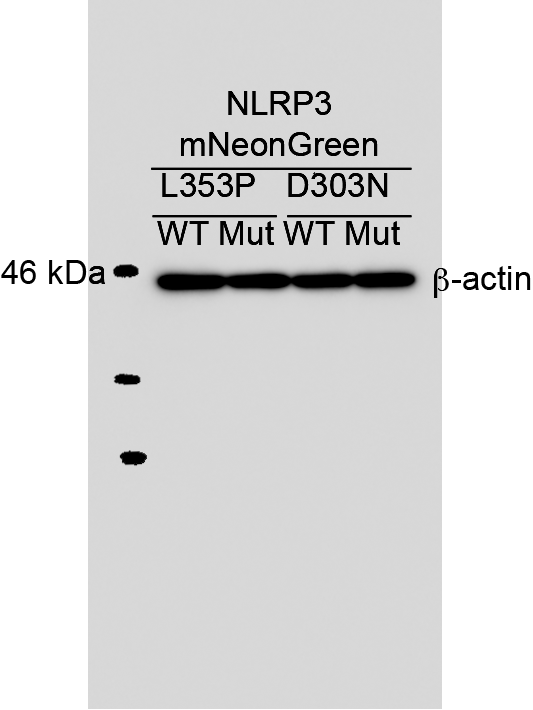

Supplement: Figure 4—figure supplement 1—source data 2. [file elife-75166-fig4-figsupp1-data2.zip › Figure4ΓÇôfigure_supplement1-Source_data2/Fig4S1F_actin_labeled.tif]

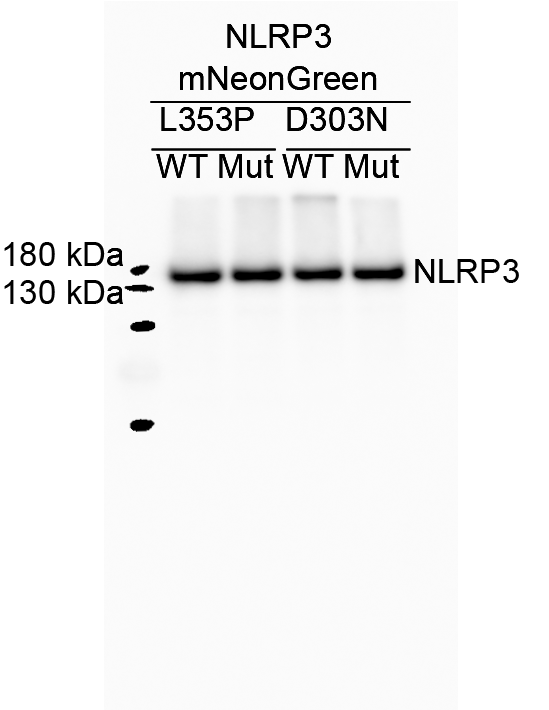

Supplement: Figure 4—figure supplement 1—source data 2. [file elife-75166-fig4-figsupp1-data2.zip › Figure4ΓÇôfigure_supplement1-Source_data2/Fig4S1F_NLRP3_labeled.tif]

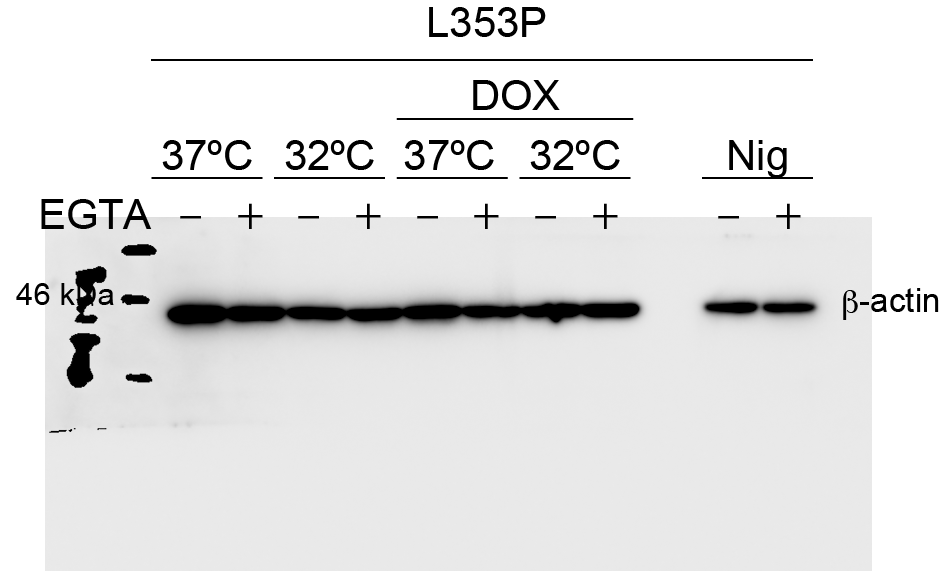

Supplement: Figure 5—source data 1. [file elife-75166-fig5-data1.zip › Figure_5-Source_data_1/Fig5C_actin_labeled.tif]

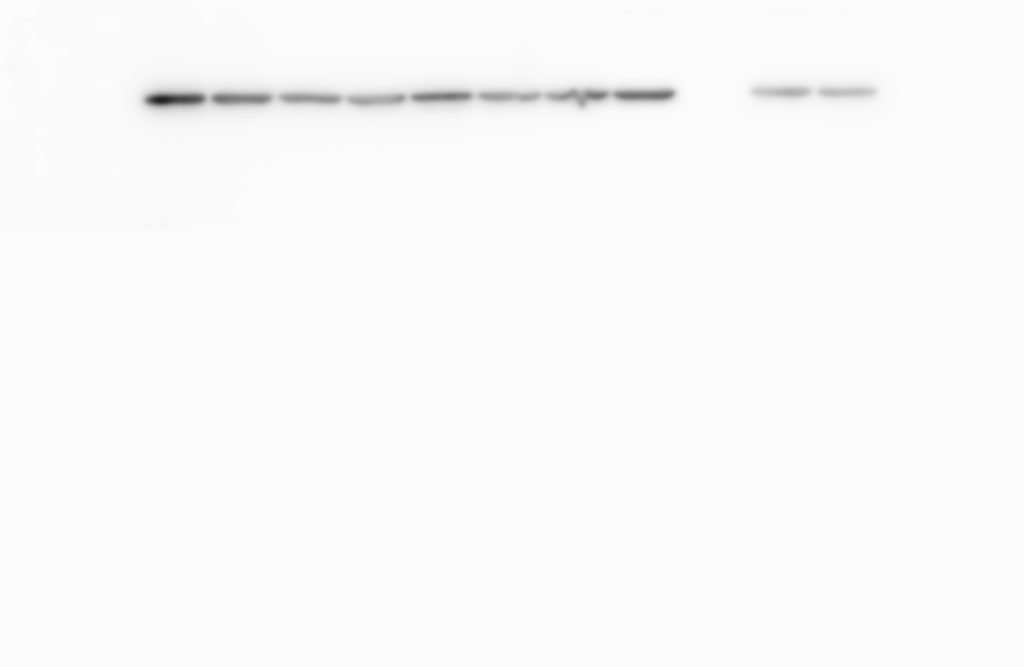

Supplement: Figure 5—source data 1. [file elife-75166-fig5-data1.zip › Figure_5-Source_data_1/Fig5C_actin_raw.tif]

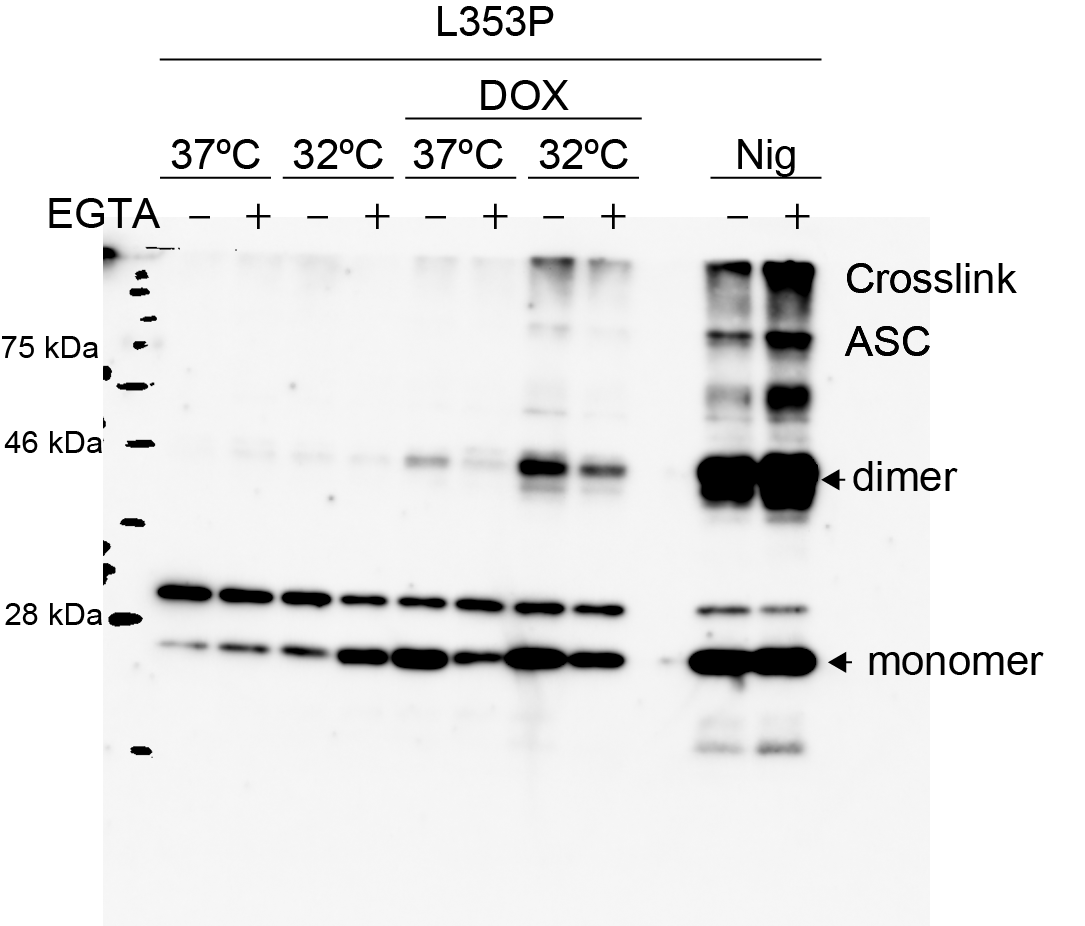

Supplement: Figure 5—source data 1. [file elife-75166-fig5-data1.zip › Figure_5-Source_data_1/Fig5C_ASC_crosslink_labeled.tif]

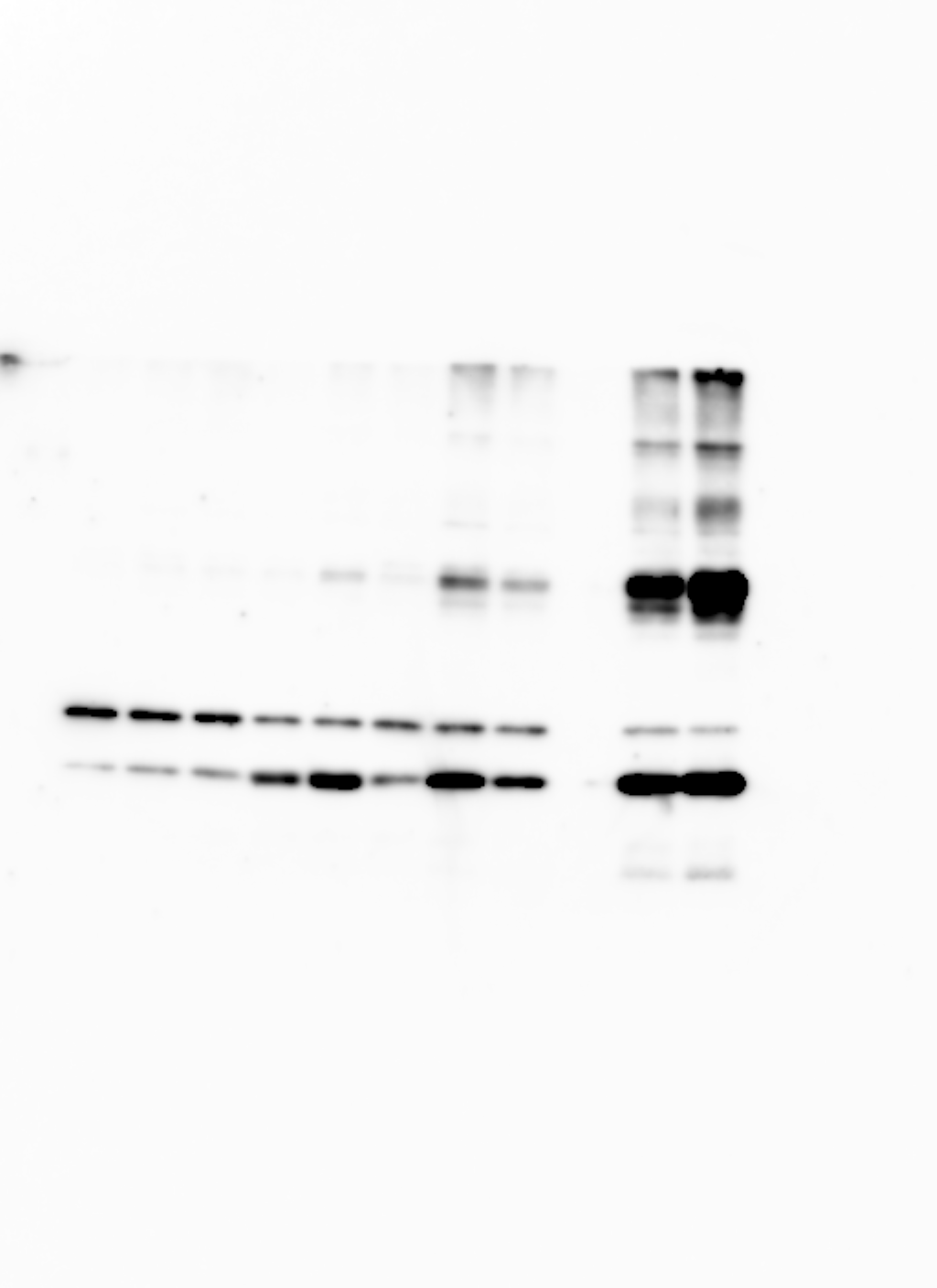

Supplement: Figure 5—source data 1. [file elife-75166-fig5-data1.zip › Figure_5-Source_data_1/Fig5C_ASC_crosslink_raw.tif]

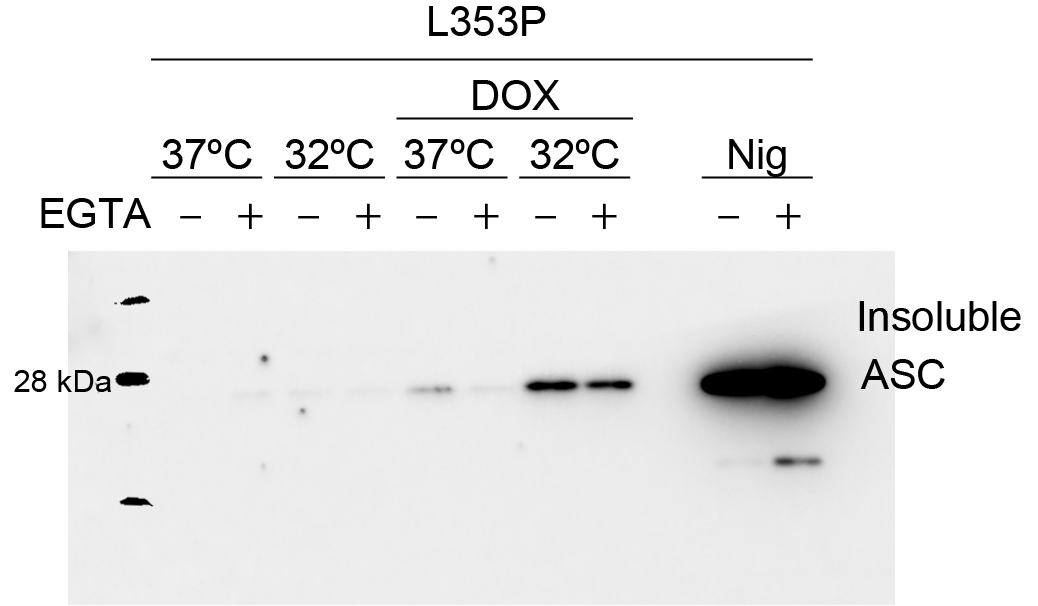

Supplement: Figure 5—source data 1. [file elife-75166-fig5-data1.zip › Figure_5-Source_data_1/Fig5C_ASC_insoluble_labeled.tif]

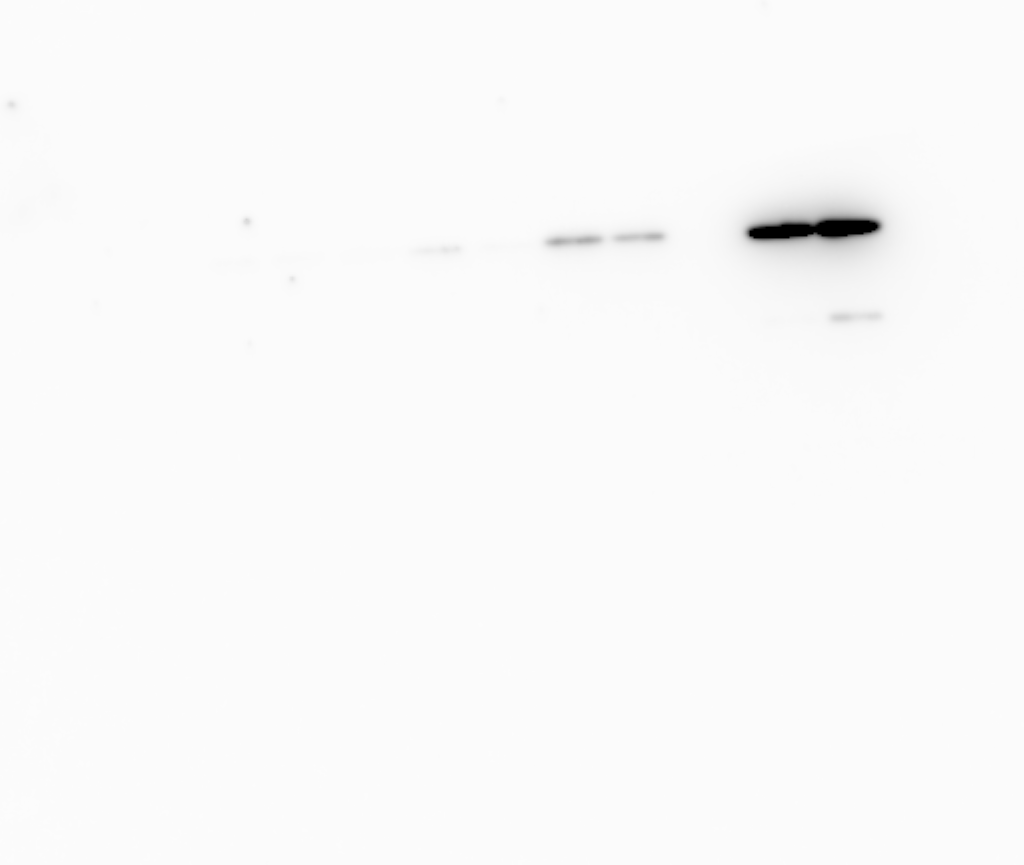

Supplement: Figure 5—source data 1. [file elife-75166-fig5-data1.zip › Figure_5-Source_data_1/Fig5C_ASC_insoluble_raw.tif]

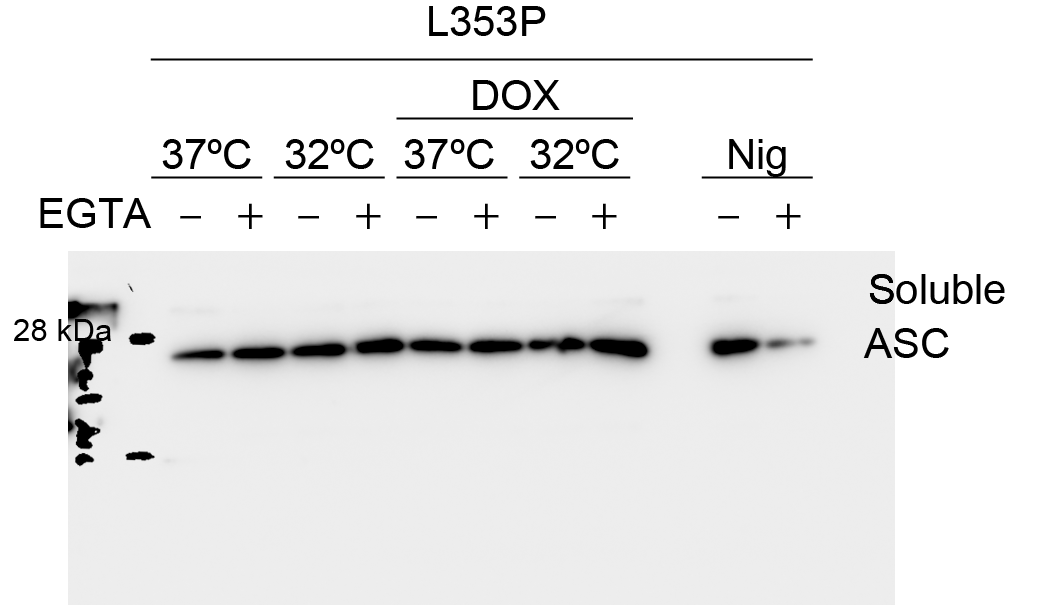

Supplement: Figure 5—source data 1. [file elife-75166-fig5-data1.zip › Figure_5-Source_data_1/Fig5C_ASC_soluble_labeled.tif]

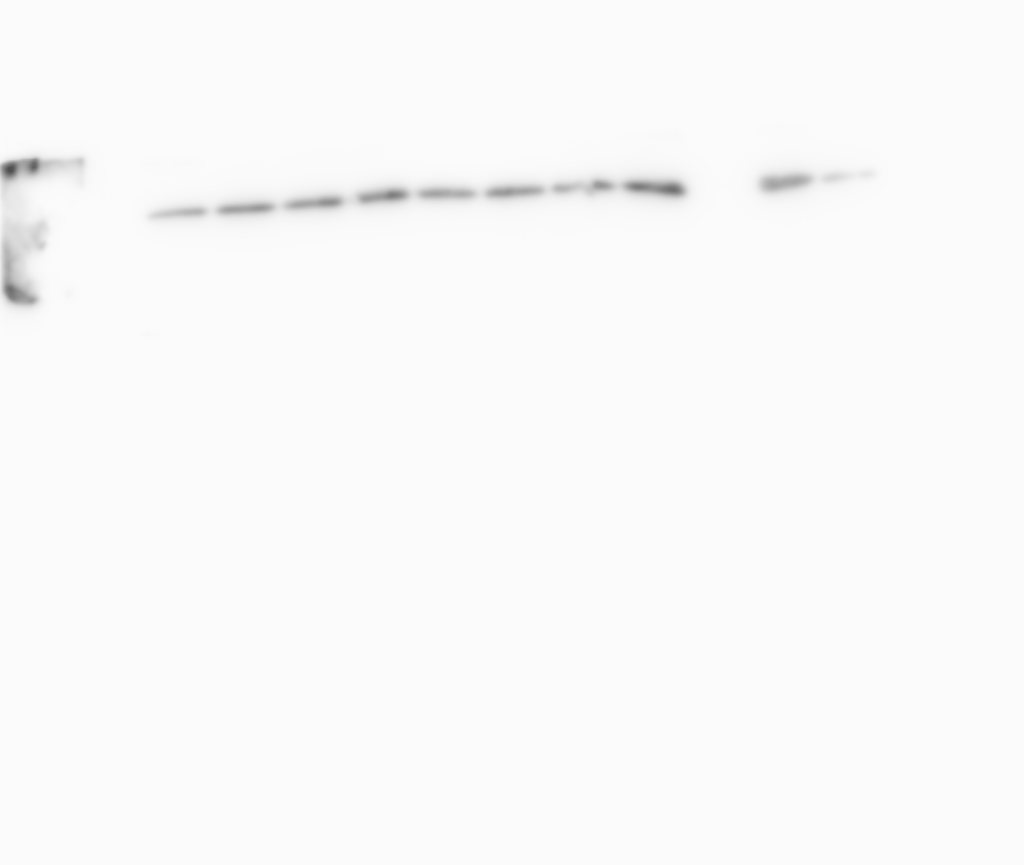

Supplement: Figure 5—source data 1. [file elife-75166-fig5-data1.zip › Figure_5-Source_data_1/Fig5C_ASC_soluble_raw.tif]

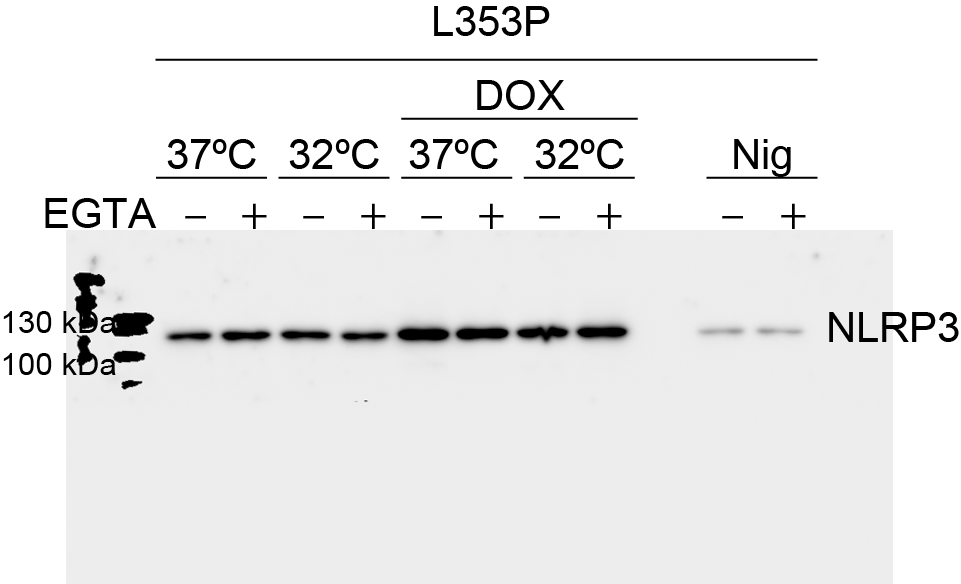

Supplement: Figure 5—source data 1. [file elife-75166-fig5-data1.zip › Figure_5-Source_data_1/Fig5C_NLRP3_labeled.tif]

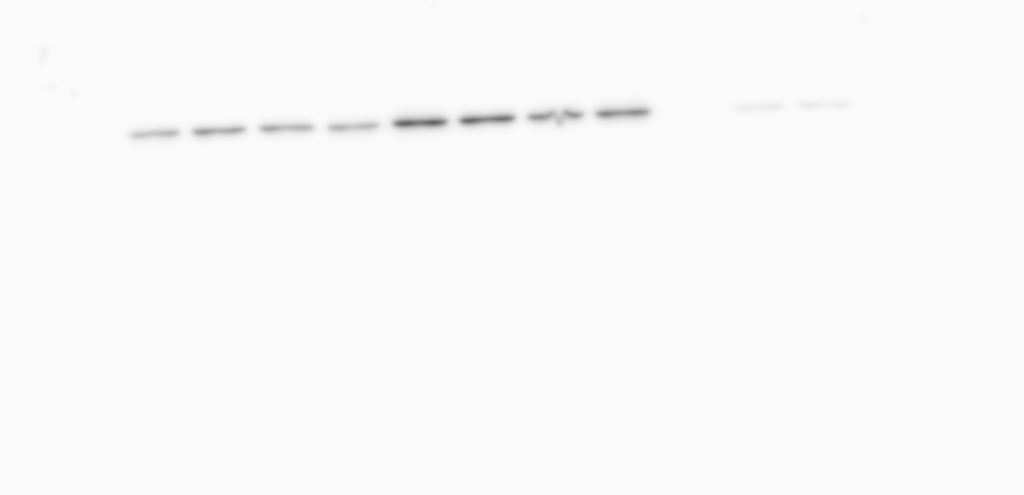

Supplement: Figure 5—source data 1. [file elife-75166-fig5-data1.zip › Figure_5-Source_data_1/Fig5C_NLRP3_raw.tif]

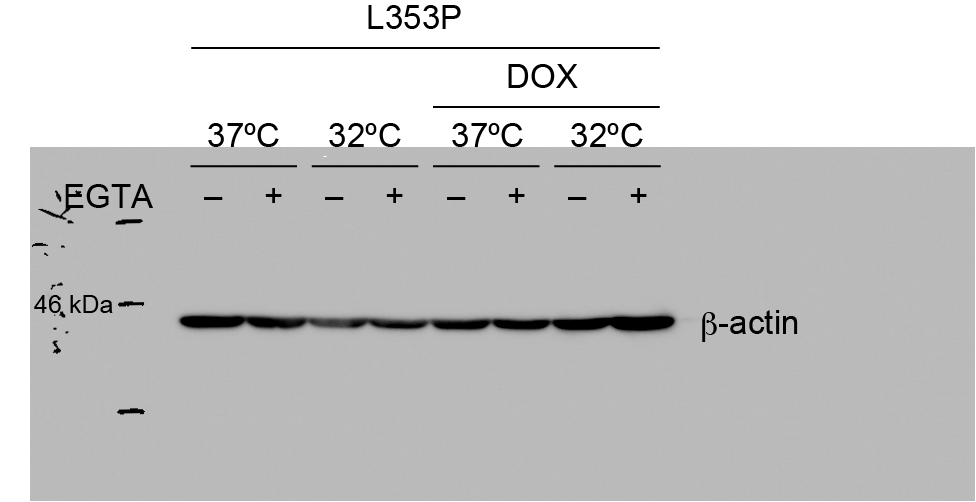

Supplement: Figure 5—figure supplement 1—source data 1. [file elife-75166-fig5-figsupp1-data1.zip › Figure_5-figure_supplement_2-Source_data_1/Fig5S1C_actin_labeled.tif]

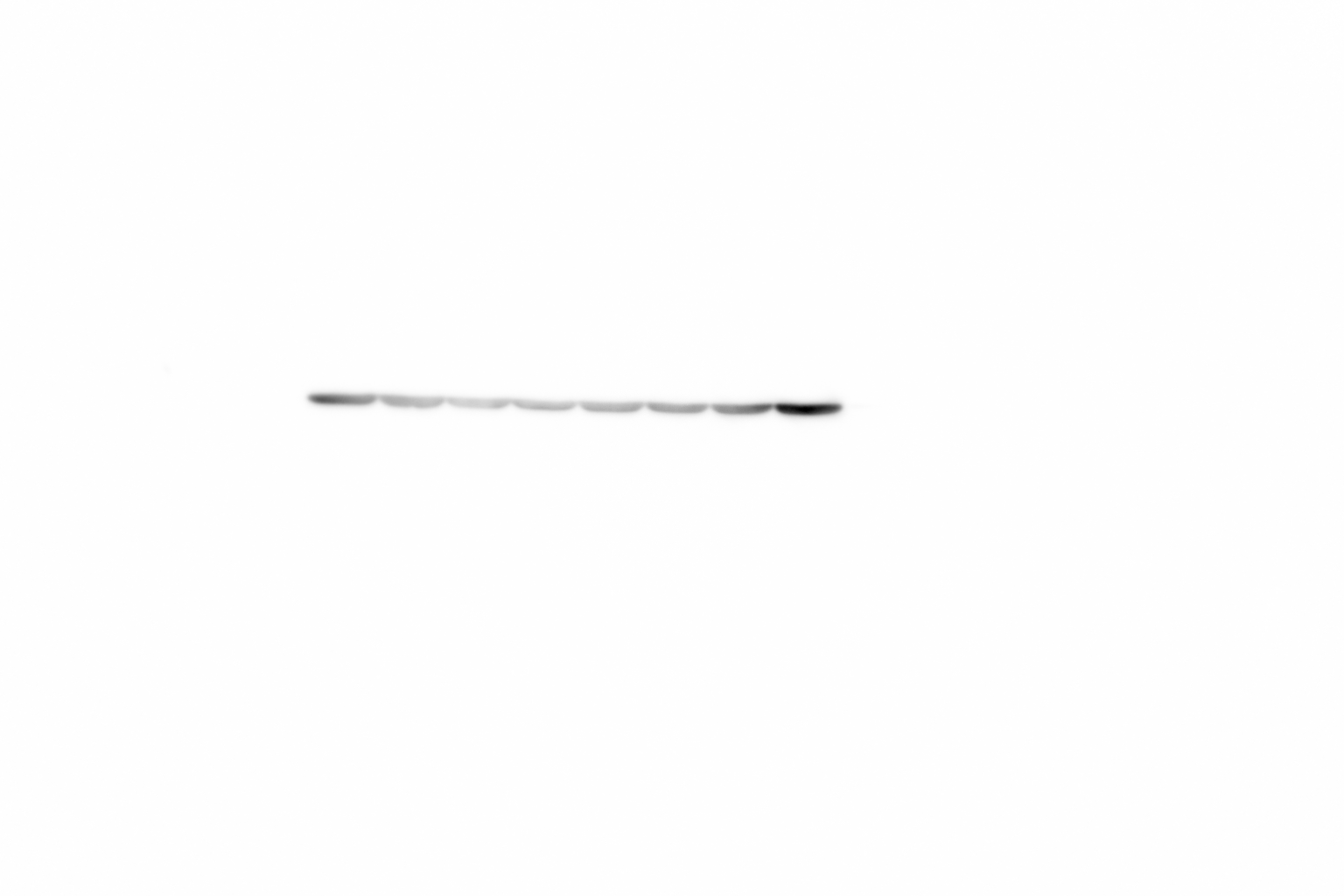

Supplement: Figure 5—figure supplement 1—source data 1. [file elife-75166-fig5-figsupp1-data1.zip › Figure_5-figure_supplement_2-Source_data_1/Fig5S1C_actin_raw.tif]

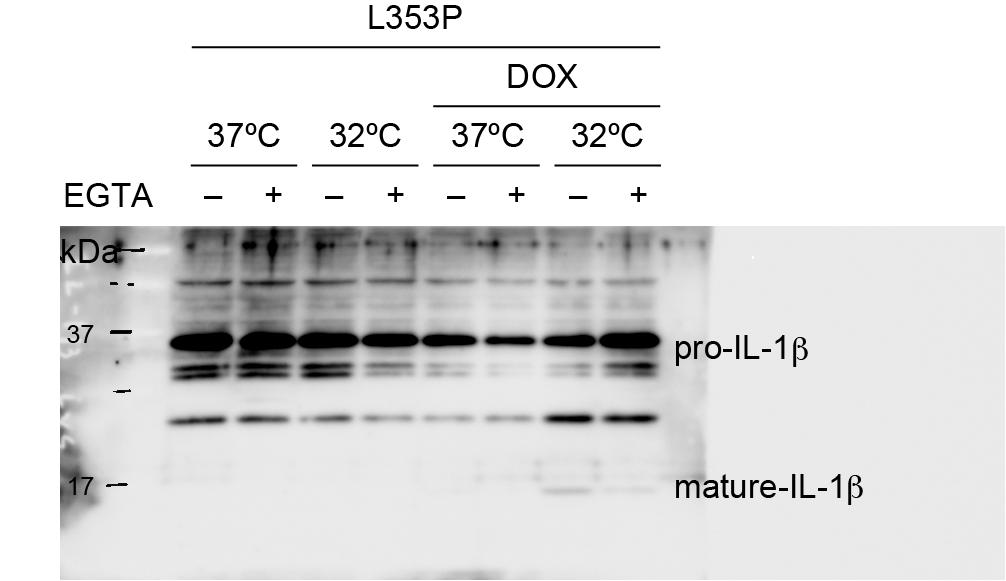

Supplement: Figure 5—figure supplement 1—source data 1. [file elife-75166-fig5-figsupp1-data1.zip › Figure_5-figure_supplement_2-Source_data_1/Fig5S1C_IL-1beta_ly_labeled.tif]

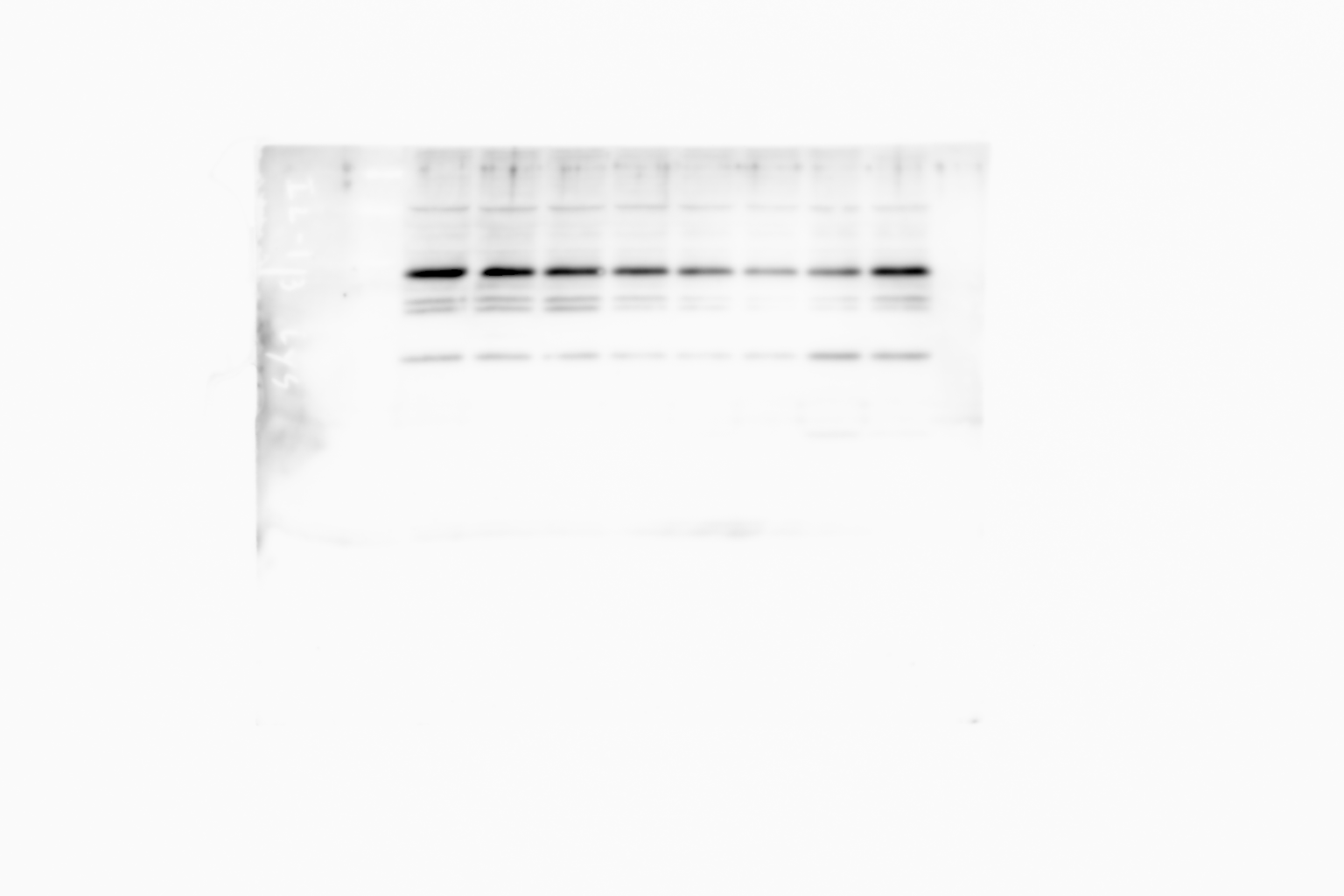

Supplement: Figure 5—figure supplement 1—source data 1. [file elife-75166-fig5-figsupp1-data1.zip › Figure_5-figure_supplement_2-Source_data_1/Fig5S1C_IL-1beta_raw.tif]

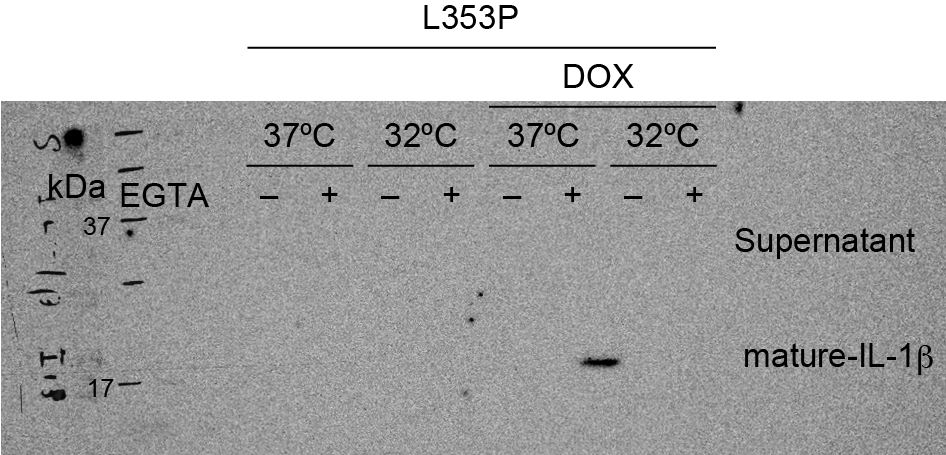

Supplement: Figure 5—figure supplement 1—source data 1. [file elife-75166-fig5-figsupp1-data1.zip › Figure_5-figure_supplement_2-Source_data_1/Fig5S1C_IL-1beta_sup_labeled.tif]

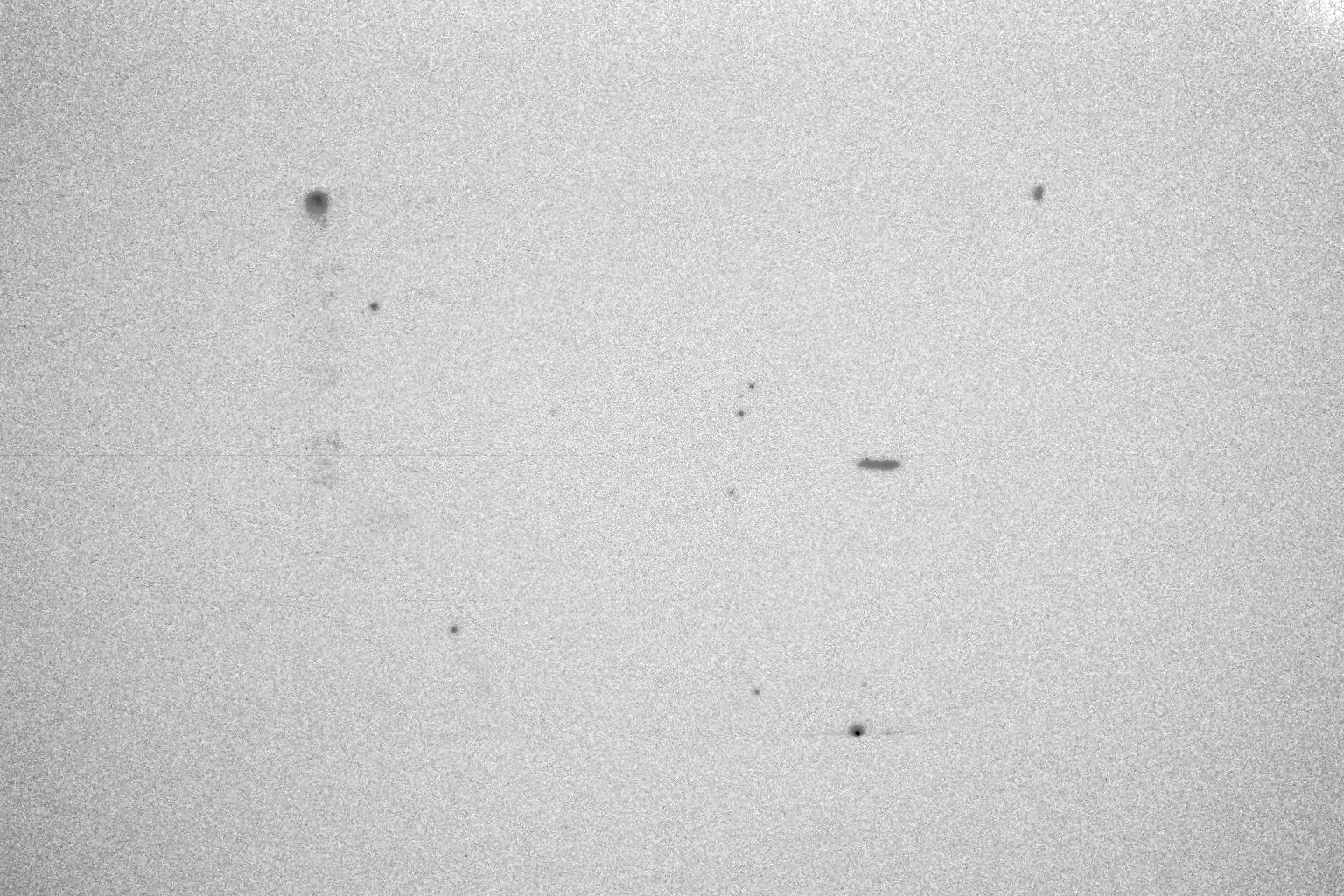

Supplement: Figure 5—figure supplement 1—source data 1. [file elife-75166-fig5-figsupp1-data1.zip › Figure_5-figure_supplement_2-Source_data_1/Fig5S1C_IL-1beta_sup_raw.tif]

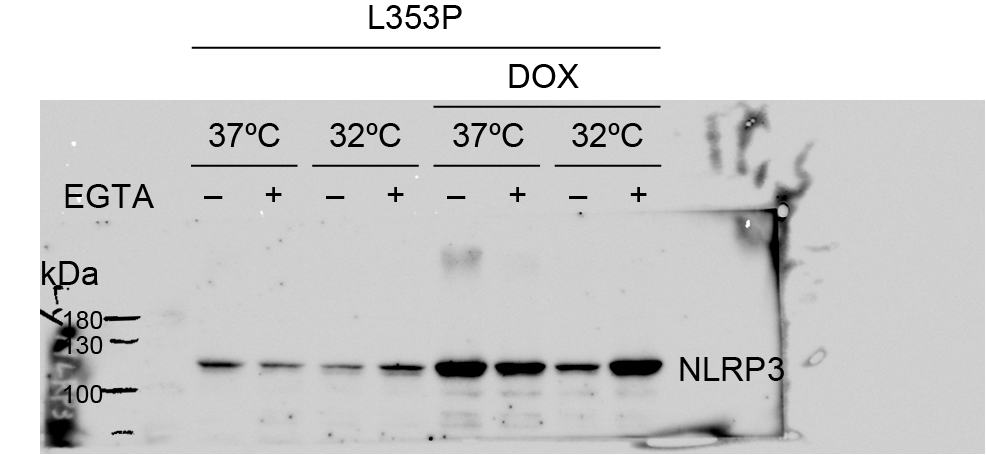

Supplement: Figure 5—figure supplement 1—source data 1. [file elife-75166-fig5-figsupp1-data1.zip › Figure_5-figure_supplement_2-Source_data_1/Fig5S1C_NLRP3_Ly_labeled.tif]

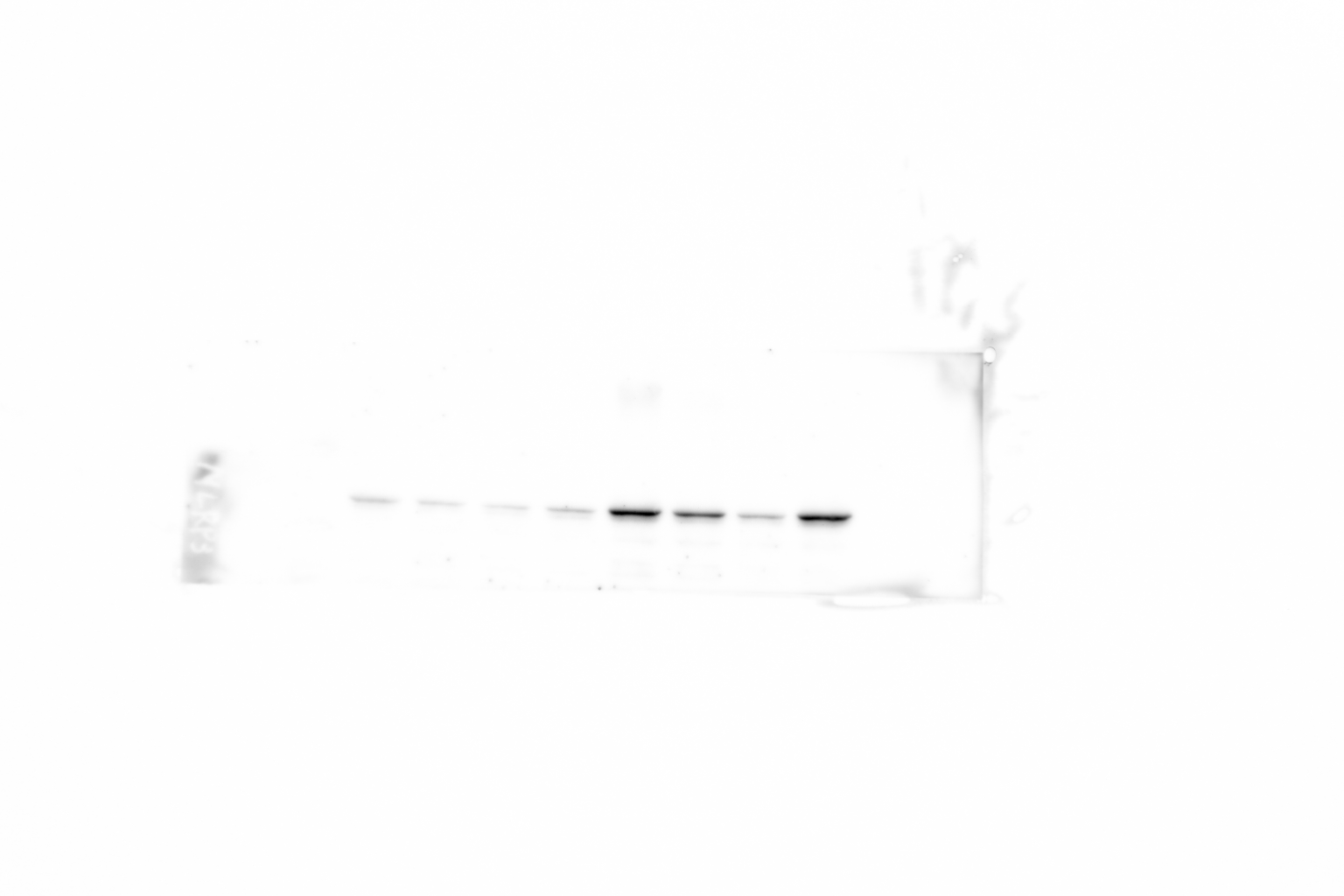

Supplement: Figure 5—figure supplement 1—source data 1. [file elife-75166-fig5-figsupp1-data1.zip › Figure_5-figure_supplement_2-Source_data_1/Fig5S1C_NLRP3_Ly_raw.tif]

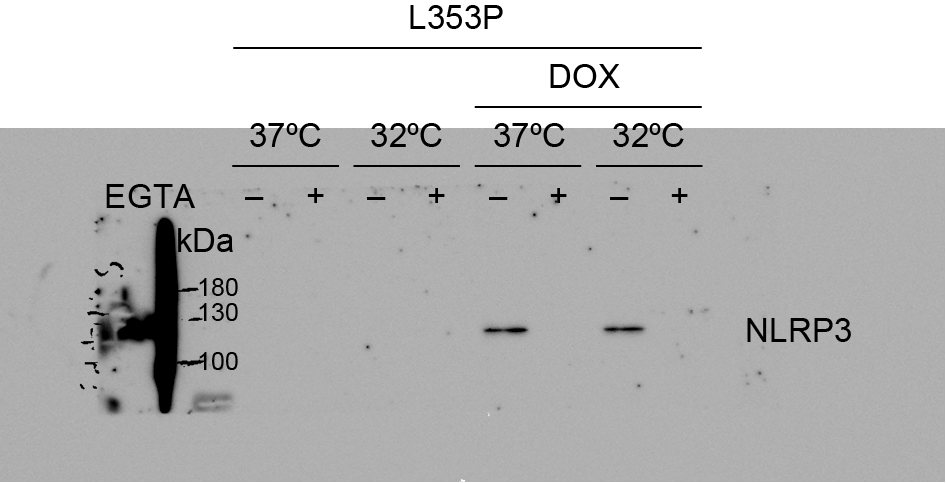

Supplement: Figure 5—figure supplement 1—source data 1. [file elife-75166-fig5-figsupp1-data1.zip › Figure_5-figure_supplement_2-Source_data_1/Fig5S1C_NLRP3_Sup_labeled.tif]

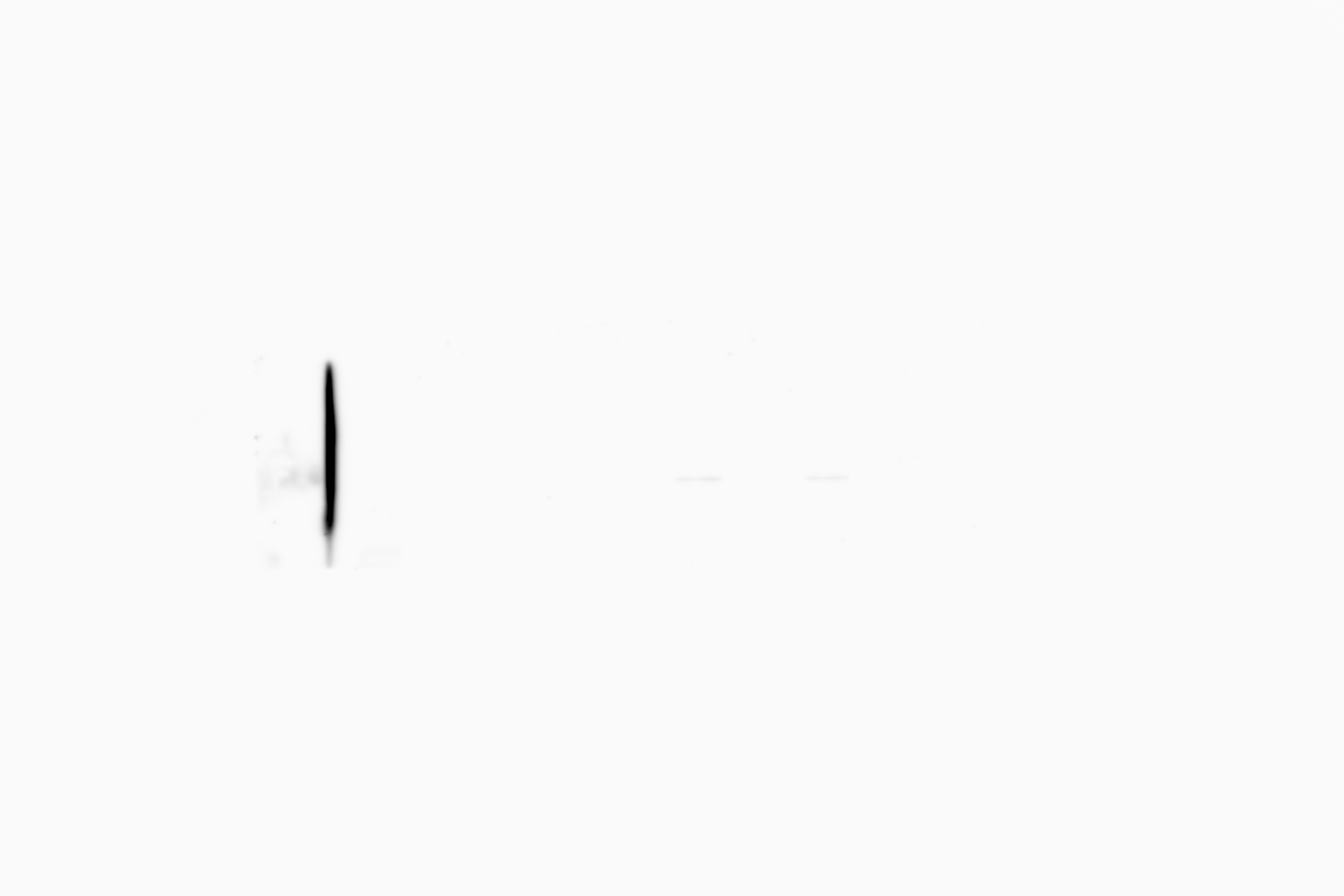

Supplement: Figure 5—figure supplement 1—source data 1. [file elife-75166-fig5-figsupp1-data1.zip › Figure_5-figure_supplement_2-Source_data_1/Fig5S1C_NLRP3_Sup_raw.tif]

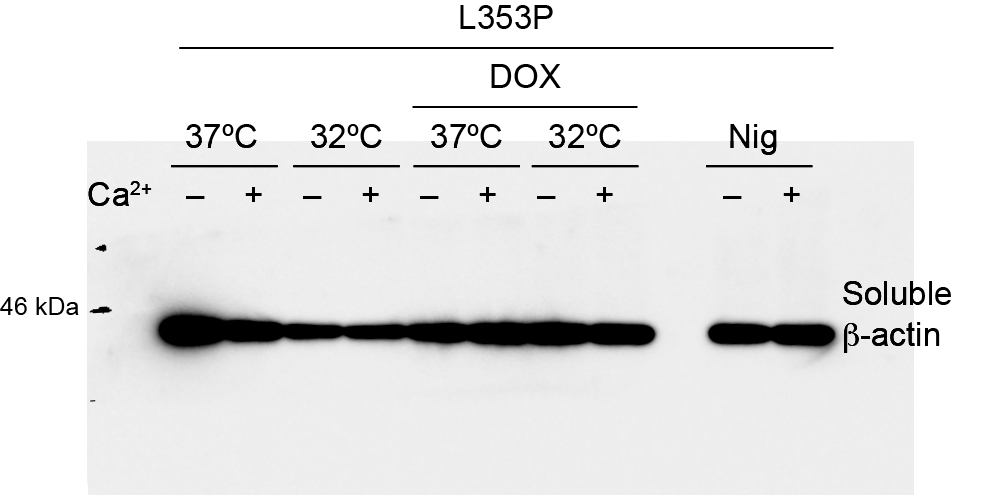

Supplement: Figure 5—figure supplement 1—source data 2. [file elife-75166-fig5-figsupp1-data2.zip › Figure_5-figure_supplement_2-Source_data_2/Fig5S1D_actin_labeled.tif]

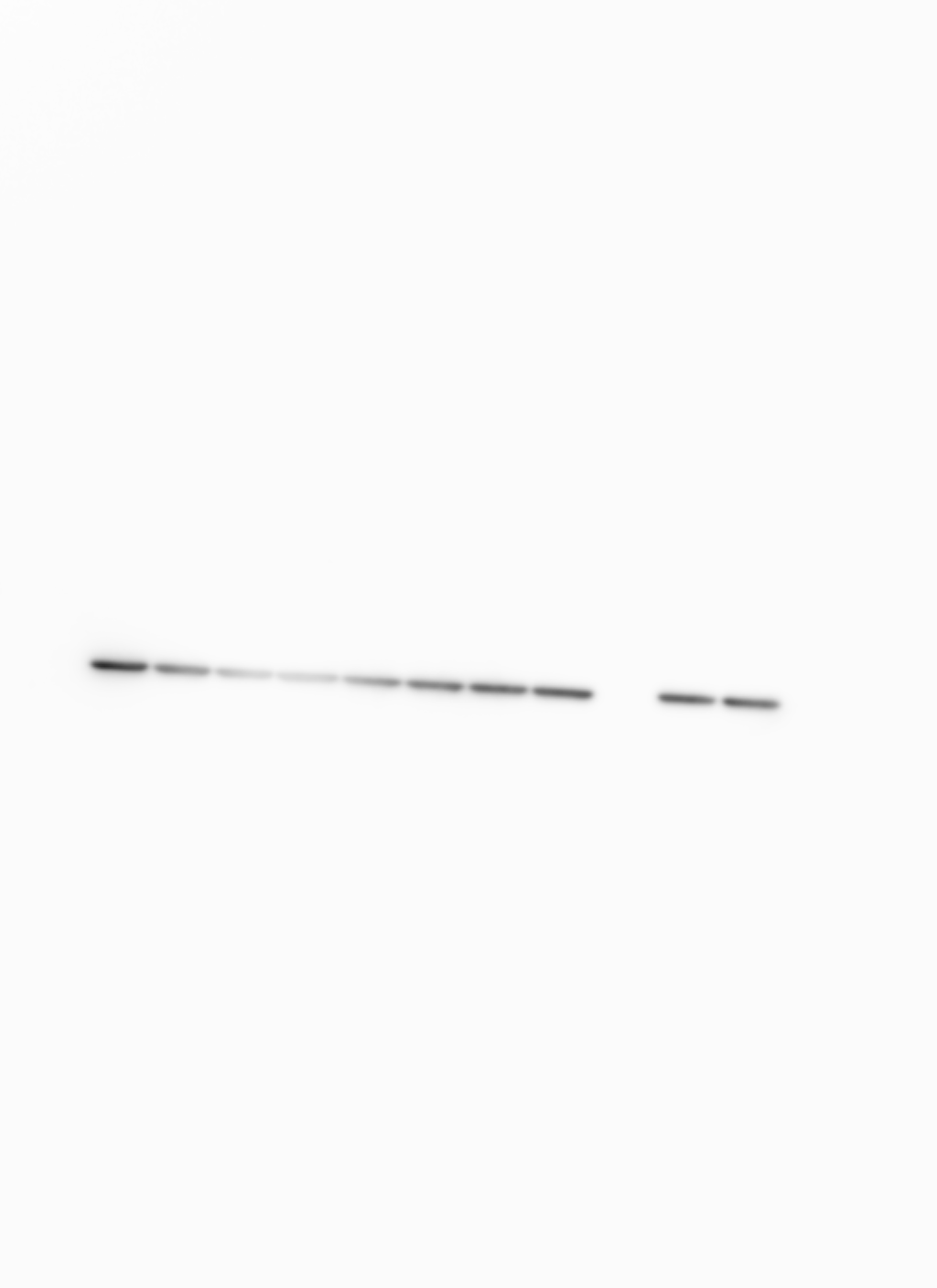

Supplement: Figure 5—figure supplement 1—source data 2. [file elife-75166-fig5-figsupp1-data2.zip › Figure_5-figure_supplement_2-Source_data_2/Fig5S1D_actin_raw.tif]

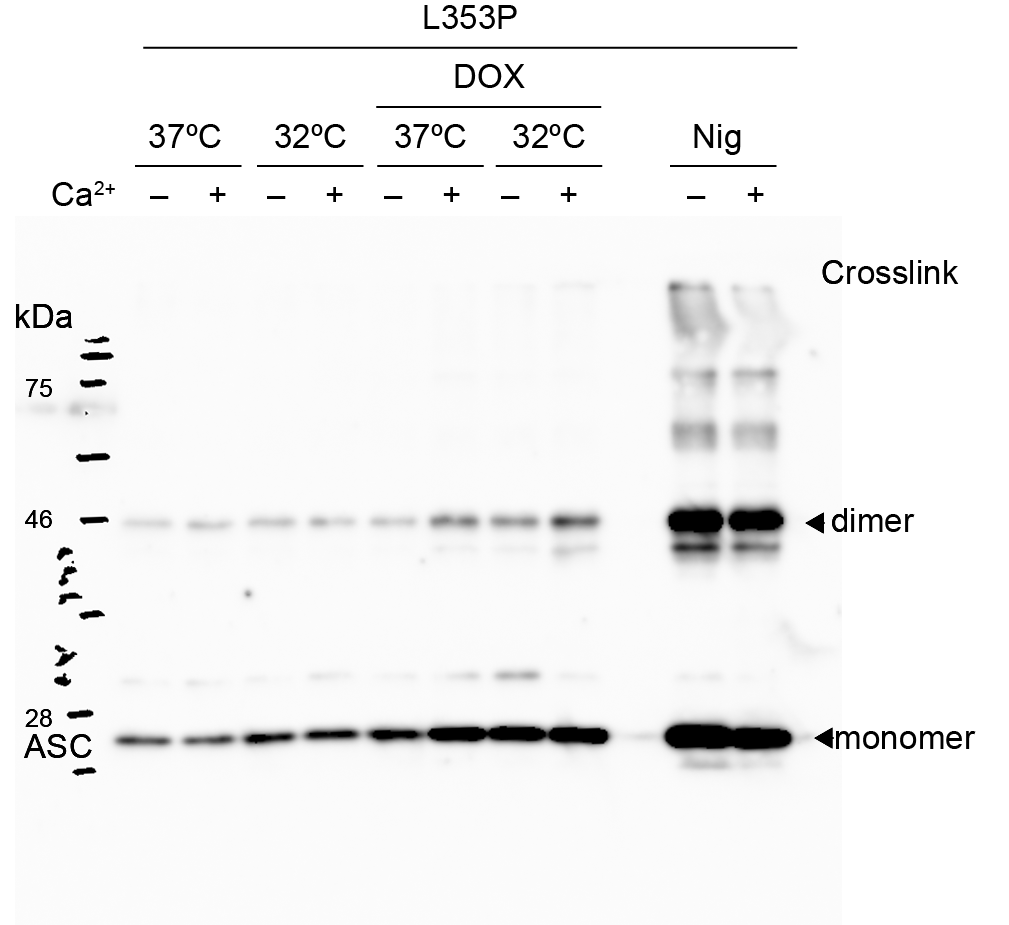

Supplement: Figure 5—figure supplement 1—source data 2. [file elife-75166-fig5-figsupp1-data2.zip › Figure_5-figure_supplement_2-Source_data_2/Fig5S1D_ASC_crosslink_labeled.tif]

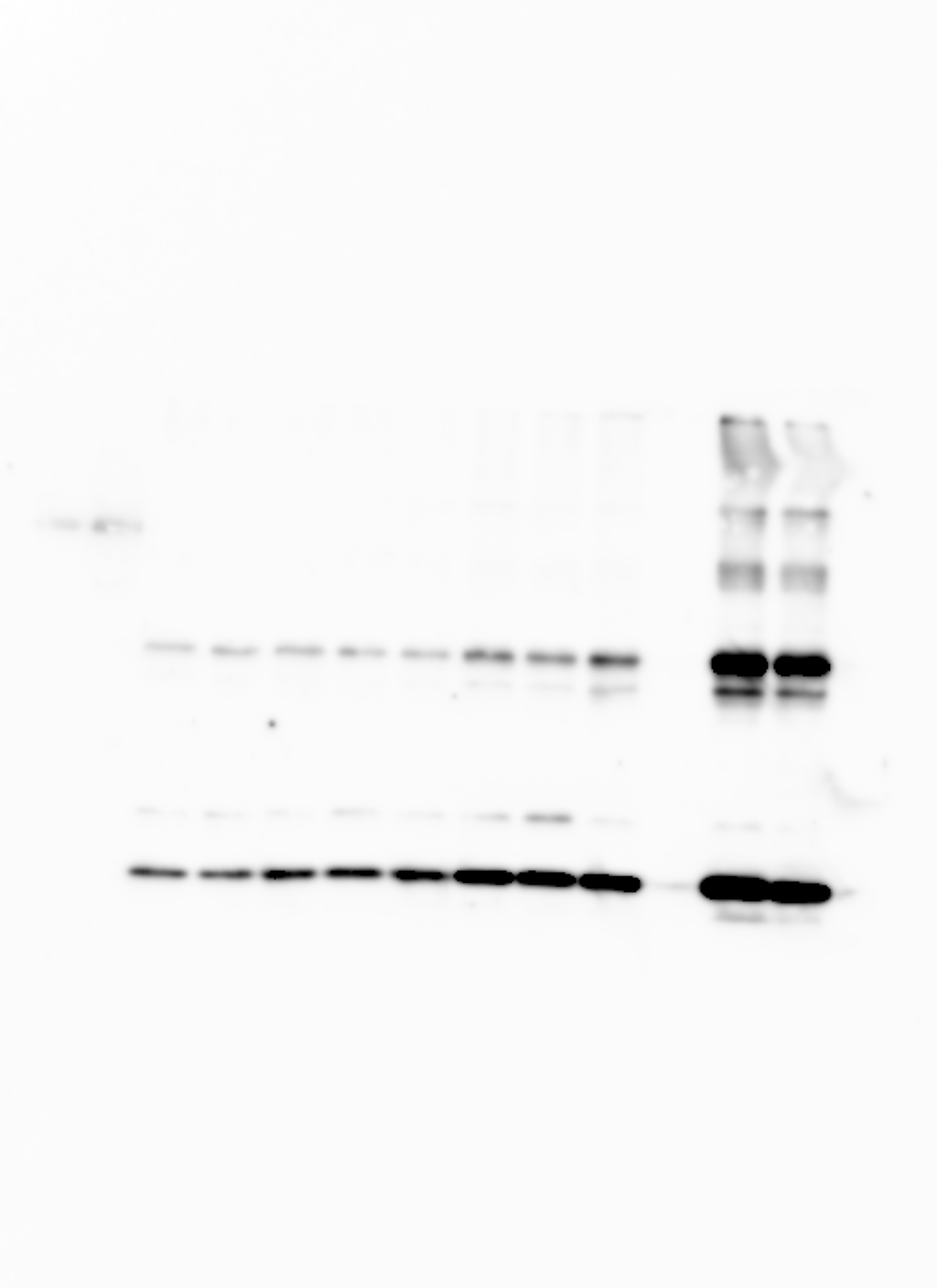

Supplement: Figure 5—figure supplement 1—source data 2. [file elife-75166-fig5-figsupp1-data2.zip › Figure_5-figure_supplement_2-Source_data_2/Fig5S1D_ASC_crosslink_raw.tif]

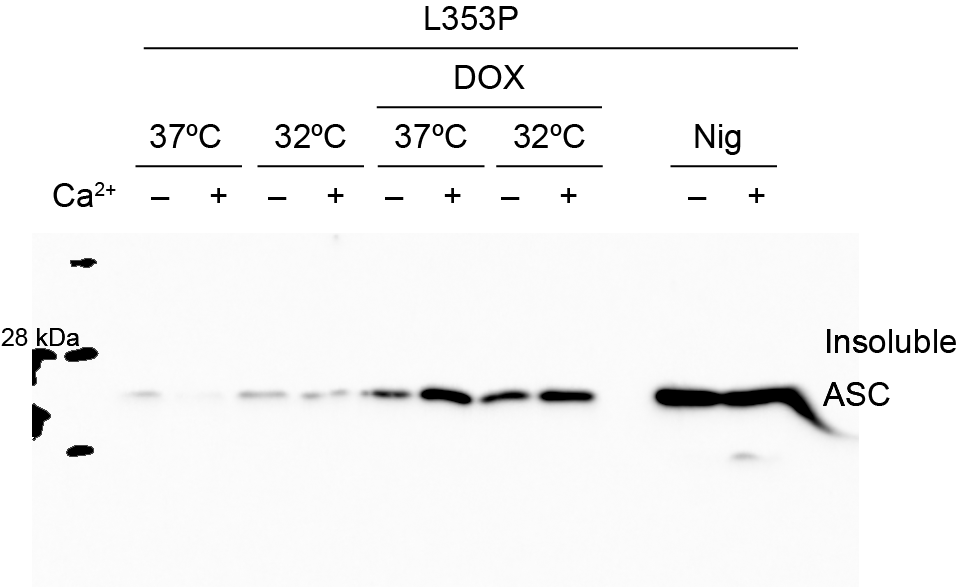

Supplement: Figure 5—figure supplement 1—source data 2. [file elife-75166-fig5-figsupp1-data2.zip › Figure_5-figure_supplement_2-Source_data_2/Fig5S1D_ASC_insoluble_labeled.tif]

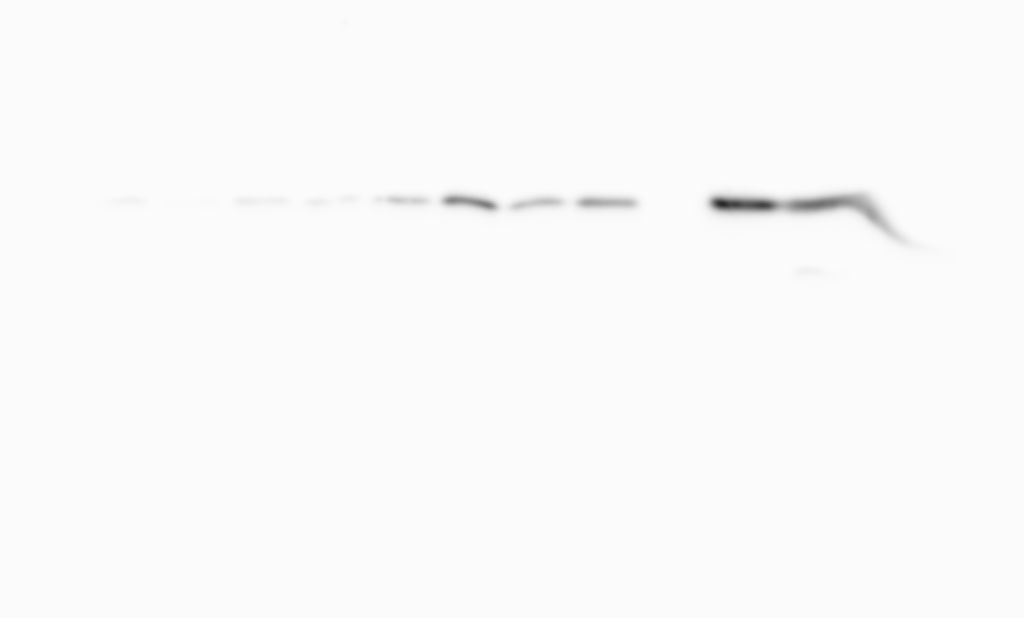

Supplement: Figure 5—figure supplement 1—source data 2. [file elife-75166-fig5-figsupp1-data2.zip › Figure_5-figure_supplement_2-Source_data_2/Fig5S1D_ASC_insoluble_raw.tif]

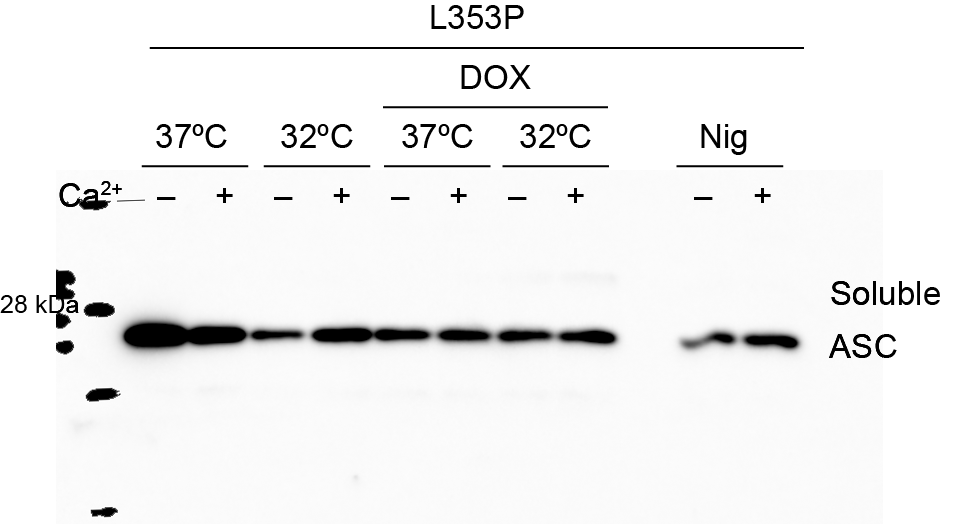

Supplement: Figure 5—figure supplement 1—source data 2. [file elife-75166-fig5-figsupp1-data2.zip › Figure_5-figure_supplement_2-Source_data_2/Fig5S1D_ASC_soluble_labeled.tif]

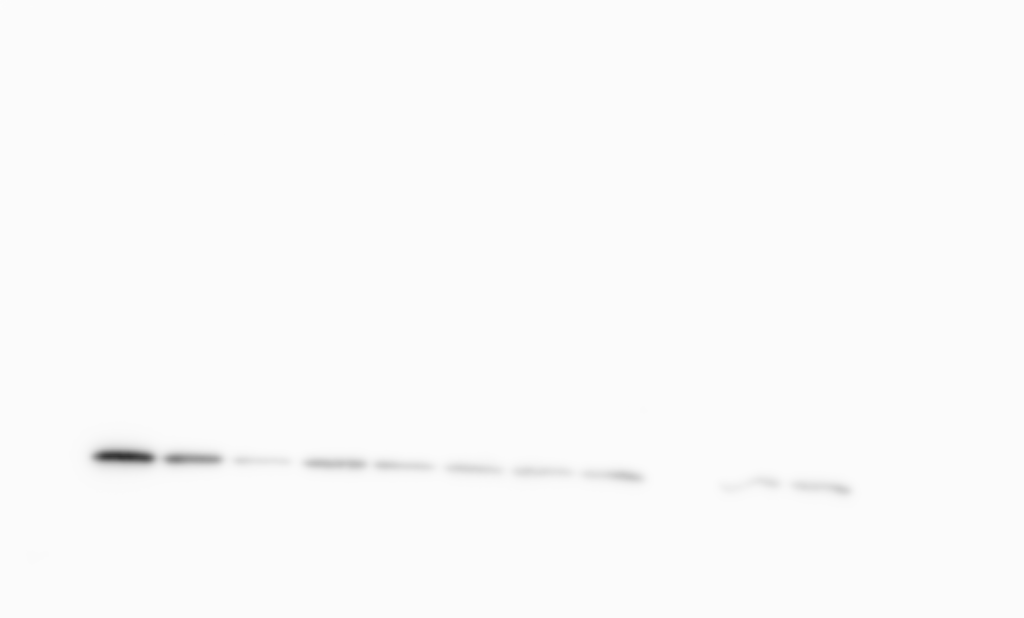

Supplement: Figure 5—figure supplement 1—source data 2. [file elife-75166-fig5-figsupp1-data2.zip › Figure_5-figure_supplement_2-Source_data_2/Fig5S1D_ASC_soluble_raw.tif]

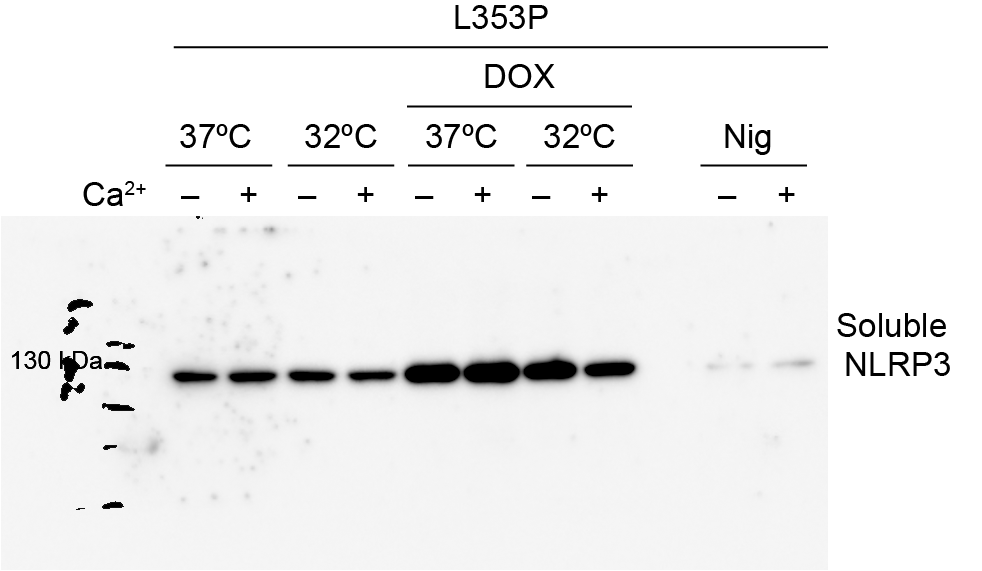

Supplement: Figure 5—figure supplement 1—source data 2. [file elife-75166-fig5-figsupp1-data2.zip › Figure_5-figure_supplement_2-Source_data_2/Fig5S1D_NLRP3_labeled.tif]

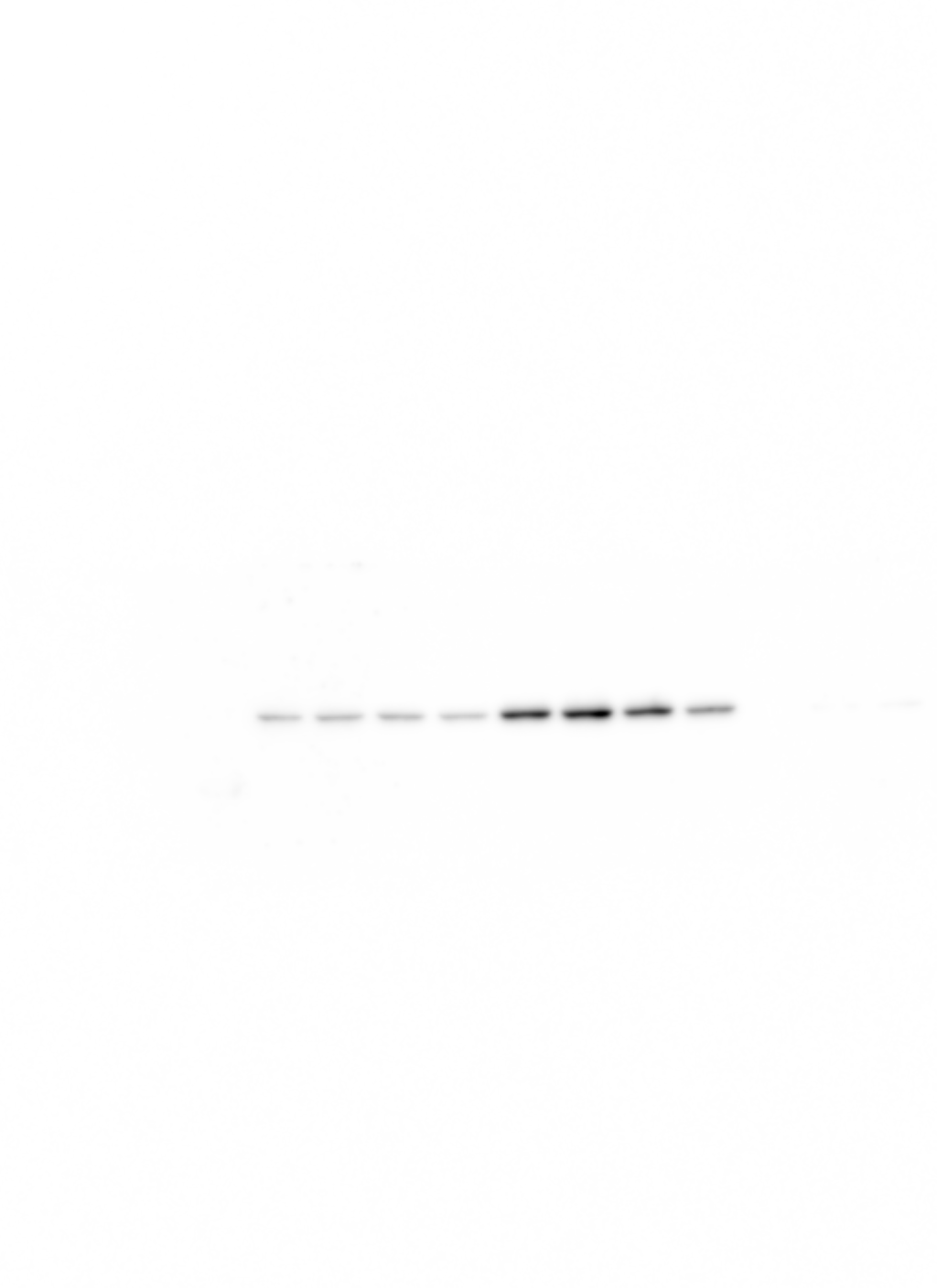

Supplement: Figure 5—figure supplement 1—source data 2. [file elife-75166-fig5-figsupp1-data2.zip › Figure_5-figure_supplement_2-Source_data_2/Fig5S1D_NLRP3_raw.tif]

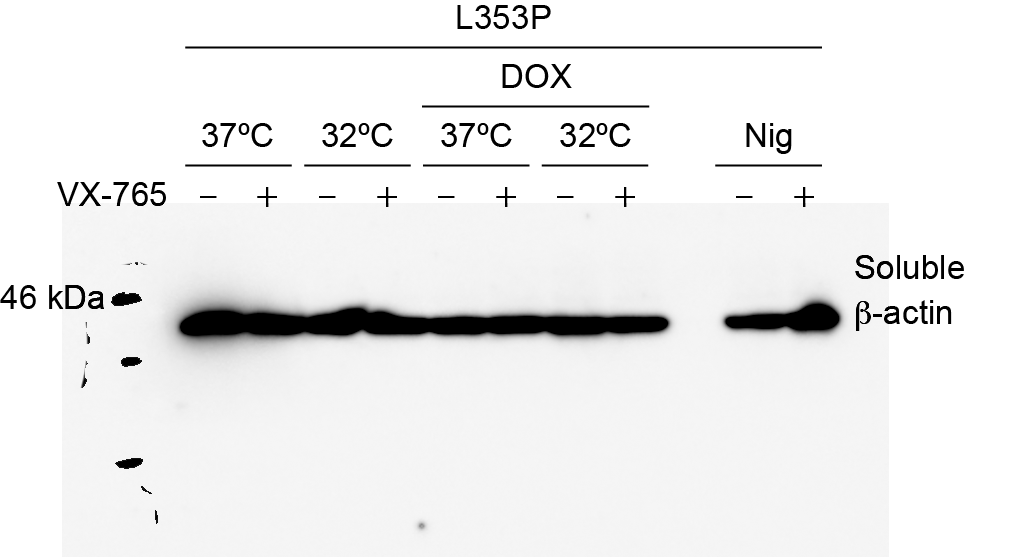

Supplement: Figure 7—source data 2. [file elife-75166-fig7-data2.zip › Figure_7-Source_data_2/Fig7D_actin_labeled.tif]

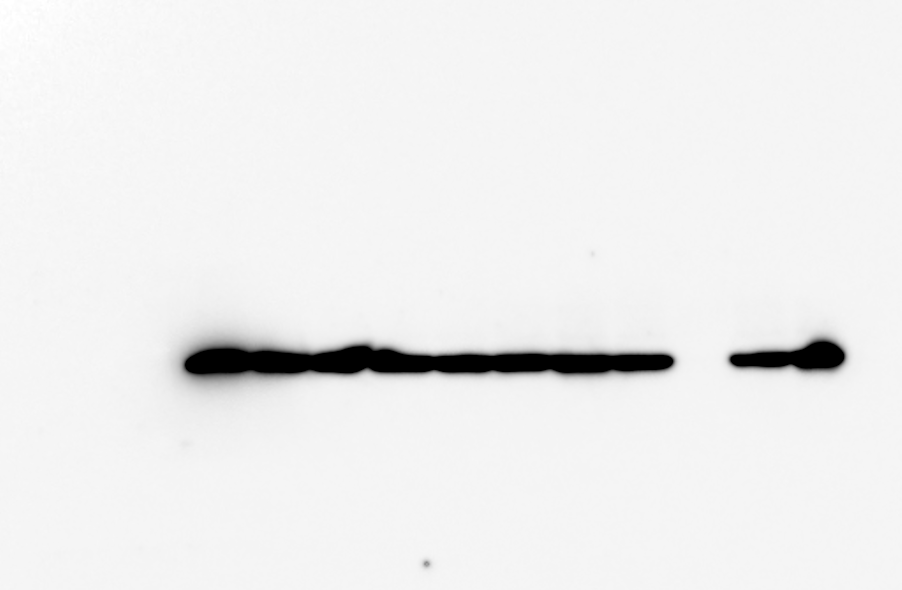

Supplement: Figure 7—source data 2. [file elife-75166-fig7-data2.zip › Figure_7-Source_data_2/Fig7D_actin_raw.tif]

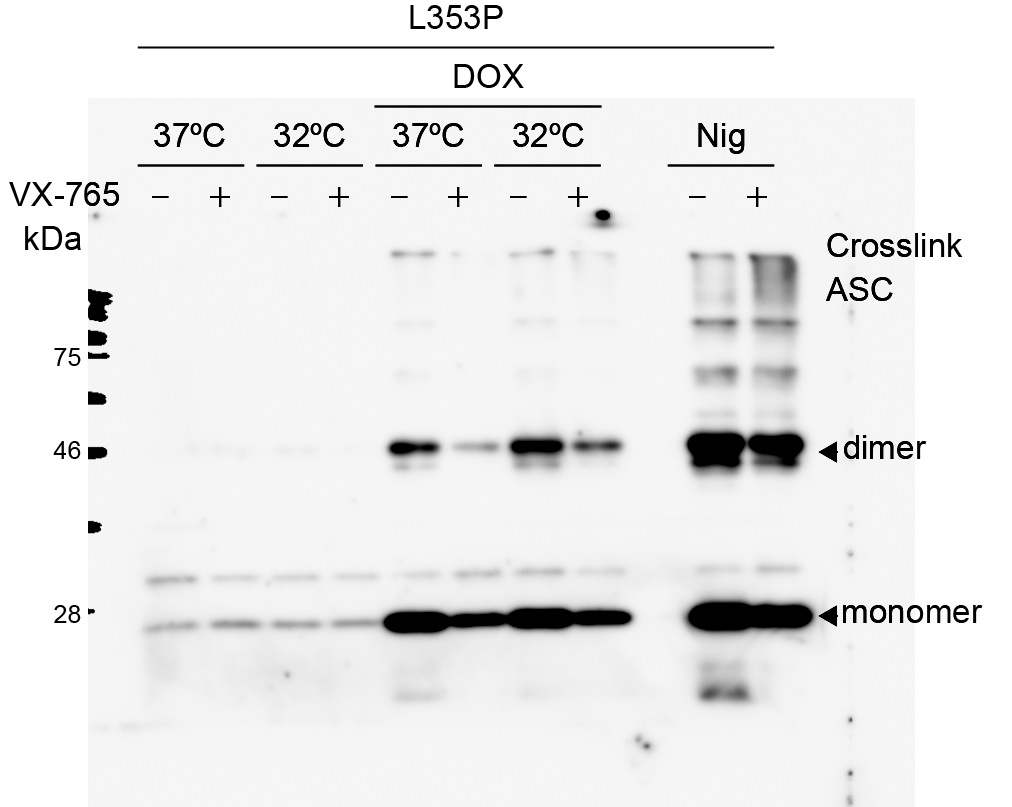

Supplement: Figure 7—source data 2. [file elife-75166-fig7-data2.zip › Figure_7-Source_data_2/Fig7D_ASC_crosslink_labeled.tif]

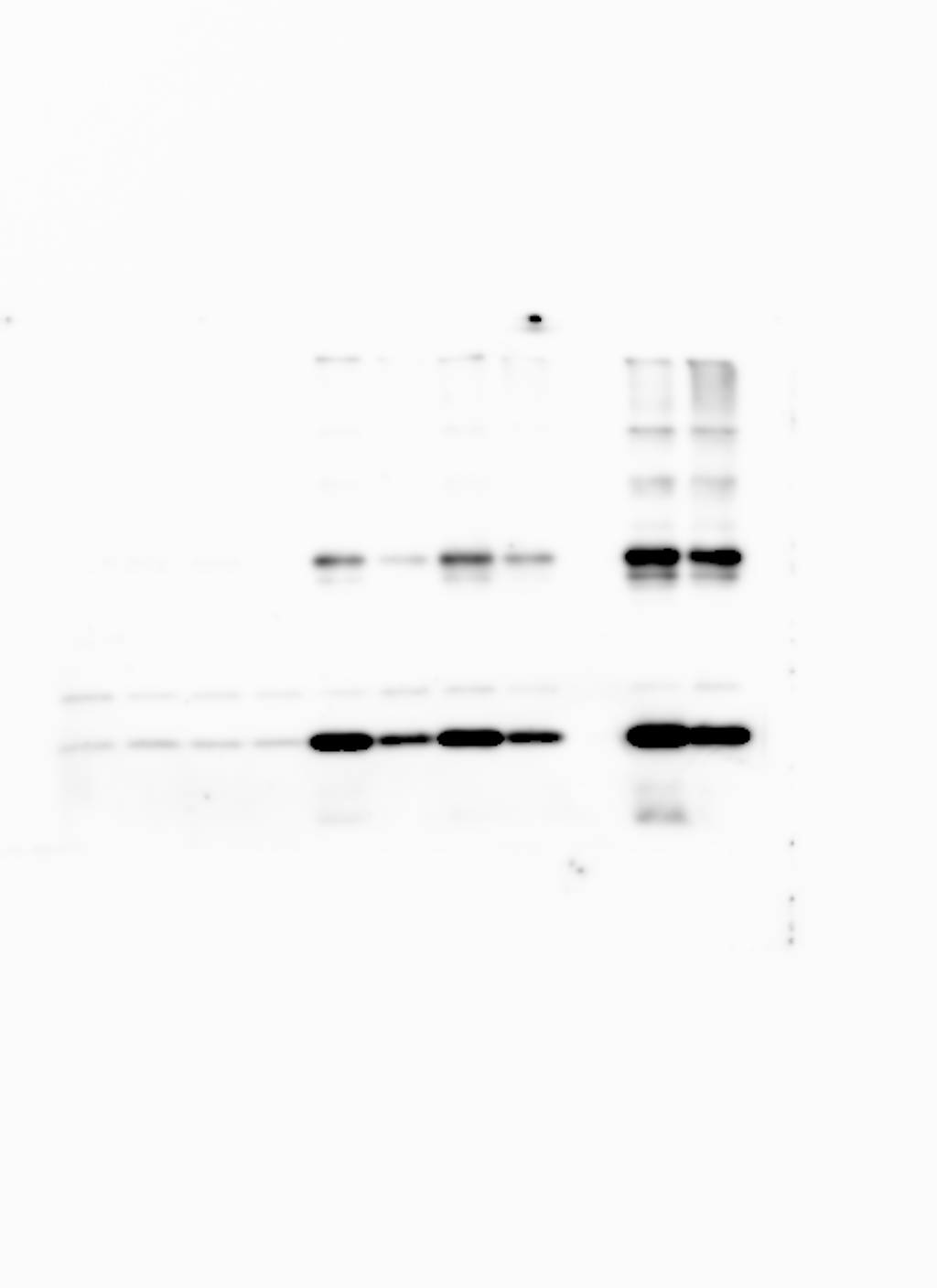

Supplement: Figure 7—source data 2. [file elife-75166-fig7-data2.zip › Figure_7-Source_data_2/Fig7D_ASC_crosslink_raw.tif]

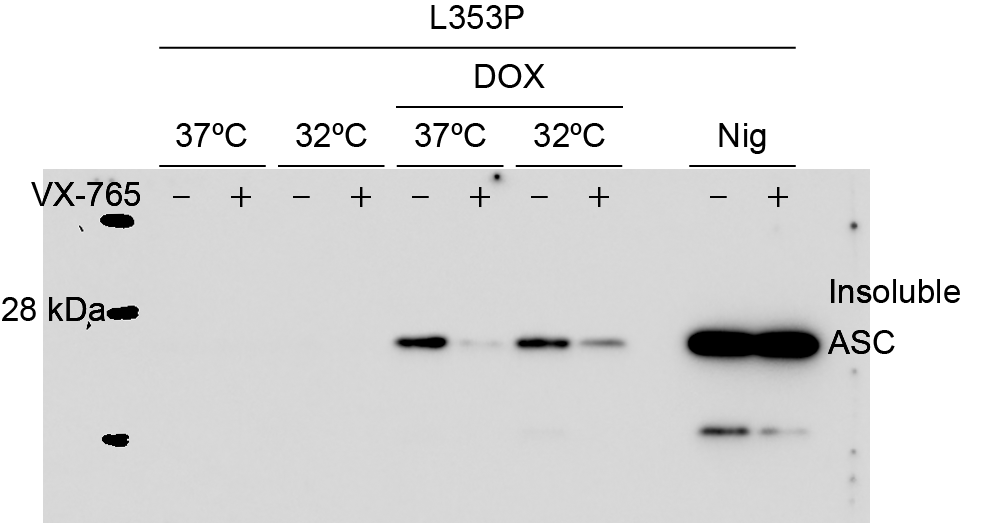

Supplement: Figure 7—source data 2. [file elife-75166-fig7-data2.zip › Figure_7-Source_data_2/Fig7D_ASC_insoluble_labeled.tif]

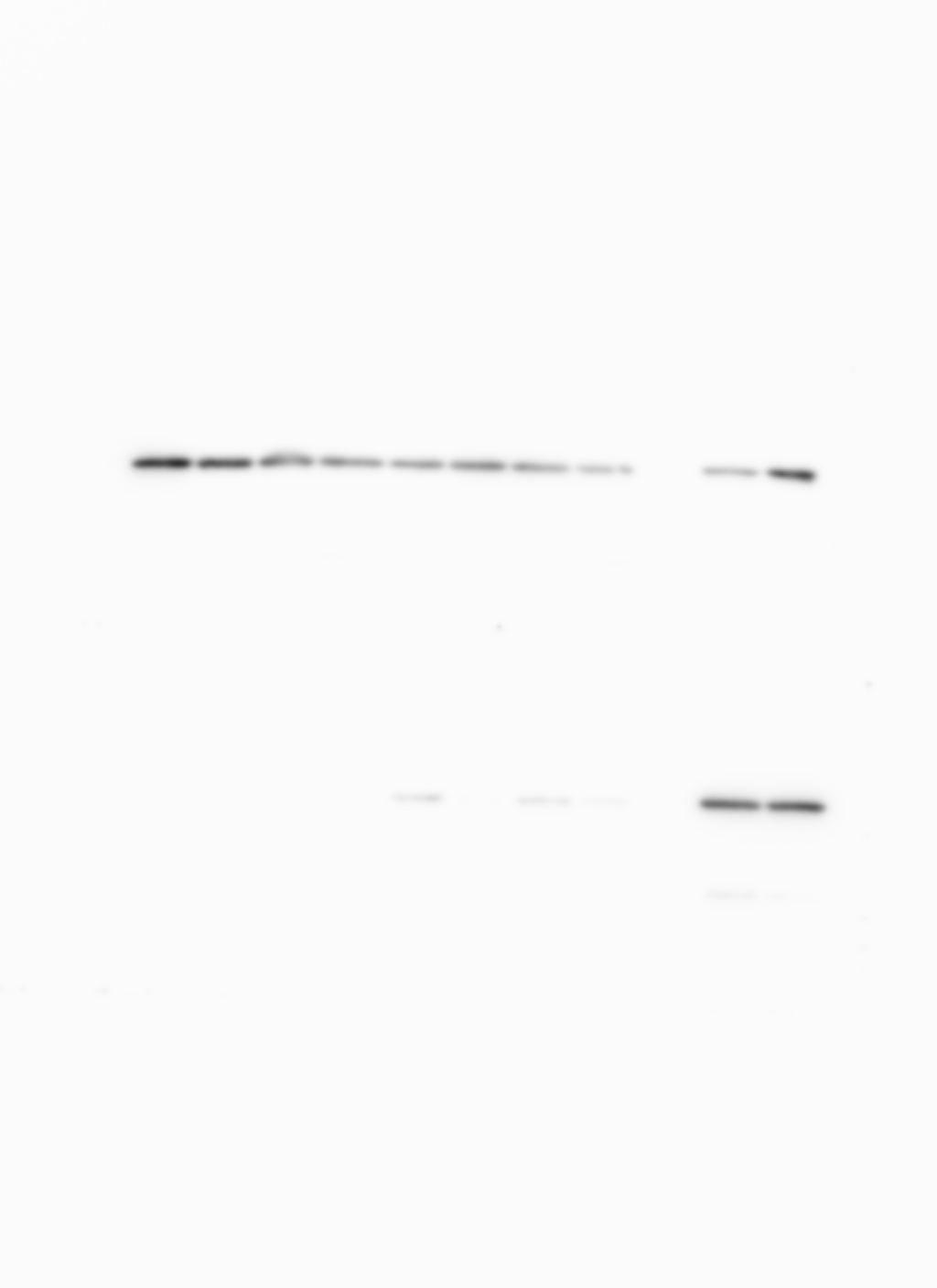

Supplement: Figure 7—source data 2. [file elife-75166-fig7-data2.zip › Figure_7-Source_data_2/Fig7D_ASC_soluble_insoluble_raw.tif]
