# Supplementary figures and images for: Cryo-sensitive aggregation triggers NLRP3 inflammasome assembly in cryopyrin-associated periodic syndrome (part 2 of 2)
Source: eLife. 2022 May 26;11:e75166. doi: 10.7554/eLife.75166 (PMC9177154; doi:10.7554/eLife.75166)

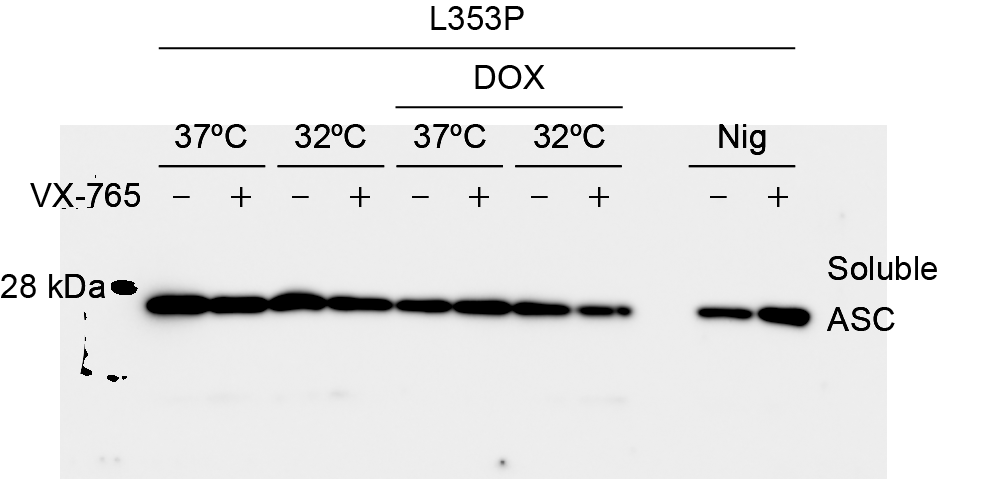

Supplement: Figure 7—source data 2. [file elife-75166-fig7-data2.zip › Figure_7-Source_data_2/Fig7D_ASC_soluble_labeled.tif]

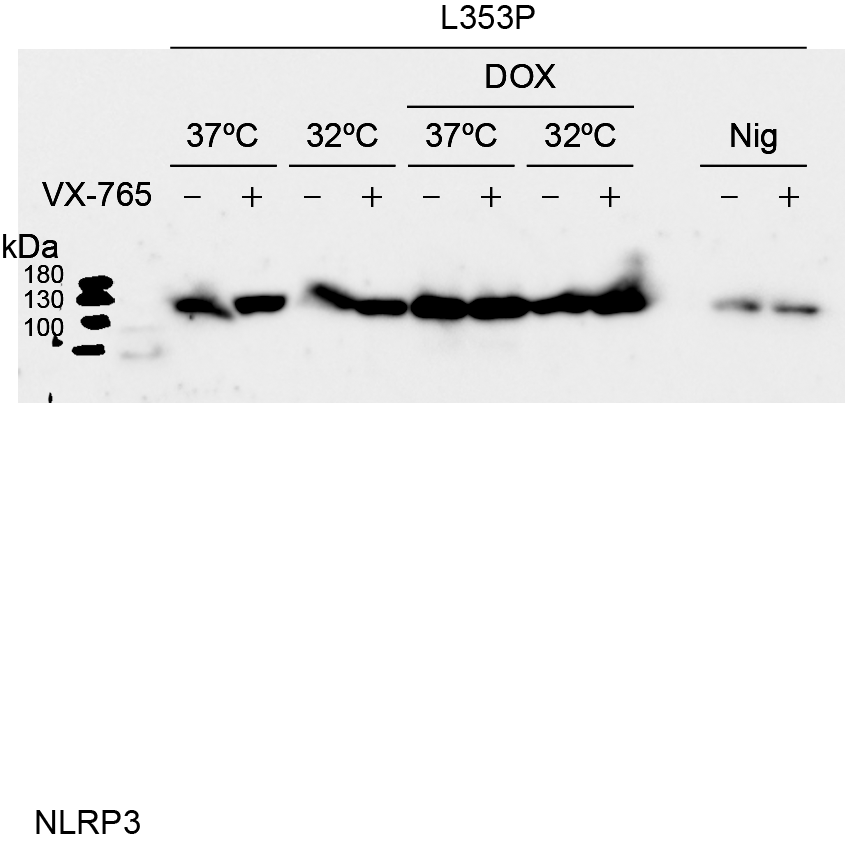

Supplement: Figure 7—source data 2. [file elife-75166-fig7-data2.zip › Figure_7-Source_data_2/Fig7D_NLRP3_labeled.tif]

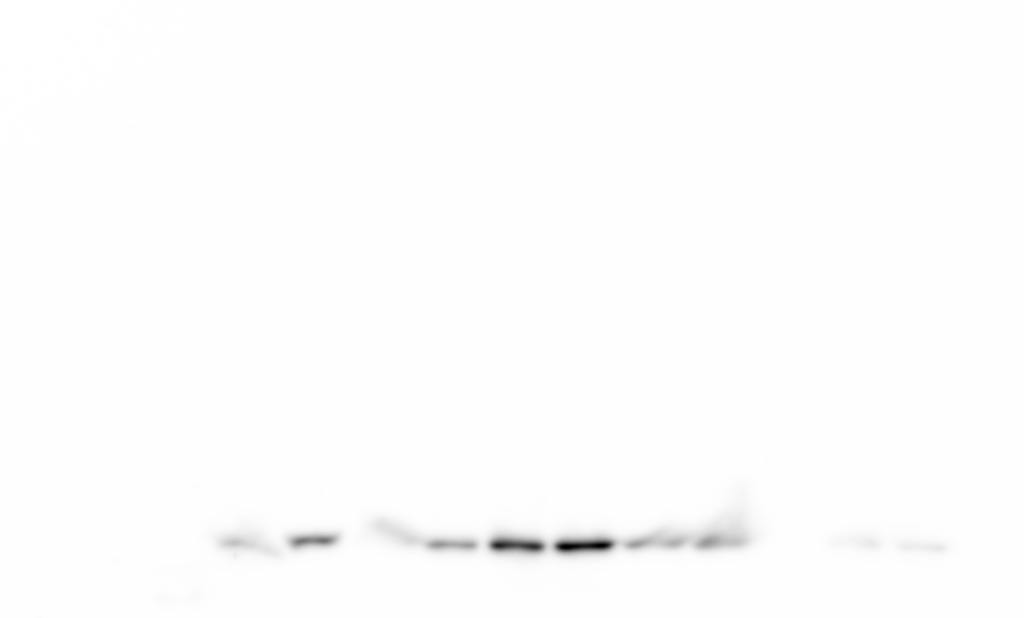

Supplement: Figure 7—source data 2. [file elife-75166-fig7-data2.zip › Figure_7-Source_data_2/Fig7D_NLRP3_raw.tif]

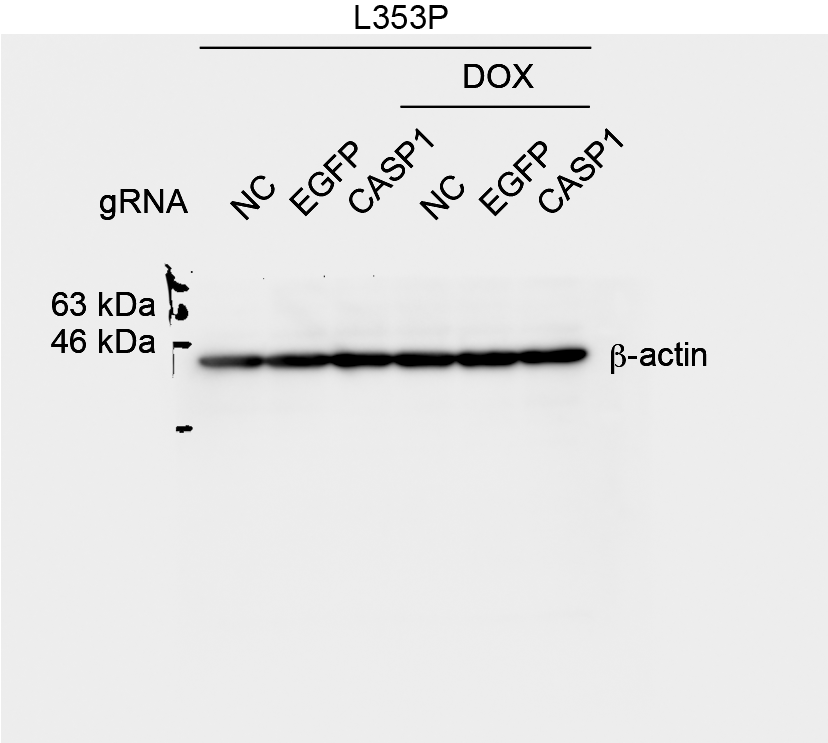

Supplement: Figure 7—figure supplement 1—source data 1. [file elife-75166-fig7-figsupp1-data1.zip › Figure_7ΓÇôfigure_supplement_1ΓÇôSource_data 1/Fig7S1D_actin_labeled.tif]

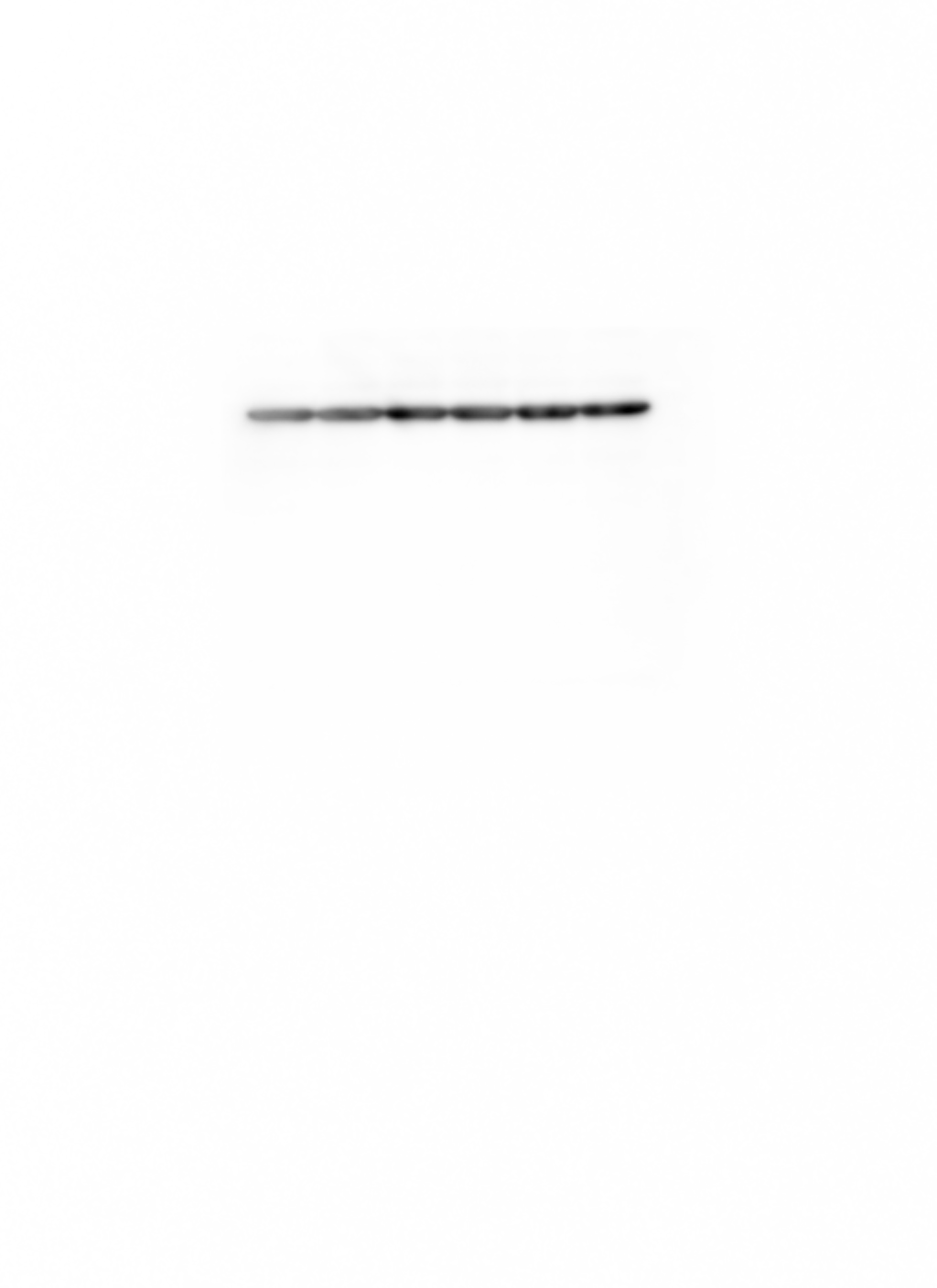

Supplement: Figure 7—figure supplement 1—source data 1. [file elife-75166-fig7-figsupp1-data1.zip › Figure_7ΓÇôfigure_supplement_1ΓÇôSource_data 1/Fig7S1D_actin_raw.tif]

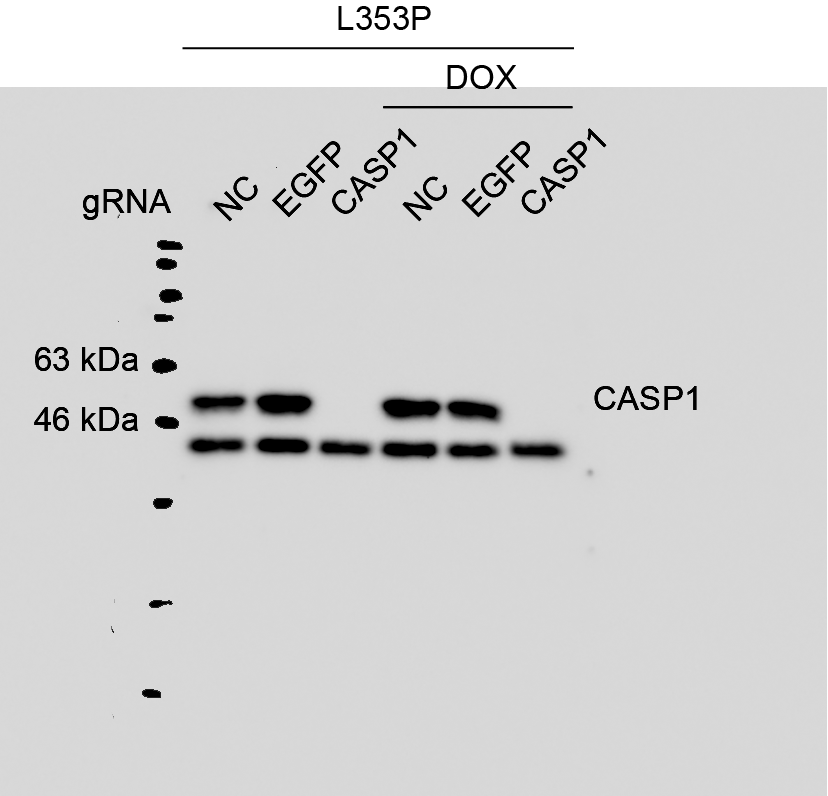

Supplement: Figure 7—figure supplement 1—source data 1. [file elife-75166-fig7-figsupp1-data1.zip › Figure_7ΓÇôfigure_supplement_1ΓÇôSource_data 1/Fig7S1D_CASP1_labeled.tif]

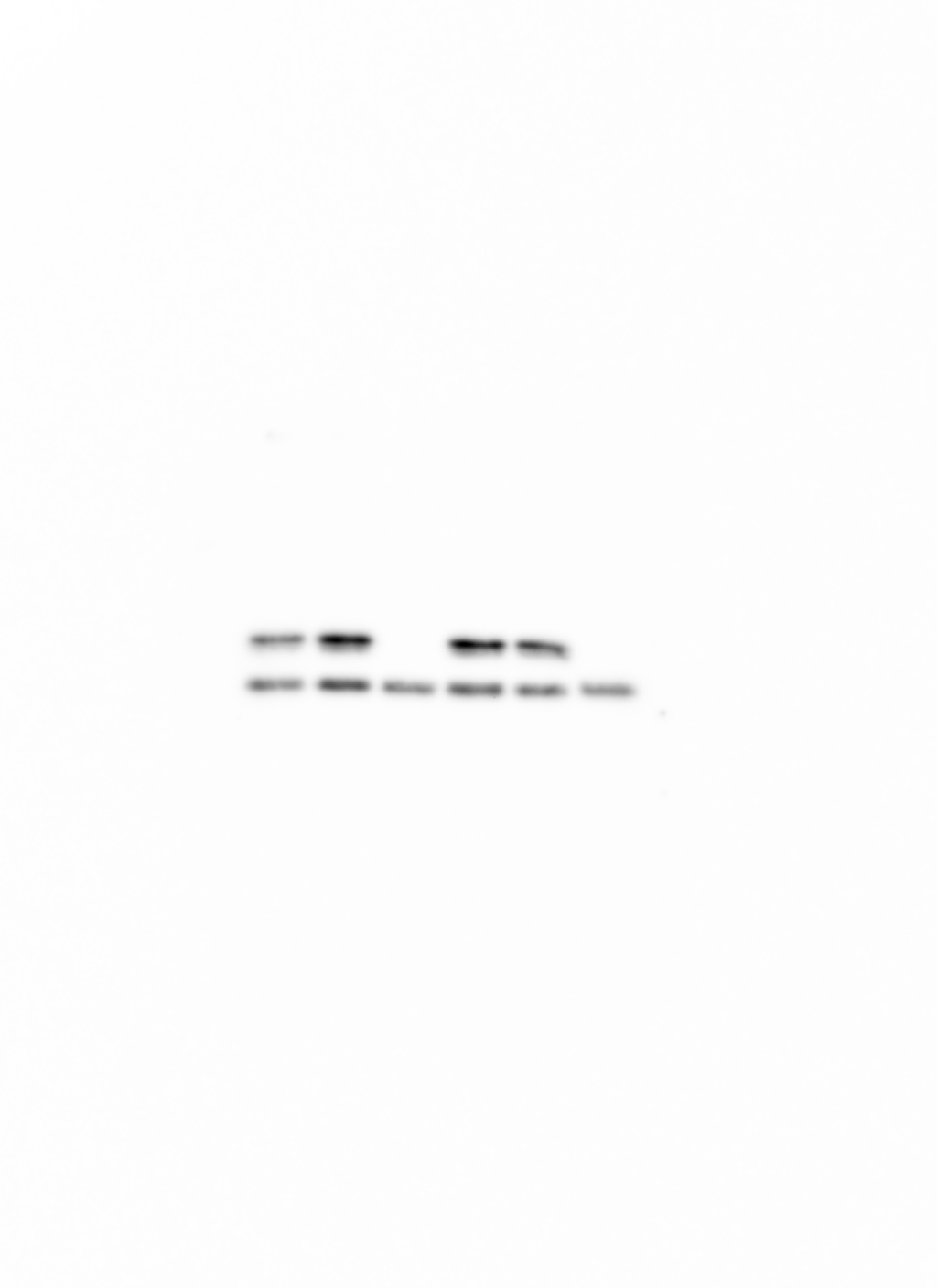

Supplement: Figure 7—figure supplement 1—source data 1. [file elife-75166-fig7-figsupp1-data1.zip › Figure_7ΓÇôfigure_supplement_1ΓÇôSource_data 1/Fig7S1D_CASP1_raw.tif]

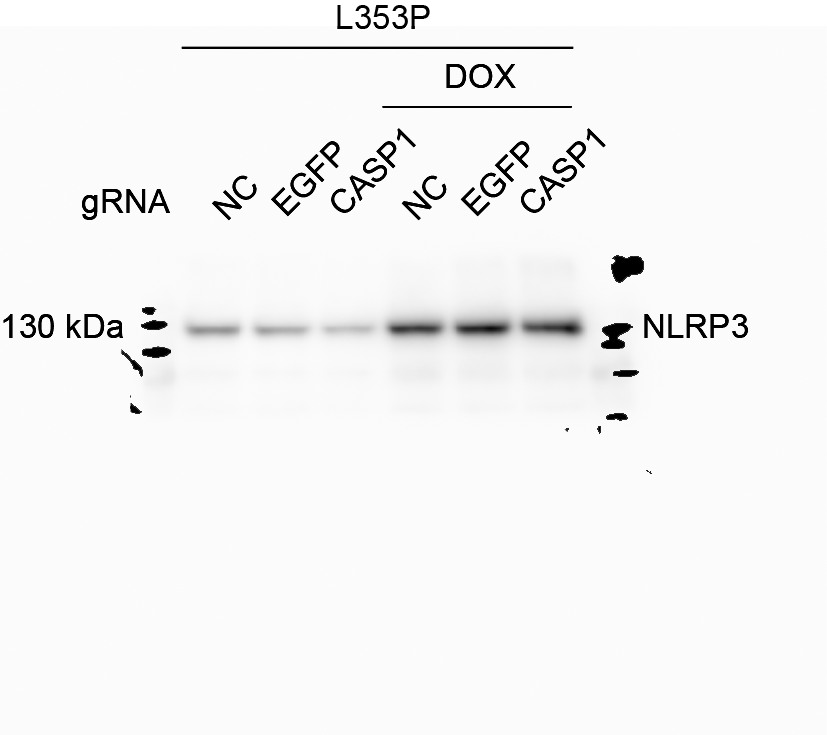

Supplement: Figure 7—figure supplement 1—source data 1. [file elife-75166-fig7-figsupp1-data1.zip › Figure_7ΓÇôfigure_supplement_1ΓÇôSource_data 1/Fig7S1D_NLRP3_labeled.tif]

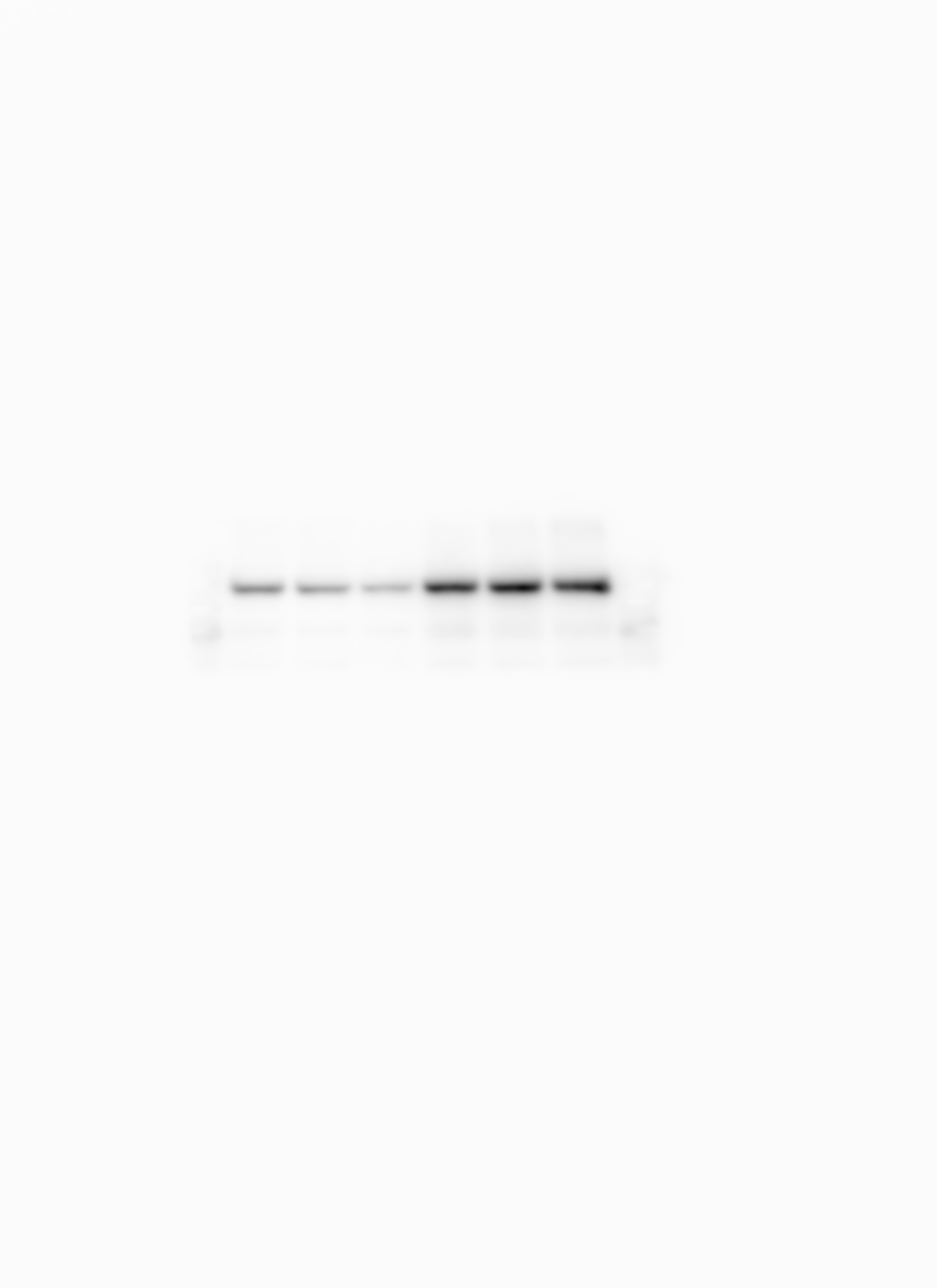

Supplement: Figure 7—figure supplement 1—source data 1. [file elife-75166-fig7-figsupp1-data1.zip › Figure_7ΓÇôfigure_supplement_1ΓÇôSource_data 1/Fig7S1D_NLRP3_raw.tif]
